# Photochemical synthesis of carbazole-fused Blatter radicals: effective spin injection to the carbazole system†

**DOI:** 10.1039/d5sc01182e

**Published:** 2025-05-27

**Authors:** Paulina Bartos, Patrycja Szamweber, Bruno Camargo, Anna Pietrzak, Piotr Kaszyński

**Affiliations:** a Faculty of Chemistry, University of Lodz 91-403 Lodz Poland piotr.kaszynski@cbmm.lodz.pl; b Centre of Molecular and Macromolecular Studies, Polish Academy of Sciences 90-363 Lodz Poland; c Institute of Experimental Physics, Faculty of Physics, University of Warsaw 02-093 Warsaw Poland; d Faculty of Chemistry, Lodz University of Technology 90-924 Lodz Poland; e Department of Chemistry, Middle Tennessee State University Murfreesboro Tennessee 37132 USA

## Abstract

Photocyclization of N-substituted carbazole derivatives of benzo[*e*][1,2,4]triazine gave two carbazole-fused Blatter radicals with a novel heterocyclic skeleton. No photocyclization was observed for the analogous dibenzocarbazole, indole, benzimidazole, and phenoxazine precursors, which was rationalized with DFT computational methods. The two carbazole-derived radicals were characterized by spectroscopic (UV-vis, EPR) and electrochemical methods, while one of them was analyzed structurally (XRD) and magnetically (SQUID). The latter analysis revealed ferromagnetic interactions in the solid state with 2*J*/*k*_B_ = 16.6 K. Properties of these first examples of a new class of stable radicals were analyzed with DFT methods, which confirmed significant impact of the *peri*-nitrogen atom on electronic properties and additional 15% spin delocalization.

## Abstract 2

Shining light on carbazole-benzotriazine derivatives leads to a new family of stable, π-delocalized radicals. The carbazole N atom has a significant impact on the electronic and magnetic properties of such planar Blatter radical derivatives.

## Graphical abstract

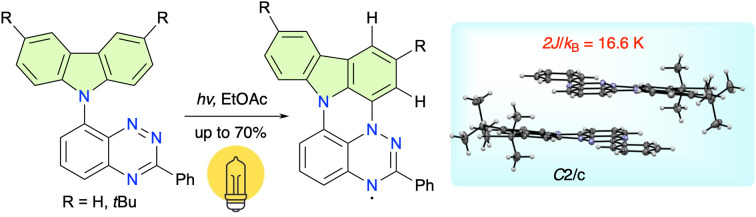

## Introduction

**Fig. 1:**
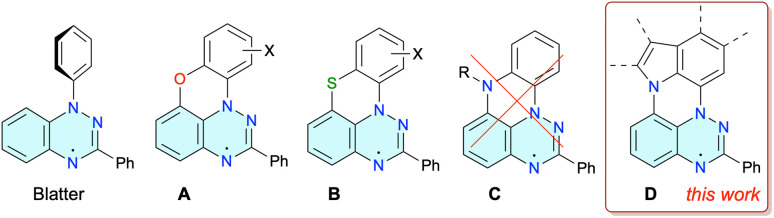
Structures of the Blatter radical and its planar analogues A–D.

Benzo[*e*][1,2,4]triazin-4-yl radicals, such as the prototypical Blatter radical^1^ (Fig. 1) and its derivatives,^2^ are becoming increasingly important structural elements for advanced functional materials.^3^ They have been explored in the context of organic batteries,^4^ molecular electronics,^5^ sensors,^6^ spintronics,^7^ high-spin materials,^8^ and photo-^9^ and ion-^10^ conductive liquid crystals. Applications of benzo[*e*][1,2,4]triazin-4-yls exploiting their stability and redox and magnetic properties in other areas of modern material science, such as information processing,^11^ flexible electronics,^12^ organic emitters^13^ and bioimaging,^14^ still await the development of suitable derivatives and new synthetic methods.

Recent advances^2^ in the chemistry of the benzo[*e*][1,2,4]triazinyl radicals led to the discovery^15^ of oxygen and sulfur *peri*-annulated Blatter radicals A and B (Fig. 1).^16^ Planarization of the radicals affects primarily their packing properties in the solid state, improves spin delocalization, and lowers the optical band-gap. On the basis of general trends in polyaromatic hydrocarbons^17^ and DFT calculations,^18^ it was expected that the nitrogen atom in the analogous N-*peri*-annulated planar Blatter radicals C would have an even greater impact on their photophysical, electrochemical and magnetic properties.

Recent investigations^18,19^ on access to N-*peri*-annulated Blatter radicals of the general structure C through aza-Pschorr,^18^ photocyclization^18^ and classical methods^19^ demonstrated that the desired radicals are possibly formed as transient species before undergoing either homolytic R–N fragmentation (R = Me, Ac in Fig. 1)^18^ or aerial oxidation (R=H)^19^ and formation of zwitterionic products. It was postulated^18^ that the homolysis of C with R = Me, Ac is a mildly endergonic process accessible at ambient temperature, while stable radicals C could be obtained for less thermodynamically stable R˙ (high homolytic bond dissociation energy, HBDE, of R–H), such as R = Ph (Fig. 1).^18^ However, even higher stability of N-*peri*-annulated Blatter radicals can be expected for systems in which the R = aryl substituent is ring-fused as in series D (Fig. 1). A particularly attractive member of this series could be derived from carbazole, a building block for materials with favorable electrochemical and photophysical properties.^20^ In the context of the latter, there is a rapidly growing interest in photophysics of carbazole derivatives substituted with stable radicals.^21^

Herein, we report the first N-*peri*-annulated Blatter radicals 1 (series D, Fig. 1) obtained by using the recently developed photocyclization method^22^ of appropriate benzo[*e*][1,2,4]triazines 2. Two carbazole-based radicals, 1c and 1d, are characterized by spectroscopic (UV-vis and EPR) and electrochemical methods. The solid-state structure of radical 1d and oxidation product of 1c are investigated with single crystal XRD, while magnetic properties of the former radical are analyzed with SQUID magnetometry methods. The experimental data are augmented with density functional theory (DFT) computational results. The successful photocyclization of 2c and 2d, but not other analogous precursors 2, is rationalized with extensive DFT analysis.

## Results and discussion

### Synthesis

**Scheme 1:**
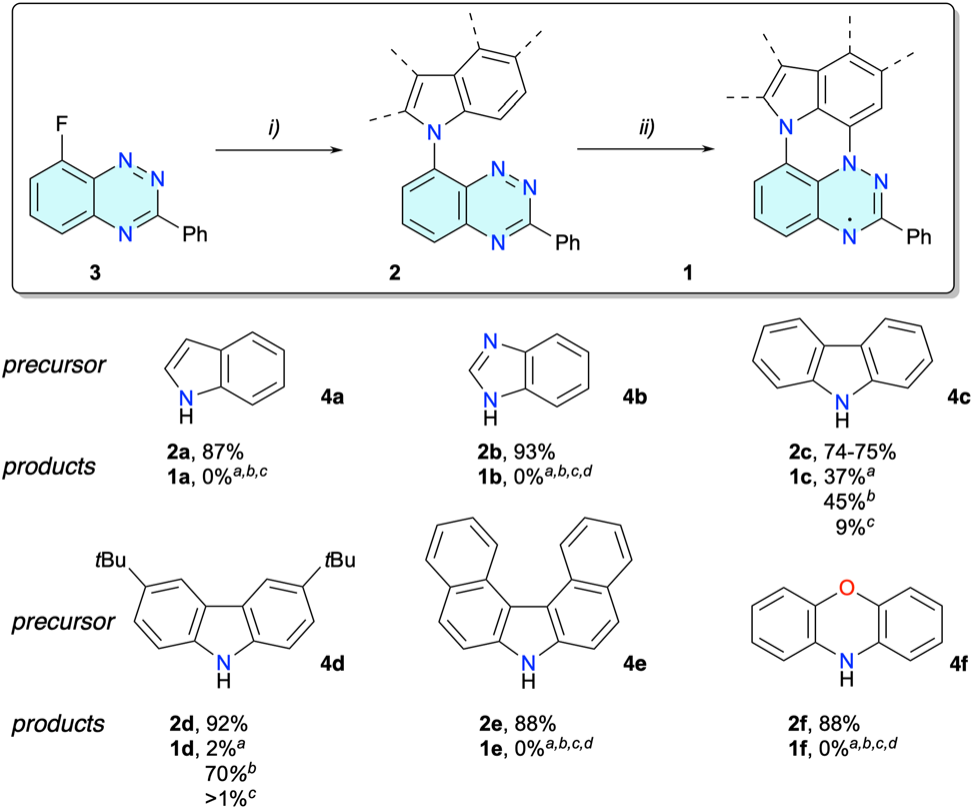
Synthesis of benzo[*e*][1,2,4]triazines 2 and their photocyclization to radicals 1 at *c* ≈ 1 mM. ^*a*^ Reagents and conditions: (i) heteroarene 4a–4f, 60% NaH, dry DMSO, 80 °C, 3 h; (ii) 300 W halogen lamp in a Pyrex flask, 1 mM (a) in CH_2_Cl_2_, (b) in EtOAc or (c) in EtOH, 30–35 °C, 72 h; (d) no reaction.

**Fig. 2:**
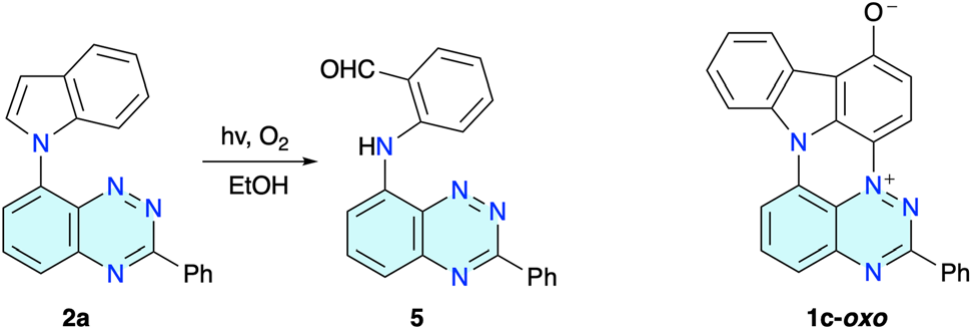
Left: photooxidation of indole derivative 2a in EtOH (*c* ≈ 1 mM). Right: the structure of byproduct 1c-oxo.

Initial investigation focused on photocyclization of the indole derivative 2a, prepared in 87% yield from fluoro derivative^15^3 and indole (4a) in DMSO in the presence of NaH (Scheme 1). Thus, irradiation of diluted solutions (*c* ≈ 1 mM) of 2a in CH_2_Cl_2_ or EtOAc with a 300 W halogen lamp through a Pyrex filter gave no reaction and starting 2a was recovered after 72 h. In contrast, the same photoreaction of 2a conducted in EtOH gave a complex mixture of products, as evident from multiple color spots in the thin layer chromatography (TLC). Mass spectrometry analysis of the reaction mixture suggested the formation of trace amounts of radical 1a, while the main product isolated in 53% yield was identified as benzaldehyde 5, formed, presumably, by oxidative ring opening in 2a with singlet oxygen (Fig. 2). Oxidative cleavage of the C(2)–C(3) double bond in indole either by ozone^23^ or by microorganisms^24^ has been reported. To prevent this degradation process, the indole substituent in 2a was modified by replacing the C(3′) with N(3′) in benzimidazole derivative 2b, and by fusing a benzene ring in carbazole 2c. Attempted photocyclization of the former failed in all three solvents, and it was recovered unreacted. On the other hand, the carbazole precursor 2c was more promising.

Irradiation of 2c in CH_2_Cl_2_ resulted in partial consumption of the starting material (50%) and formation of product 1c isolated in 37% yield (Scheme 1). Changing the reaction medium to EtOAc resulted in full conversion of the starting 2c after 72 h, and the desired radical 1c was isolated as a high-melting (>250 °C) brown/purple solid in 45% yield. The photocyclization performed in EtOH was less efficient and 1c was isolated in 9% yield along with starting 2c recovered in 70% yield.

The formation of radical 1c was accompanied by a more polar byproduct, which was isolated by chromatography. Extensive spectroscopic and XRD (*vide infra*) analysis revealed that the polar blue product is zwitterion 1c-oxo isolated in about 12% yield from the reaction mixtures in EtOH and CH_2_Cl_2_ (Fig. 2). Extending the irradiation time did not significantly affect the yields of 1c presumably due to the increased optical density of the solutions and promoted oxidation of radical 1c to 1c-oxo.

The formation of 1c-oxo was attributed to the reaction of radical 1c with molecular oxygen at the CH site with the highest spin density (*vide infra*). A similar process was observed for the parent Blatter radical, which undergoes oxidation at the C(7) position with high spin density and forms a polar quinoimine product.^25^ To prevent aerial oxidation of radical 1c, the carbazole was substituted with *t*-Bu groups in derivative 2d (steric protection) and fused with two benzene rings in derivative 2e (substitution protection). Irradiation of derivative 2d in EtOAc resulted in full consumption of the starting material and formation of radical 1d isolated in 70% yield. Interestingly, reactions of 2d performed in CH_2_Cl_2_ or EtOH gave only traces, less than 2% yield of the desired 1d, with the recovery of starting 2d in 95–98% yield. In contrast, dibenzocarbazole 2e was inert under irradiation conditions in all three solvents and was fully recovered even after 7 days of irradiation. Similarly, phenoxazine precursor 2f was inert under these reaction condition in all three solvents.

The high effectiveness of the *t*-Bu groups in stabilization of radical 1c against oxygen was demonstrated by monitoring of low energy absorption bands of radicals 1c and 1d in solutions exposed to air. Results showed that while the parent radical 1c decays with a pseudo first-order rate constant of 0.0211(5) h^−1^ (*τ*_1/2_ = 1.37 d), the *t*-Bu substituted derivative 1d shows significantly slower decay, *k* = 7.9 × 10^−4^ h^−1^, with a half-life of about 37 d (for details see the ESI†).

### Molecular and crystal structures

**Fig. 3:**
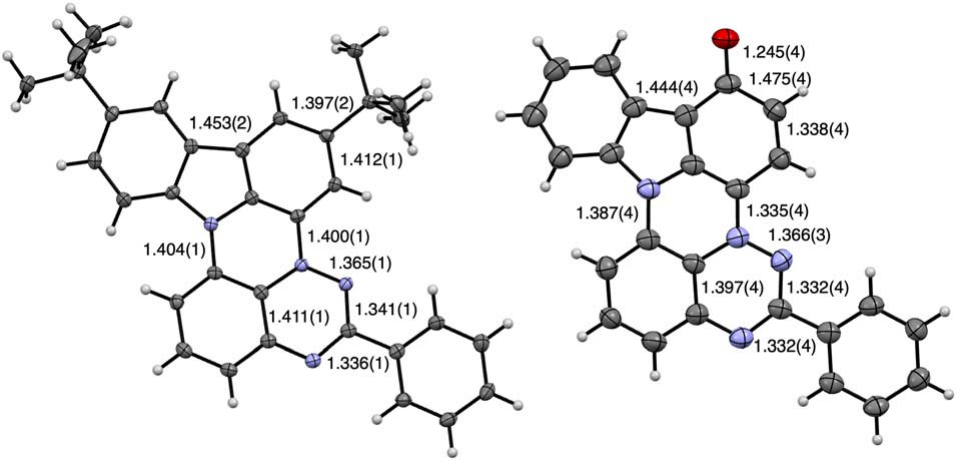
Molecular structures of 1d (left) and 1c-oxo “donor” molecule (right) with pertinent interatomic distances. Atomic displacement ellipsoids are drawn at 50% probability level. N atoms are in blue. For the numbering system see Fig. 4. See text and ESI† for details.

**Fig. 4:**
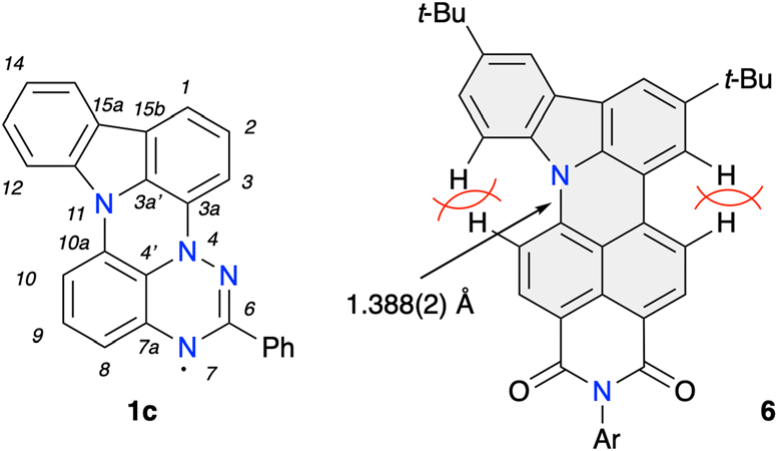
Left: partial numbering system for 6-phenyl-7*H*-indolo[3,2,1-*de*][1,2,4]triazino[5,6,1-*kl*]phenazin-7-yl (1c). Right: structure of closely related indolo[3,2,1-*de*]acridine system 6 with the indicated key interatomic distance (ref. 26).

**Fig. 5:**
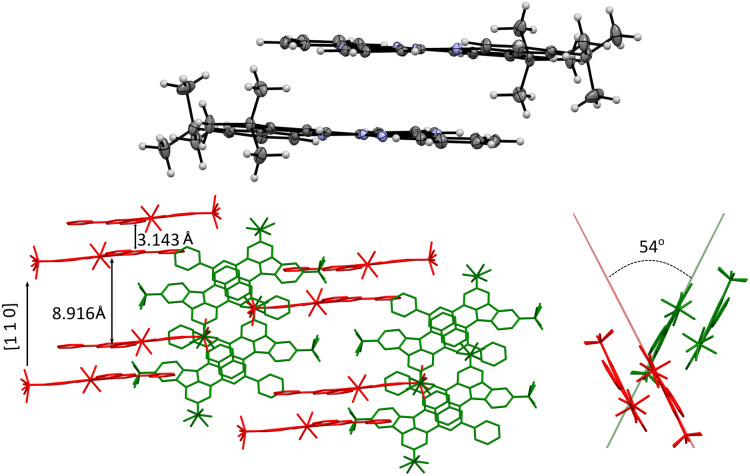
Top: discrete π⋯π dimer of 1d. Bottom: two views of partial crystal packing of 1d with discrete dimers shown in two relative orientations (red and green).

**Fig. 6:**
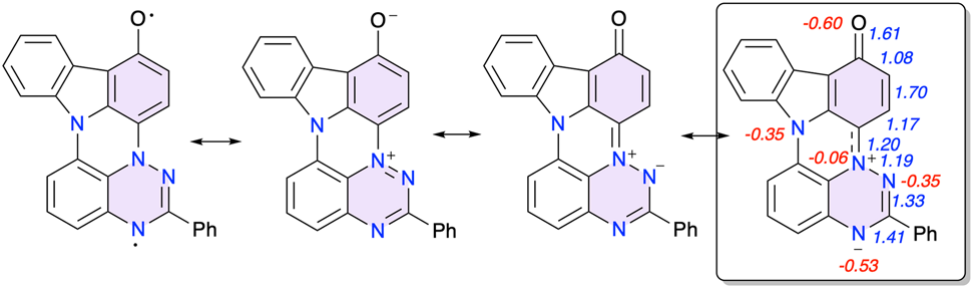
Selected resonance forms of 1c-oxo with the dominant in the box. Wiberg Bond Index (WBI) values are shown in blue and natural charges n*q* (e^−^ units) for heteroatoms in red.

**Fig. 7:**
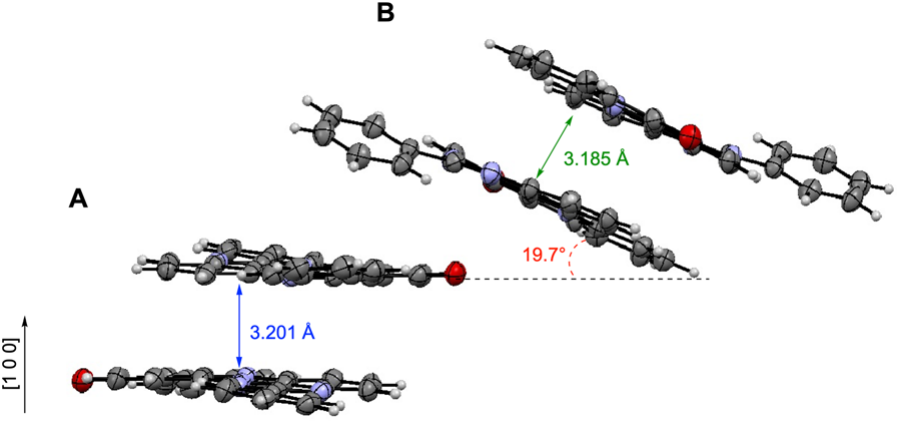
Partial crystal packing of 1c-oxo showing arrangements of two unique molecules A and B.

Dark purple needle-shaped crystals of 1d and dark blue microcrystals of 1c-oxo suitable for single crystal XRD analysis were obtained by slow evaporation of MeCN/CH_2_Cl_2_ solutions. Both compounds crystallize in a monoclinic system adopting the *C*2/*c* and *P*2_1_/*n* space groups, respectively. The structure of radical 1d contains one molecule with rotationally disordered *t*-Bu groups in the asymmetric unit, while in the 1c-oxo there are two symmetry independent molecules. The results are shown in Fig. 3–7, and full data are provided in the ESI.†

Analysis of the molecular structure of 1d demonstrates that this novel heterocyclic system formally contains two fused subunits, carbazole and benzo[*e*][1,2,4]triazine, connected with N(11)–C(10a) and N(4)–C(3a) bonds of 1.404(2) and 1.400(1) Å, respectively (Fig. 3 and 4). The former distance is comparable to 1.388(2) Å for the analogous C–N bond found in compound 6,^17*c*^ the closest known^17*c*,26^ structural analogue of 1d (Fig. 4). The N(4)–C(3a) bond length in 1d is essentially the same as the analogous distance in oxo derivatives A (1.399(3)–1.405(2) Å)^16*b*^ and shorter than that found in the sulfur analogue B(X=H) (1.420(3) Å)^15^ and Blatter (1.427(2) Å).^27^

Both subunits in 1d, the carbazole and benzo[*e*][1,2,4]triazine, are twisted relative to each other by *θ* = 6.6° due to through space C(10)-H⋯H–C(12) interactions. The analogous dihedral angle between the planes defined by carbazole and naphthalene subunits in 6 is 12.8°, resulting from two through space H⋯H interactions.^26^ The Ph group in 1d is almost coplanar with the benzo[*e*][1,2,4]triazine subunit forming a 2.9° twist angle.

In the crystal, molecules of 1d form discrete (non-covalent) π⋯π dimers with a mean interplanar distance of 3.143 Å (Fig. 5). The dimers are arranged in two stacks: one along the [1 1 0] direction (red in Fig. 5) and the other rotated by about 54° (green). The *t*-Bu groups separate the dimers in the neighboring stacks, which results in the interdimer distance of 8.916 Å. The primary close nonbonding contacts within the dimer are C(6)⋯C’(10a), 3.288 Å (0.112 Å inside the Van der Waals, *V*d*W*, distance) and C(8)⋯C′(3), 3.322 Å (0.078 Å inside the *V*d*W* separation), which correspond to interactions of sites with opposite spin densities and indicate ferromagnetic interactions between the two radicals (for the numbering system see Fig. 4). In addition, there are nonbonding contacts between the heterocycle C(3a′) and Me carbon atoms (3.286 Å, 0.114 Å inside VdW separation).

Two symmetry independent molecules in the crystal structure of 1c-oxo (Fig. 3) are nearly planar with the Ph group rotated about 5.9° and 22.4°, respectively, relative to the mean plane of the heterocycle. A comparison of the molecular structure of 1d and 1c-oxo demonstrates that oxidation of the C(1) position affects mainly the oxidized benzene ring connected to the N(4) position, and to a lesser extent the triazine ring. Thus the C(1)–C(2), C(3)–C(3a), and C(1)–C(15b) distances are significantly expanded, while the C(2)–C(3) and C(3a)–N(4) contracted to different extent in both unique molecules of 1c-oxo upon oxidation of 1c. These changes in bond lengths indicate the iminoquinone structure, which is also consistent with the short C–O bond (avg 1.246(4) Å) characteristic for C=O in quinones (1.222(13) Å).^28^

Close inspection of the unit cell shows that the two unique molecules of 1c-oxo have different environments: the oxygen atom of one molecule (“donor”) has short contacts with the positively charged N(4) (3.017(3) Å, 0.053 Å inside *V*d*W*) and C(3a) (3.152(4) Å, 0.068 Å inside *V*d*W*) atoms of the second molecule (“acceptor”). This transfer of electron density from the “donor” to “acceptor” results in a markedly longer C(3a)–N(4) distance, 1.360(4) Å, in the “acceptor” than in the “donor” (1.335(4) Å).

The assignment of the iminoquinone structure to 1c-oxo is corroborated by DFT calculations and NBO population analysis. Results shown in Fig. 6 demonstrate increased Wiberg bond index (WBI) value for C(1)–O, C(2)–C(3) and decreased for C(1)–C(2) and C(3)–C(3a), relative to the typical value of WBI = 1.4 for an aromatic system, which is consistent with the quinoid structure. Analysis of natural charges, n*q*, in 1c-oxo revealed a significantly increased at N(7) (n*q*_N(7)_ = −0.53*e*) and significantly reduces at N(4) (n*q*_N(4)_ = −0.06*e*) negative charge relative to the typical n*q*_N_ = −0.35*e* at N(5) and N(11). Further DFT calculations indicated that the closed shell singlet is the ground state of 1c-oxo with the triplet lying 21.2 kcal mol^−1^ above it.

Each unique molecule of 1c-oxo is arranged in discrete π⋯π dimers with an interplanar distance of 3.201 Å for the “donor” (molecule A) and 3.185 Å for the “acceptor” molecules (molecule B, Fig. 7). The two dimers are rotated about 19.7° relative to each other and form an alternating stack extending along the [1 0 0] direction.

### Mechanistic studies

**Fig. 8:**
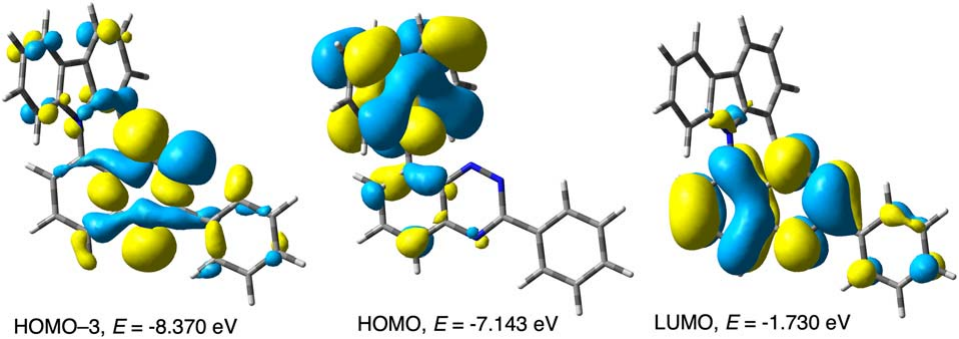
Selected CAM-B3LYP/6-311G(d,p)-derived MO contours and energies relevant to low energy excitations of 2c in EtOAc medium (MO Isovalue = 0.02).

**Fig. 9:**
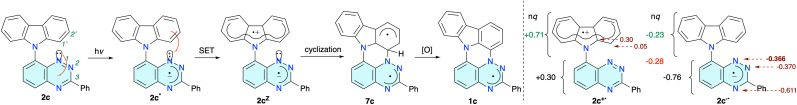
Left: photoexcitation of 2c to the (*n*,π*) state (2c*) and intramolecular single electron transfer (SET) leading to charge separated zwitterionic species 2c^Z^. Partial numbering system is shown for 2c. Right: natural charge (n*q* in *e* units; NBO population analysis) on selected atoms and fragments in radical ions at the S_0_ equilibrium geometry of 2c. UCAM-B3LYP/6-311+G(d,p)//CAM-B3LYP/6-311G(d,p) method in EtOAc dielectric medium. For C(1′) the sum C and H charges is shown.

Photocyclization of precursors 2 can be considered as mechanistically similar to the recently reported cyclization involving a photo-Smiles rearrangement.^29^ Thus, DFT calculations for the carbazole precursor 2c demonstrated that the lowest energy vertical excitation, S_0_ → S_1_, is localized on the benzo[*e*][1,2,4]triazine and has ^1^(*n*,π*) character (2c*). Vibrational relaxation of the Frank–Condon geometry leads to the equilibrium geometry S_1_ state, which also has the ^1^(*n*,π*) character with a formal hole on the MO encompassing all three N lone pairs (*n*, HOMO-3, Fig. 8) and an electron delocalized in the π system of the triazine ring (π*, LUMO, Fig. 8), as shown in Fig. 9.

**Fig. 10:**
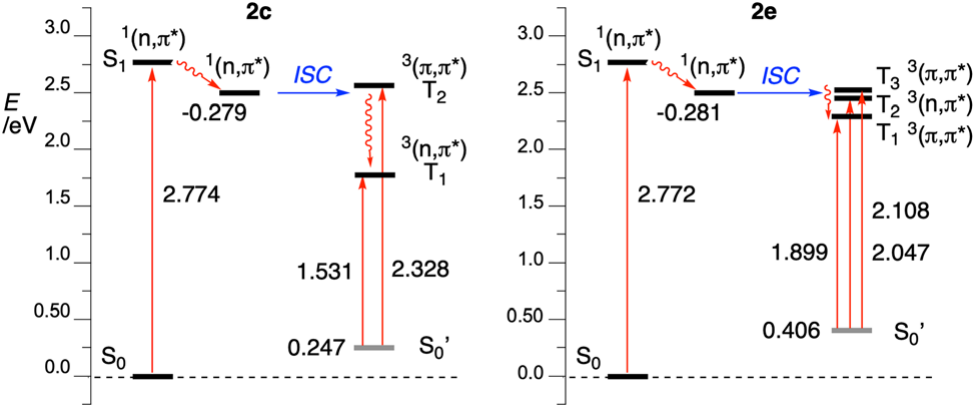
Partial Jablonski diagram for 2c (left) and 2e (right).

The relaxed S_1_ state may undergo intersystem crossing (ISC) to the triplet electron manifold through the energetically accessible (π,π*) T_2_ state (Δ*E*_T_2_–S_1__ = 80 meV, Fig. 10), which is allowed according to the El Sayed rule.^30^ Internal conversion (IC) leads to the relaxed T_1_ state with ^3^(*n*,π*) character and which is also localized on the benzo[*e*][1,2,4]triazine unit. Subsequent intramolecular single electron transfer (SET) from the HOMO localized on the carbazole fragment (Fig. 8) to the *n* orbital leads to the formation of the radical anion-radical cation system 2c^z^, in which the N(1) atoms becomes nucleophilic, while the carbazole electrophilic (Fig. 9). NBO population analysis of radical ions generated by addition (for radical anion 2c^–^˙) or subtraction (for radical cation 2c^+^˙) of an electron to 2c at the S_0_ equilibrium geometry indicates that the natural charge, n*q*, for the N(1) atom is −0.366*e*, while for the combined C(1′)H position is 0.053*e* (Fig. 9). The subsequent polar cyclization leads to diradical 7c, which upon oxidation with molecular oxygen present in the solution gives the observed radical 1c.

The key to successful cyclization in series 2 is the presence of a relatively long-lived ^3^(*n*,π*) T_1_ localized on the benzo[*e*][1,2,4]triazine fragment, and the HOMO localized on the C(8) substituent (carbazole in the case of 2c). This requires an efficient and fast ISC, which can take place between the ^1^(*n*,π*) S_1_ and ^3^(π,π*) T_2_, states of sufficiently close energy. These conditions are satisfied for 2c and its di-*t*-Bu derivative 2d, while analysis of other precursors 2 revealed issues preventing an electronic state favorable for cyclization. Thus, in dibenzocarbazole derivative 2e the ISC can take place between the ^1^(*n*,π*) S_1_ and ^3^(π,π*) T_3_ states (Δ*E*_T_3_–S_1__ = 23 meV, Table 1), but the T_1_ state has the (π,π*) character delocalized on both molecular fragments (Fig. 10). Interestingly, preliminary results for benzo[*e*][1,2,4]triazine with a significantly π-expanded carbazole substituent demonstrate the presence of the desired localized ^3^(*n*,π*) T_1_ state and photocyclization to the expected radical.^31^ The T_1_ state in phenoxazine derivative 2f is again ^3^(π,π*) state, although with a CT character. More importantly, both the S_1_ and T_2_ states have the (π,π*) character, disallowing the ISC, while the S_1_ state is presumably too short-lived for efficient cyclization.^32^ Finally, the indole and benzimidazole derivatives 2a and 2b have the T_1_ state of mixed character delocalized on both molecular fragments, which does not lead to stable charge separation prerequisite for polar cyclization. Energies for Jablonski diagrams for all six derivatives are shown in Table 1, while all diagrams are provided in the ESI.†

**Table 1:** Energy parameters for Jablonski diagram for 2^a^

2	S_0_ → S_1_/eV	Δ*E*_S_1_(rel)–S_0__/eV	Δ*E*_T_2_–S_1_(rel)_/eV	Δ*E*_T_2_–S_0__/eV	Δ*E*_T_1_–S_0__/eV
a	2.779	2.501	0.065	2.566	1.781
b	2.777	2.499	0.109	2.608	1.781
c	2.774	2.495	0.080	2.574	1.777
d	2.775	2.496	0.050	2.546	1.779
e	2.772	2.491	0.023^b^	2.453^c^	2.304
f	2.553	2.488	0.082	2.256	2.176

a CAM-B3LYP/6-311G(d,p) method in EtOAc dielectric medium.

b Energy difference between the S_1(rel)_ and T_3_ state, Δ*E*_T_3_–S_1_(rel)_.

c Energy difference between the S_0_ and T_3_ state, Δ*E*_T_3_–S_0__.

### Electronic absorption spectroscopy

**Fig. 11:**
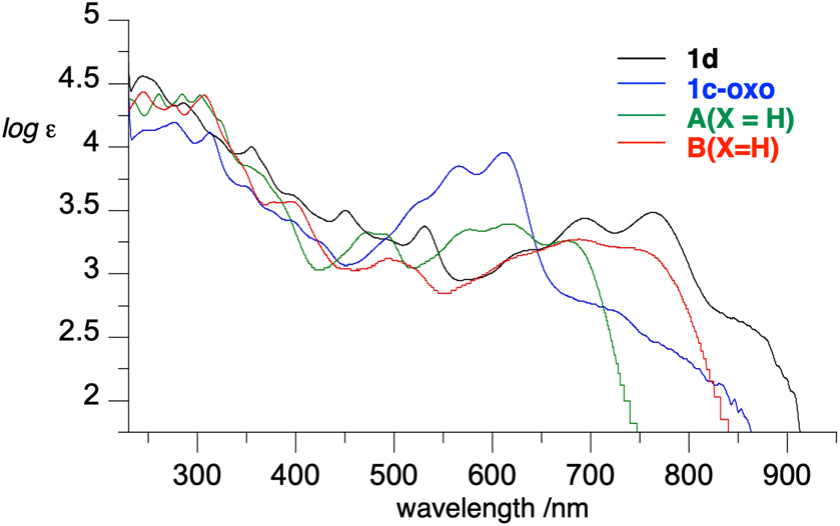
Electronic absorption spectra of 1d, 1c-oxo, A(X=H) and B(X=H) in CH_2_Cl_2_.

To assess the effect of *peri*-annulation of the Blatter radical with indole on electronic properties, radicals 1c and 1d were analyzed with spectroscopic (UV-vis and EPR) and electrochemical methods. Data revealed that in CH_2_Cl_2_ solutions the radicals exhibit the typical strong absorption in the UV region and a group of six overlapping, moderate intensity bands in the visible range extending to about 930 nm (Fig. 11). This indicates that the carbazole-fused radicals 1 have significantly lower optical band gaps (*E*_g_ = 1.30 eV) than parent planar Blatter radicals A(X=H) and B(X=H), *E*_g_ = 1.55 and 1.37 eV, respectively. This result is in agreement with expectations for N-*peri*-annulated Blatter radicals.^18^

TD-DFT calculations revealed that the lowest energy excitation in 1c is solely due to β-HOMO (delocalized on the entire heterocycle) to β-LUMO (localized on the phenazine-triazine system) excitation, which is typical for other Blatter radicals.

Zwitterion 1c-oxo exhibits a strong absorption in the UV region and broad absorption in the visible range with two main absorption maxima at 566 nm (log *ε* = 3.85) and 611 nm (log *ε* = 3.96), and a shoulder absorption at about 710 nm (Fig. 11).

### Electrochemistry

**Fig. 12:**
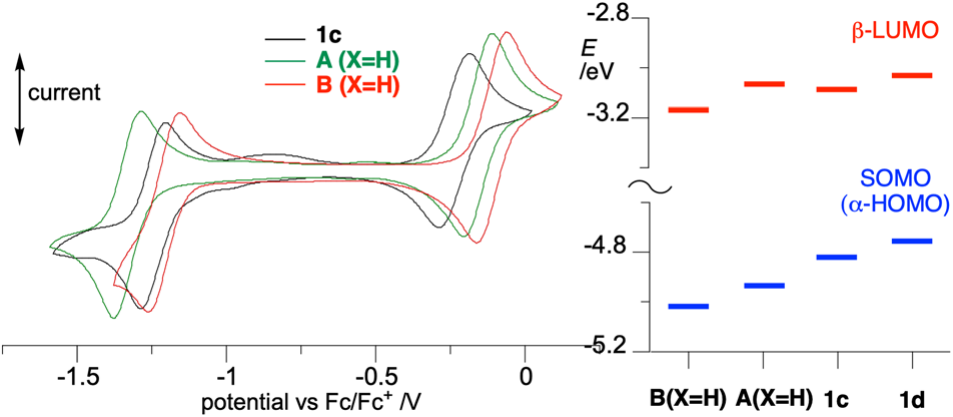
Left: cyclic voltammogram for 1c, A (X=H) and B (X=H) and half-wave potentials referenced to the Fc/Fc^+^ couple (IUPAC convention); 0.5 mM in CH_2_Cl_2_ [*n*-Bu_4_N]^+^ [PF_6_]^–^ (50 mM), at *ca.* 20 °C, 50 mV s^−1^, scans from 0 V in the anodic direction; glassy carbon working electrode, Pt counter electrode, and Ag/AgCl pseudoreference electrode. Right: FMOs energies for selected radicals calculated at the UB3LYP/6-311++G(d,p)//UB3LYP/6-311G(d,p) level of theory in CH_2_Cl_2_ dielectric medium.

**Fig. 13:**
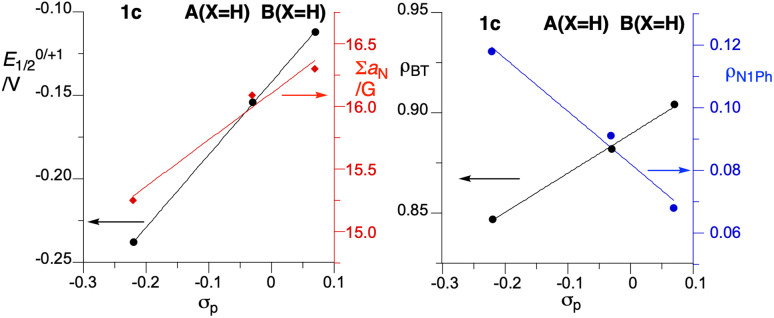
Left: correlation of *E*_1/2_^0/+1^ (black) and the sum of *hfcc a*_N_ for the triazine ring (red) with Hammett parameters σ_p_ for –SPh (0.07), –OPh, (−0.03) and –NPh_2_, (−0.22). Right: correlation of DFT spin density for the benzo[*e*][1,2,4]triazine ring (ρ_BT_, black) and for the benzene ring at the N(1) position of the triazine (ρ_N_1_Ph_, blue) with σ_p_.

Cyclic voltammetry measurements demonstrated quasi-reversible redox processes for radical 1c, as shown in Fig. 12. Data collected in Table 2 demonstrate that *peri*-annulation of the Blatter radical with indole, shifts cathodically the oxidation potential and anodically the reduction potential by about 0.08 V relative to A(X=H) (Fig. 1), consequently narrowing the electrochemical window to 1.005 V. This suggests that *peri*-nitrogen atom affects both FMOs. Closer analysis of the data for the three prototypical radicals, B(X=H), A(X=H) and 1c indicates that the trend in *E*_1/2_^0/+1^ correlates well with Hammett parameters^33^ σ_p_ for model substituents –SPh (0.07), –OPh (−0.03), and –NPh_2_ (−0.22), as shown in Fig. 13.

**Table 2:** Electrochemical,^a^ EPR and DFT data for planar Blatter radicals

Radical	*E*_1/2_^−1/0^/V	*E*_1/2_^0/+1^/V	*E*_cell_/V	*a*_N_1__ b/G	RDV^−1^^c^
A(X=H)^d^	−1.317	−0.154	1.163	7.27	3.843
B(X=H)^d^	−1.202	−0.112	1.090	7.53	3.591
1c	−1.243	−0.238	1.005	7.08	3.988
1d	−1.404^e^	−0.320	1.085	7.14	4.173

a Potential *vs.* the Fc/Fc^+^ couple. See Fig. 12 for details.

b The *hfcc* value ascribed to the N(1) position of the triazine ring.

c Inverse for radical delocalization value. See ESI for details.

d Ref. 16a.

e Partially reversible 2e^−^ process.

Substitution of the carbazole fragment in 1c with *t*-Bu groups shifts the oxidation potential cathodically in 1d, which reflects the electron donating character of the substituents. The reduction of 1d appears to be only a partially reversible 2e^−^ process. Analysis of the data in Table 2 for all four compounds demonstrates good correlation of the oxidation potentials *E*_1/2_^0/+1^ with the calculated level of the α-HOMO (SOMO), and the reduction potential *E*_1/2_^−1/0^ with the energy of the β-LUMO (Fig. 12).

### EPR spectroscopy

**Fig. 14:**
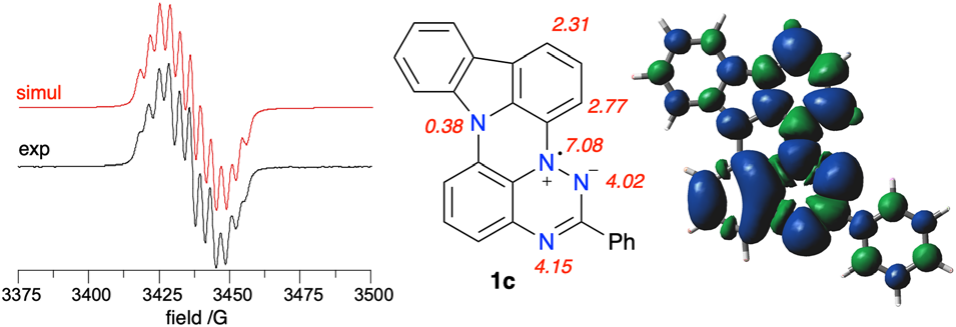
Left: Experimental (black) and simulated (red) EPR spectra for 1c recorded in benzene at *ca.* 20 °C. Right: assignment of the resulting *hfcc* (G) and the total spin density in 1c based on DFT calculations in benzene dielectric medium. Contour values are plotted at ±0.02 (e/bohr^3^)^1/2^.

EPR spectroscopy revealed that the experimental hyperfine coupling constants (*hfcc*) *a*_N_ for 1c (Fig. 14) and 1d are smaller than those for the planar radicals A(X=H) and B(X=H).^16*a*^ Analysis of the data indicates that the sum of three principal *a*_N_ values of the triazinyl ring systematically decreases in the series B(X=H), A(X=H) and 1c, and the trend correlates well with Hammett parameters^33^ σ_p_ for substituent –SPh, –OPh and –NPh_2_, as shown in Fig. 13. The observed change in *a*_N_ values suggests increasing spin delocalization in the series, which is supported with the DFT-calculated Radical Delocalization Value^34^ parameter, RDV^−1^ (Table 2). Thus, with the increasing electron donating ability of the *peri*-annulating fragment (S, O, indole, *t*-Bu-indole) the RDV^−1^ systematically increases (indicating increasing spin delocalization) from 3.591 for B(X=H) to 4.173 for 1d.

Further analysis of DFT data demonstrates that as the electron donating ability of the *peri*-annulating fragment and, consequently, electron density on the benzene ring connected to the triazine N(1) position increases, the spin density shifts from the benzo[*e*][1,2,4]triazine fragment to that benzene ring (Fig. 13). This is presumably due to the increasingly effective stabilization of the zwitterionic resonance form in the triazine, as shown in Fig. 14. As a consequence, the N(1) benzene ring has nearly twice larger spin concentration in 1c than in B(X=H) reaching almost 12% or 15.3% in the entire carbazole unit, as evident from the spin density map in Fig. 14. This spin injection to the carbazole unit and hence effective mixing of electronic states in 1c is much larger than that in 3-(*t*-butylnitroxyl)carbazole (6.6%) or in the recently described *N*-(Ph_2_C˙)carbazole^21*b*^ (6.1%), but short of that in the unstable carbazole-*N*-oxyl radical^35^ (50%).^32^

DFT calculations indicate that the highest spin density on the CH fragment in 1c is in the C(1) and C(3) positions, which is consistent with oxidation and formation of the iminoquinone derivative 1c-oxo (*vide supra*).

### Magnetic properties

**Fig. 15:**
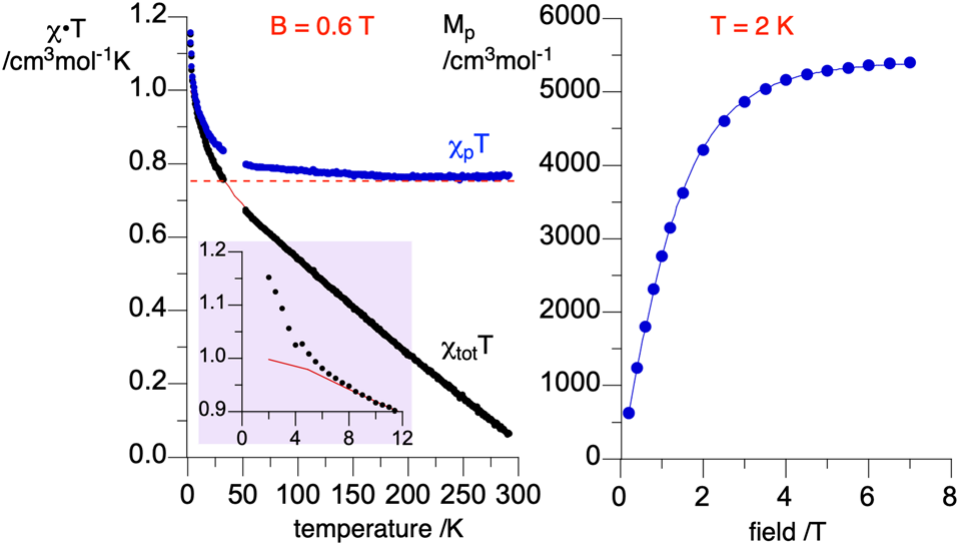
Left: *χ*_tot_*T vs.* temperature (black) data for a discrete dimer of 1d fitted to the Bleaney–Bowers model containing the diamagnetic correction (eqn (1), red line). Fitting parameters: 2*J*/*k*_B_ = 16.6(2) K and *χ*_dia_ = −0.002418(3) cm^3^ mol^−1^; *r*^2^ = 0.998. Data in the range 30–50 K contaminated with traces of O_2_ are removed from analysis. The purple inset shows the low temperature portion of the plot. Using the *χ*_dia_ value, *χ*_p_ was obtained and shown as the *χ*_p_*T vs.* temperature (blue) plot. The horizontal dotted line marks *χ*_p_*T* = 0.750 cm^3^ mol^−1^ K for two ideal spins 1/2. Right: molar paramagnetic magnetization, *M*p, *vs.* field at 2 K fitted to the Brillouin function with *S* = 1.56(1), *r*^2^ = 0.999.

The magnetic susceptibility of radical 1d was measured as a function of temperature in the range of 290 → 2 K at 0.60 T. Results shown in Fig. 15 indicate ferromagnetic interactions at low, and ideal paramagnetic behaviour at high temperatures. Detailed analysis of the data was conducted for a two-spin pair of radicals 1d as the fundamental structural unit in the solid-state structure (*vide supra*, Fig. 5). Thus, fitting the *χ*_tot_*T*(*T*) datapoints to the Bleaney–Bowers model^36^ (BB model for two spins *S* = 1/2 based on Ĥ = −2*J*Ŝ_1_˙Ŝ_2_ Hamiltonian) containing the diamagnetic correction term, *χ*_dia_ (eqn (1)), gave the ferromagnetic exchange interaction 2*J*/*k*_B_ = 16.6(1) K or Δ*E*_S–T_ = 33(2) cal mol^−1^. The model describes the experimental data well down to 8 K, while below this temperature the χ_tot_*T* values are larger than expected (see inset in Fig. 15). This increase in magnetization suggests additional ferromagnetic-like spin–spin interactions, which could be related to the rotational disorder of the *t*-Bu groups observed in the solid-state structure at higher temperatures. The observed behavior of the χ_tot_*T*(*T*) is consistent with the *M*(H) measurements at 2K (Fig. 15), which are well described with the Brillouin function (eqn (2)) for *S* = 1.56(1). This indicates that, on average, three spins *S* = 1/2 interact ferromagnetically at 2 K in the solid-state sample of 1d.1
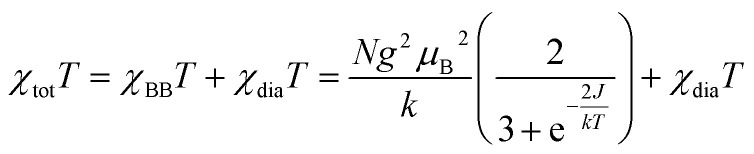
2
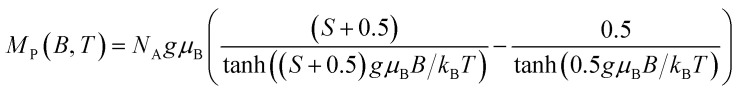


An attempt to account for these additional ferromagnetic interactions by using the BB model with the mean field approximation^37^ gave a similar primary exchange interaction, 2*J*/*k*_B_ = 12.1(4) K, and negligible small average mean field interactions (2*J*/*k*_B_ = 0.10(1) K) without proper fitting of the low temperature data.^32^

For comparison purposes, the exchange interaction in 1d was calculated using the usual broken symmetry (BS) approach and the Yamaguchi formalism^38^ (eqn (3)), where *E* is the SCF energy corrected for ZPE and <*S*^2^> is the total spin angular momentum of high (*T*) or low (OSS) spin state. Thus, single-point DFT calculations at the UB3LYP/6-311+G(d) level of theory for the discrete dimer of 1d at its crystallographic coordinates gave the Δ*E*_S–T_ (DFT) = 0.15 kcal mol^−1^. These experimental and computational results are consistent with the types of intermolecular close contacts between the radicals in the discrete dimer (*vide supra*).3
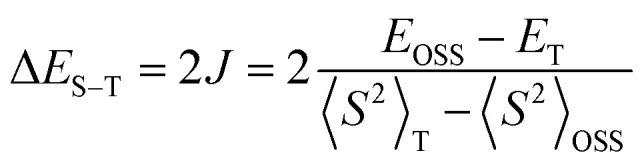


## Conclusions

Photocyclization of two carbazole derivatives of benzo[*e*][1,2,4]triazine, 2c and 2d, led to persistent radicals, 1c and 1d, respectively, which are the first examples of a novel heterocyclic system. The presence of the *peri*-annulating N atom with the available lone pair has a significant impact on the electronic properties of the Blatter radical: it lowers the optical band gap to 1.30 eV, cathodically shifts oxidation potentials, and increases spin delocalization onto the benzene ring at the N(1) position of the triazine, which confirm the original hypothesis. The last two effects correlate well with Hammett parameters for model substituents, –SPh, –OPh, and –NPh_2_. The increased spin delocalization onto the N(1)–Ph is supported by the observed oxidation of 1c at its highest spin density site and formation of 1c-oxo. On the other hand, this delocalization is important for magnetic behavior of 1d in the solid state and observed ferromagnetic interactions. Overall, fused Blatter is much more effective spin injector to carbazole than nitroxide or diphenylmethyl.

Detailed DFT analysis of carbazole precursors, that gave the cyclization products, and analogous derivatives which were not photoreactive, indicate that the successful cyclization requires efficient access to the long-lived ^3^(*n*,π*) T_1_ state localized on the benzo[*e*][1,2,4]triazine, and a higher energy HOMO localized on the C(8) substituent (*e.g*. carbazole). This allows for an intramolecular SET process and subsequent cyclization of the resulting charge-separated radical ion fragments. These features are found in the two carbazole precursors, 2c and 2d, while in the remaining four derivatives 2 this localized ^3^(*n*,π*) T_1_ state is either not present or not attainable. The proposed mechanism is supported by the observed solvent effect on yields of the radicals: it is higher in AcOEt than in CH_2_Cl_2_ as the reaction medium as expected for a polar mechanism. It also provides a better understanding of the design features of derivatives 2 for successful cyclization and formation of radicals 1. For instance, a derivative 2 containing a chiral helical carbazole substituent satisfying the mechanistic requirements and the corresponding radical was just prepared. These results will be reported elsewhere.

The two radicals 1c and 1d constitute a new class of stable radicals of the general structure D, which complements series A and B and expands the range of tunability of electronic and magnetic properties for applications in the broad area of molecular electronics.

## Author contributions

The manuscript was written through contributions of all authors, and all authors have given approval to the final version of the manuscript. Conceptualization: P. B., P. K.; investigation and writing: all authors; supervision and data curation: P. B, P. K.; funding acquisition: P. K.

## Conflicts of interest

The authors declare no competing financial interest.

## Supplementary Material

SC-016-D5SC01182E-s001

SC-016-D5SC01182E-s002

## Data Availability

Data for this article, including synthetic procedures and characterization details (NMR, EPR, UV-vis, E-chem), crystallographic and magnetic data, and computational results, are available at DOI: https://doi.org/10.1039/D5SC01182E. The data supporting this article have been included as part of the ESI.† Crystallographic data for 1c-oxo and 1d have been deposited at the Cambridge Crystallographic Data Centre (CCDC) under CCDC numbers: 2327428 and 2327427, respectively.
