# Supplementary material for: Photochemical synthesis of carbazole-fused Blatter radicals: effective spin injection to the carbazole system
Source: Chem Sci. 2025 May 27;16(26):12139–47. doi: 10.1039/d5sc01182e (PMC12143299; doi:10.1039/d5sc01182e)
Supplement: SC-016-D5SC01182E-s001 [file SC-016-D5SC01182E-s001.pdf]

Supplementary Information (SI) for Chemical Science.
This journal is © The Royal Society of Chemistry 2025

Electronic Supplementary Information

for

Photochemical synthesis of carbazole-fused Blatter radicals: Effective spin injection to the carbazole system

Paulina Bartos,[a*] Patrycja Szamweber,[b] Bruno Camargo,[c] Anna Pietrzak,[d] Piotr Kaszyński[a,b,e*]

[a] Faculty of Chemistry, University of Łódź, 91-403 Łódź, Poland
[b] Centre of Molecular and Macromolecular Studies, Polish Academy of Sciences, 90-363 Łódź, Poland
[c] Institute of Experimental Physics, Faculty of Physics, University of Warsaw, 02-093 Warsaw, Poland
[d] Faculty of Chemistry, Łódź University of Technology, 90-924 Łódź, Poland
[e] Department of Chemistry, Middle Tennessee State University, Murfreesboro, TN, 37132

Table of Content:

1. Synthetic details S2
2. Nomenclature of radical **1** according to IUPAC rules …..S7
3. NMR spectra S9
4. XRD data collection and refinement for **1d** and **1c-*oxo*** S17
5. Electronic absorption spectroscopy S21
6. Stability of radicals **1c** and **1d** towards atmospheric oxygen S26
7. Electrochemical results S27
8. EPR Spectroscopy S29
9. SQUID magnetometry S31
10. Computational details and results S37
   a) *mechanistic investigation of photocyclization of **2*** S37
   b) *isotropic Fermi contact coupling constants (hfcc) and spin densities for radicals* S38
   c) *spin delocalization in radicals in benzene dielectric medium* S42
   d) *electronic excitations* S43
   e) *partial output data from TD-DFT calculations for radicals **1*** S45
   f) *intermolecular interaction energy calculations* S58
11. Archive for DFT geometry optimization results. S58
12. References S75

1. Synthetic details

General. Reagents and solvents were obtained commercially. For separation of radicals, silica gel 60 (35-70 micron) passivated with Et_3N or high-purity grade silica gel (w/ Ca, ~0.1%; purchased from Sigma-Aldrich, product no. 60752-1KG) were used. NMR spectra were obtained at 600 and 400 MHz (^1H), and 151 and 101 MHz (^{13}C) in $CDCl_3$ and referenced to the solvent (δ = 7.26 ppm for ^1H and δ = 77.16 ppm for ^{13}C).[1] IR spectra were recorded using Nexus FT-IR Thermo Nilolet IR spectrometer in KBr tablets or an Agilent Cary 630 FTIR spectrometer, in neat. UV spectra were measured in CH_2Cl_2 on PerkinElmer Lambda 45 spectrophotometer. Melting points were determined on a Stuart SMP30 Advanced Digital Melting Point Apparatus and are uncorrected. High-resolution mass spectrometry (HRMS) measurements were performed using SYNAPT G2-Si High Resolution Mass Spectrometry equipped with an ESI or APCI source and Quantitative Time-of-Flight (QuanTof) mass analyzer. Irradiations were conducted with a 300 W halogen lamp ("Portable halogen Work Lamp" without the protecting front glass window) equipped with a T3 double-ended RSC base J118 light bulb.

Photochemical preparation of radicals 1. General procedure.
A solution of precursor **2** (0.10 mmol, see the ESI for synthesis) in CH_2Cl_2, EtOAc or EtOH (100 mL) was placed in a 250 mL borosilicate round bottom flask fitted with a reflux condenser. The solution was stirred and irradiated with a 300 W halogen lamp, which was set about 30 cm from the flask. The reaction mixture was allowed to warm up to 30–35°C. Progress of the reaction was monitored by TLC (20% EtOAc/petroleum ether) and the irradiation was stopped after 72 h. The solvent was evaporated, the residue was adsorbed onto silica gel (w/Ca content, ~0.1%) and the products were separated from unreacted starting **2** (if present) using a short chromatographic column (silica gel with "Ca", ~0.1%) using EtOAc in pet. ether gradient 5–10%. The obtained radical was recrystallized.

6-Phenyl-7H-indolo[3,2,1-de][1,2,4]triazino[5,6,1-kl]phenazin-7-yl ***(1c)****.* Obtained from **2c** (37.2 mg, 0.1 mmol) in CH_2Cl_2: 14.1 mg (37% yield); in EtOAc: 18.4 mg (45% yield); in EtOH; 3.4 mg (9% yield); black brown microcrystals: mp 264–266 °C (hexane/ EtOAc 4:1); IR (KBr) ν 1587, 1483, 1449, 1394, 1353, 1340, 1322, 771, 736, 688 cm^{-1}; UV (CH_2Cl_2) λ_{max} (log ε) 852.0 sh (2.63), 763.0 (3.48), 693.5 (3.44), 636.0 (3.19), 531.0 (3.31), 450.0 (3.50), 355.0 (4.00), 285.5 (4.35), 244.0 (4.56) nm; HRMS

(ESI-TOF) $[M+H]^+$ *m/z* calcd for $C_{25}H_{16}N_3$: 372.1375; found: 372.1360. Anal. Calcd for $C_{25}H_{15}N_4$: C, 80.84; H, 4.07; N, 15.08. Found: C, 80.82; H, 4.08; N, 15.14%.

*1-Oxo-6-phenyl-1H-indolo[3,2,1-de][1,2,4]triazino[5,6,1-kl]phenazin-4-ium-5-ide (**1c-oxo**).* Obtained as a highly polar byproduct (AcOEt eluent) in photocyclization of **2c** (37.2 mg, 0.1 mmol) in CH_2Cl_2: 5.6 mg (12% yield); in EtOAc: 5.0 mg (13% yield); dark blue micro-crystals: mp 268–270 °C; ^1H NMR (400 MHz, $CDCl_3$) δ 8.54 (d, J = 8.1 Hz, 1H), 8.38–8.35 (m, 2H), 8.24 (d, J = 9.9 Hz, 1H), 8.16 (d, J = 6.8 Hz, 1H), 7.91–7.83 (m, 2H), 7.59–7.52 (m, 5H), 7.44 (d, J = 7.7 Hz, 1H), 6.82 (d, J = 10.1 Hz, 1H); $^{13}C\{^1H\}$ NMR (101 MHz, $CDCl_3$) δ 179.7, 161.5, 148.3, 134.9, 134.7, 133.1, 132.2, 131.8, 130.4, 128.7, 128.4, 128.0, 126.0, 125.5, 123.2, 120.7, 119.9, 119.1, 113.9, 111.2, 108.7; IR (neat) ν 1682, 1571, 1536, 1506, 1491, 1435, 1380, 1330, 1179, 1125, 1043, 971, 781, 746, 693 cm^{-1}; UV (CH_2Cl_2) λ_{max} (log ε) 702.0 (2.76), 612.0 (3.96), 566.0 (3.85), 526.5 (3.57), 312.5 (4.12), 276.0 (4.20) nm; HRMS (AP-TOF) *m/z* $[M+H]^+$ Calcd for $C_{25}H_{15}N_4O$: 387.1246; found: 387.1253. Anal. Calcd for $C_{25}H_{14}N_4O$: C, 77.71; H, 3.65; N, 14.50; O, 4.14. Found: C, 77.42; H, 3.78; N, 14.61%.

*2,14-Di-tert-butyl-6-phenyl-7H-indolo[3,2,1-de][1,2,4]triazino[5,6,1-kl]phenazin-7-yl (**1d**).* Obtained from **2d** (48.5 mg, 0.1 mmol) in CH_2Cl_2: 1.1 mg (~2% yield); in EtOAc: 33.9 mg (70% yield); in EtOH: 0.5 mg (~1% yield); black brown microcrystals: mp 294.0–295.0 °C (MeCN/CH_2Cl_2); IR (neat) ν 2954, 1578, 1487, 1444, 1389, 1264, 1140, 851, 770, 691 cm^{-1}; UV (CH_2Cl_2) λ_{max} (log ε) 852.0 sh (2.62), 781.0 (3.78), 708.0 (3.69), 650.0 sh (3.40), 535.5 (3.45), 456.5 (3.69), 357.5 (4.16), 288.5 (4.52), 245.0 (4.72) nm; HRMS (ESI-TOF) $[M+H]^+$ *m/z* calcd for $C_{33}H_{32}N_4$: 484.2627; found: 484.2604. Anal. Calcd for $C_{33}H_{31}N_4$: C, 81.95; H, 6.46; N, 11.58. Found: C, 81.65; H, 6.87; N, 11.55%.

Preparation of 8-substituted 3-phenylbenzo[*e*][1,2,4]triazines 2. General procedure.

A mixture of 8-fluoro-3-phenylbenzo[*e*][1,2,4]triazine[2] (**3**, 225.0 mg, 1.0 mmol), appropriate heterocycle **4** (1.1 mmol), and 60% NaH (45.0 mg, 1.1 mmol) in dry DMSO (6 mL) were stirred at 80 °C for 3 h under Ar. After cooling, CH_2Cl_2 (30 mL) was added, and the organic layer was washed well with H_2O (3 × 25 mL) and brine (25 mL). The organic layer was dried (Na_2SO_4), and the solvent was removed in *vacuo*. The resulting solid residue was absorbed

onto silica and purified by column chromatography using EtOAc in pet. ether gradient 5–10%. The solvent was evaporated, and the product was recrystallized.

8-(1*H*-Indol-1-yl)-3-phenylbenzo[*e*][1,2,4]triazine (2a).

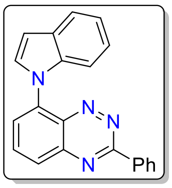

Obtained 280.0 mg (87% yield) as yellow crystals: mp 198–199 °C (EtOAc); ^{1}H NMR (600 MHz, $CDCl_3$) δ 8.82-8.77 (m, 2H), 8.12 (dd, J_1 = 8.6 Hz, J_2 = 1.5 Hz, 1H), 8.09 (t, J = 7.8 Hz, 1H), 7.99 (d, J = 7.1 Hz, 1H), 7.78 (d, J = 3.0 Hz, 1H), 7.77–7.74 (m, 1H), 7.64–7.58 (m, 3H), 7.50–7.46 (m, 1H), 7.26–7.21 (m, 2H), 6.85 (d, J = 3.2 Hz, 1H), ^{13}C{^{1}H} NMR (151MHz, $CDCl_3$) δ 160.0, 142.2, 142.1, 137.7, 137.2, 135.5, 135.3, 132.0, 131.2, 129.8, 129.2, 129.1, 127.7, 125.7, 122.8, 121.4, 121.2, 110.6, 104.9; IR (KBr) v 1601, 1563, 1509, 1457, 1389, 1355, 1318, 1244, 1211, 1149, 1010, 799, 745, 703 cm^{-1}; UV (CH_2Cl_2) λ_{max} (log ε) 427.5 (3.40), 352.0 (3.62), 306.0 (4.13), 261.5 (4.63) nm; HRMS (ESI-TOF) $[M+H]^+$ calcd for $C_{21}H_{15}N_4$: 323.1297; found: 323.1299. Anal. Calcd for $C_{21}H_{14}N_4$: C, 78.24; H, 4.38; N, 17.38. Found: C, 78.25; H, 4.34; N, 17.37%.

8-(1*H*-Benzo[*d*]imidazol-1-yl)-3-phenylbenzo[*e*][1,2,4]triazine (2b).

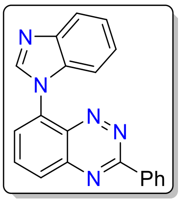

Obtained 301.0 mg (93% yield) as yellow crystals: mp 200–201 °C (EtOAc); ^{1}H NMR (600 MHz, $CDCl_3$) δ 8.81-8.77 (m, 2H), 8.56 (s, 1H), 8.23 (d, J = 8.5 Hz, 1H), 8.16 (t, J = 8.0 Hz, 1H), 8.02 (d, J = 7.5 Hz, 1H), 7.97 (d, J_1 = 8.0 Hz, 1H), 7.64–7.60 (m, 3H), 7.49 (d, J = 7.9 Hz, 1H), 7.40 (t, J = 7.5 Hz, 1H), 7.36 (t, J = 7.5 Hz, 1H); ^{13}C{^{1}H} NMR (151MHz, $CDCl_3$) δ 160.6, 144.8, 144.0, 142.2, 141.4, 135.5, 135.0, 134.6, 134.5, 132.3, 129.31, 129.28, 129.2, 125.3, 124.2, 123.5, 121.0, 110.6; IR (neat) v 1605, 1563, 1502, 1449, 1385, 1327, 1242, 1198, 1010, 803, 764, 737, 705 cm^{-1}; UV (CH_2Cl_2) λ_{max} (log ε) 352.5 (3.78), 295.0 sh (4.29), 260 (4.59) nm; HRMS (ESI-TOF) $[M+H]^+$ calcd for $C_{20}H_{14}N_5$: 324.1249; found: 324.1243. Anal. Calcd for $C_{20}H_{13}N_5$: C, 74.29; H, 4.05; N, 21.66. Found: C, 74.30; H, 4.11; N, 21.45%.

9-(3-Phenylbenzo[*e*][1,2,4]triazin-8-yl)-9*H*-carbazole (2c).

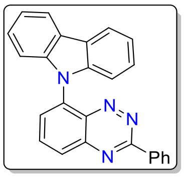
Obtained 276.5–281.0 mg (73–75% yield) as yellow crystals: mp 268.0–269.0 °C (EtOAc); ^{1}H NMR (400 MHz, $CDCl_3$) δ 8.79-8.75 (m, 2H), 8.28 (dd, J_1 = 8.6 Hz, J_2 = 1.0 Hz, 1H), 8.22-8.17 (m, 3H), 8.02 (dd, J_1 = 7.2 Hz, J_2 = 1.1 Hz, 1H), 7.60–7.57 (m, 3H), 7.36 (td, J_1 = 7.5 Hz, J_2 = 1.2 Hz, 2H), 7.32 (td, J_1 = 7.1 Hz, J_2 = 0.7 Hz, 2H), 7.12 (d, J = 8.0 Hz, 2H); ^{13}C{^{1}H} NMR (101 MHz, $CDCl_3$) δ 160.2, 143.0, 142.4, 142.3, 136.6, 135.8, 135.3, 132.0, 129.7, 129.6, 129.2, 129.1, 126.2, 124.1, 120.6, 120.5, 110.2; IR (KBr) v 1606, 1563, 1504, 1451, 1371, 1324, 1276, 1232, 1004, 803, 750, 706 cm^{-1}; UV (CH_2Cl_2) λ_{max} (log ε) 444.5 (3.17), 354.5 (3.65), 333.5 (4.05), 319.5 (4.09), 270.5 (4.63), 258.5 (4.65), 248.5 (4.66), 239.0 (4.78) nm; HRMS (ESI-TOF) $[M+H]^+$ calcd for $C_{25}H_{17}N_4$: 373.1453; found: 373.1458. Anal. Calcd for $C_{25}H_{16}N_4$: C, 80.63; H, 4.33; N, 15.04. Found: C, 80.59; H, 4.27; N, 15.06%.

3,6-Di-*tert*-butyl-9-(3-phenylbenzo[*e*][1,2,4]triazin-8-yl)-9*H*-carbazole (2d).

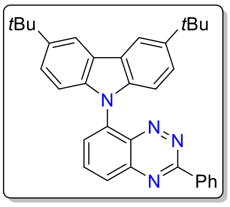
Obtained 448.0 mg (92% yield) as yellow crystals: mp 301–303 °C (EtOAc); ^{1}H NMR (400 MHz, $CDCl_3$) δ 8.78 – 8.76 (m, 2H), 8.23 (dd, J_1 = 8.6, J_2 = 1.3 Hz, 1H), 8.18 – 8.17 (m, 2H), 8.15 (dd, J_1 = 8.6 Hz, J_2 = 7.3 Hz, 1H), 7.99 (dd, J_1 = 7.3, J_2 = 1.3 Hz, 1H), 7.61 – 7.57 (m, 3H), 7.40 (dd, J_1 = 8.6 Hz, J_2 = 1.9 Hz, 2H), 7.05 (d, J = 8.6 Hz, 2H), 1.46 (s, 18H); ^{13}C{^{1}H} NMR (101 MHz, $CDCl_3$) δ 160.1, 143.5, 143.0, 142.4, 140.8, 137.2, 135.8, 135.4, 131.9, 129.2, 129.15, 129.1, 129.0, 124.2, 123.9, 116.5, 109.7, 34.9, 32.2; IR (neat) v 2955, 1609, 1564, 1482, 1360, 1290, 1236, 1176, 1102, 1057, 1009, 882, 797, 699 cm^{-1}; UV (CH_2Cl_2) λ_{max} (log ε) 468.5 (3.23), 363.5 sh (3.59), 339.0 (4.06), 326.5 (4.07), 293.0 (4.50), 272.5 (4.62), 252.0 (4.69), 242.0 (4.77) nm; HRMS (ESI-TOF) $[M+H]^+$ calcd for $C_{33}H_{33}N_4$: 485.2705; found: 485.2705. Anal. Calcd for $C_{33}H_{32}N_4$: C, 81.78; H, 6.66; N, 11.56. Found: C, 81.72; H, 6.73; N, 11.54%.

7-(3-Phenylbenzo[*e*][1,2,4]triazin-8-yl)-7*H*-dibenzo[*c,g*]carbazole (2e).

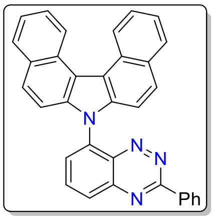

Obtained 417.0 mg (88% yield) as yellow crystals: mp 295.0–297.0 °C (EtOAc); ^{1}H NMR (400 MHz, $CDCl_3$) δ 9.31 (d, J = 8.5 Hz, 2H), 8.76-8.74 (m, 2H), 8.36 (dd, J_1 = 8.6 Hz, J_2 = 1.2 Hz, 1H), 8.23 (dd, J_1 = 8.7 Hz, J_2 = 7.3 Hz, 1H), 8.07 (dd, J_1 = 7.2 Hz, J_2 = 1.2 Hz, 1H), 8.03 (dd, J_1 = 8.2 Hz, J_2 = 1.4 Hz, 2H), 7.80 (d, J = 8.8 Hz, 2H), 7.74 (ddd, J_1 = 8.4 Hz, J_2 = 7.7 Hz, J_3 = 1.4 Hz, 2H), 7.60 - 7.53 (m, 5H), 7.30 (d, J = 8.8 Hz, 2H); ^{13}C{^{1}H} NMR (101 MHz, $CDCl_3$) δ 160.5, 143.3, 142.3, 139.5, 135.8, 135.6, 135.2, 132.1, 130.7, 130.6, 130.56, 129.3, 129.28, 129.2, 129.15, 127.2, 125.71, 125.67, 123.7, 118.6, 111.8; IR (neat) ν 1609, 1562, 1502, 1381, 1322, 1175, 1108, 1059, 1009, 919, 791, 702 cm^{-1}; UV (CH_2Cl_2) λ_{max} (log ε) 429.5 (3.16), 364.5 (4.48), 346.5 (4.37), 303.0 (4.50), 378.0 (4.84) nm; HRMS (ESI-TOF) [M+H]$^+$ Calcd for $C_{33}H_{21}N_4$: 473.1766; found: 473.1772. Anal. Calcd for $C_{33}H_{20}N_4$: C, 83.88; H, 4.27; N, 11.86. Found: C, 83.72; H, 4.48; N, 11.93%.

10-(3-Phenylbenzo[*e*][1,2,4]triazin-8-yl)-10*H*-phenoxazine (2f).

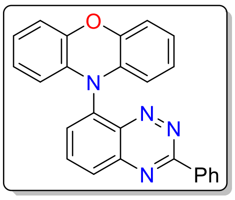

Obtained 341.8 mg (88% yield) as yellow crystals: mp 252.5–253.5 °C (EtOAc); ^{1}H NMR (600 MHz, $CDCl_3$) δ 8.78-8.74 (m, 2H), 8.27 (dd, J_1 = 8.6 Hz, J_2 = 1.0 Hz, 1H), 8.17 (dd, J_1 = 8.6 Hz, J_2 = 7.2 Hz, 1H), 7.91 (dd, J_1 = 7.2 Hz, J_2 = 1.1 Hz, 1H), 7.60–7.57 (m, 3H), 6.76 (dd, J_1 = 8.0 Hz, J_2 = 1.1 Hz, 2H), 6.66 (td, J_1 = 7.9 Hz, J_2 = 1.5 Hz, 2H), 6.49 (td, J_1 = 7.7 Hz, J_2 = 1.3 Hz, 2H), 5.73 (dd, J_1 = 7.9 Hz, J_2 = 1.1 Hz, 2H); ^{13}C{^{1}H} NMR (151 MHz, $CDCl_3$) δ 160.3, 144.1, 143.7, 143.6, 137.6, 136.5, 135.2, 134.7, 134.1, 132.0, 130.9, 129.2, 129.1, 123.3, 122.0, 116.0, 113.4; IR (neat) ν 1589, 1563, 1490, 1384, 1338, 1271, 1080, 1044, 1001, 798, 738, 709 cm^{-1}; UV (CH_2Cl_2) λ_{max} (log ε) 460.0 (2.66), 320.0 sh (4.05), 276.0 (4.45), 260.0 (4.48), 239.5 (4.71) nm; HRMS (ESI-TOF) *m/z* [M+H]$^+$ calcd for $C_{25}H_{17}N_4O$: 389.1402; found: 389.1399. Anal. Calcd for $C_{25}H_{16}N_4O$: C, 77.30; H, 4.15; N, 14.42. Found: C, 77.12; H, 4.49; N, 14.21%.

2-((3-Phenylbenzo[e][1,2,4]triazin-8-yl)amino)benzaldehyde (5).

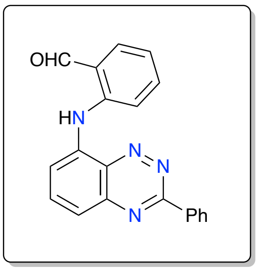

A solution of precursor **2a** (32.6 mg, 0.1 mmol) in EtOH (100 mL) was irradiated for 72 h. The reaction mixture was separated by column chromatography using EtOAc in pet. ether gradient 10-20% giving 17.4 mg (53% yield) of aldehyde **5** as orange crystals: mp 143-145 °C (EtOAc); ^{1}H NMR (600 MHz, $CDCl_3$) δ 11.88 (s, 1H), 10.08 (s, 1H), 8.80-8.77 (m, 2H), 7.88 (d, J = 8.0 Hz, 1H), 7.85 (d, J = 8.5 Hz, 1H), 7.81 (d, J = 7.6 Hz, 1H), 7.77 (dd, J_1 = 7.7 Hz, J_2 = 1.5 Hz, 1H), 7.62-7.57 (m, 5H), 7.13 (t, J = 7.1 Hz, 1H); ^{13}C{^{1}H} NMR (176 MHz, $CDCl_3$) δ 194.1, 160.7, 143.8, 142.5, 139.7, 139.5, 137.0, 136.9, 135.8, 135.4, 131.6, 129.1, 129.0, 123.2, 120.6, 120.0, 115.9, 112.3; IR (neat) v 2922, 2851, 2734, 1665, 1579, 1456, 1371, 1316, 1166, 877, 760, 686 cm^{-1}; HRMS (ESI-TOF) m/z [M+H]$^{+}$ Calcd for $C_{20}H_{15}N_4O$: 327.1240; found: 327.1241.

2. Nomenclature of radicals 1 according to IUPAC rules

In blue: numbering of the main structure. In green: numbering of the [1,2,4]triazinyl fused ring. In red: assigned letters to the phenazine core.

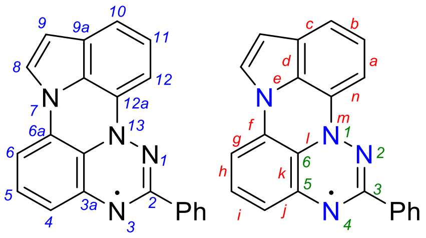

2-Phenyl-3*H*-pyrrolo[3,2,1-*de*][1,2,4]triazino[5,6,1-*kl*]phenazin-3-yl (**1a**).

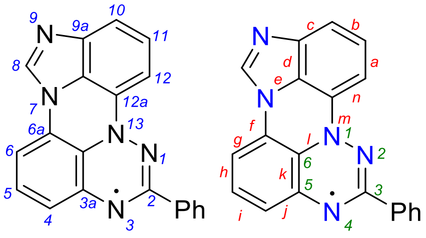

2-Phenyl-3*H*-imidazo[4,5,1-*de*][1,2,4]triazino[5,6,1-*kl*]phenazin-3-yl (**1b**).

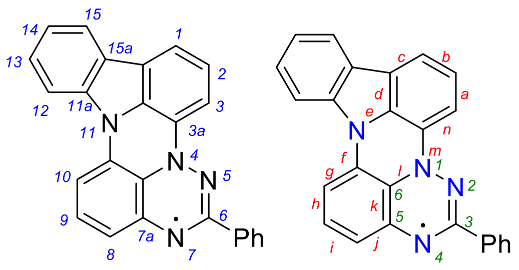

6-Phenyl-7*H*-indolo[3,2,1-*de*][1,2,4]triazino[5,6,1-*kl*]phenazin-7-yl (**1c**).

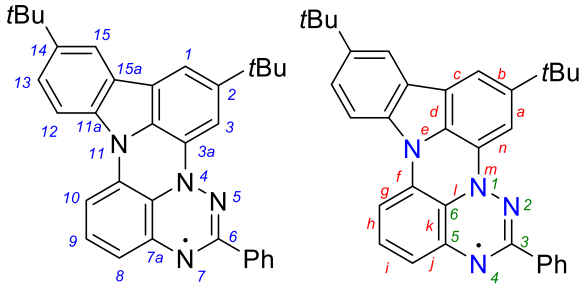

2,14-Di-*tert*-butyl-6-phenyl-7*H*-indolo[3,2,1-*de*][1,2,4]triazino[5,6,1-*kl*]phenazin-7-yl (**1d**).

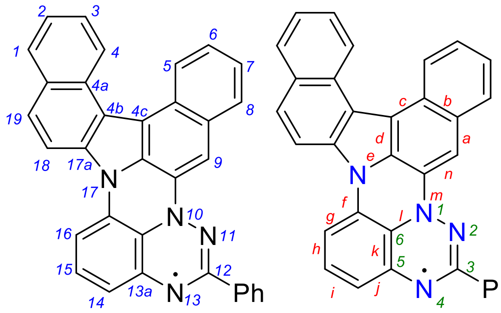

12-Phenyl-13*H*-benzo[*b*]benzo[4,5]indolo[3,2,1-*de*][1,2,4]triazino[5,6,1-*kl*]phenazin-13-yl (**1e**).

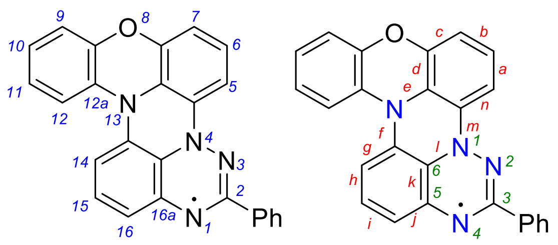

2-Phenyl-1*H*-benzo[5,6][1,4]oxazino[2,3,4-*de*][1,2,4]triazino[5,6,1-*kl*]phenazin-1-yl (**1f**).

3. NMR spectra

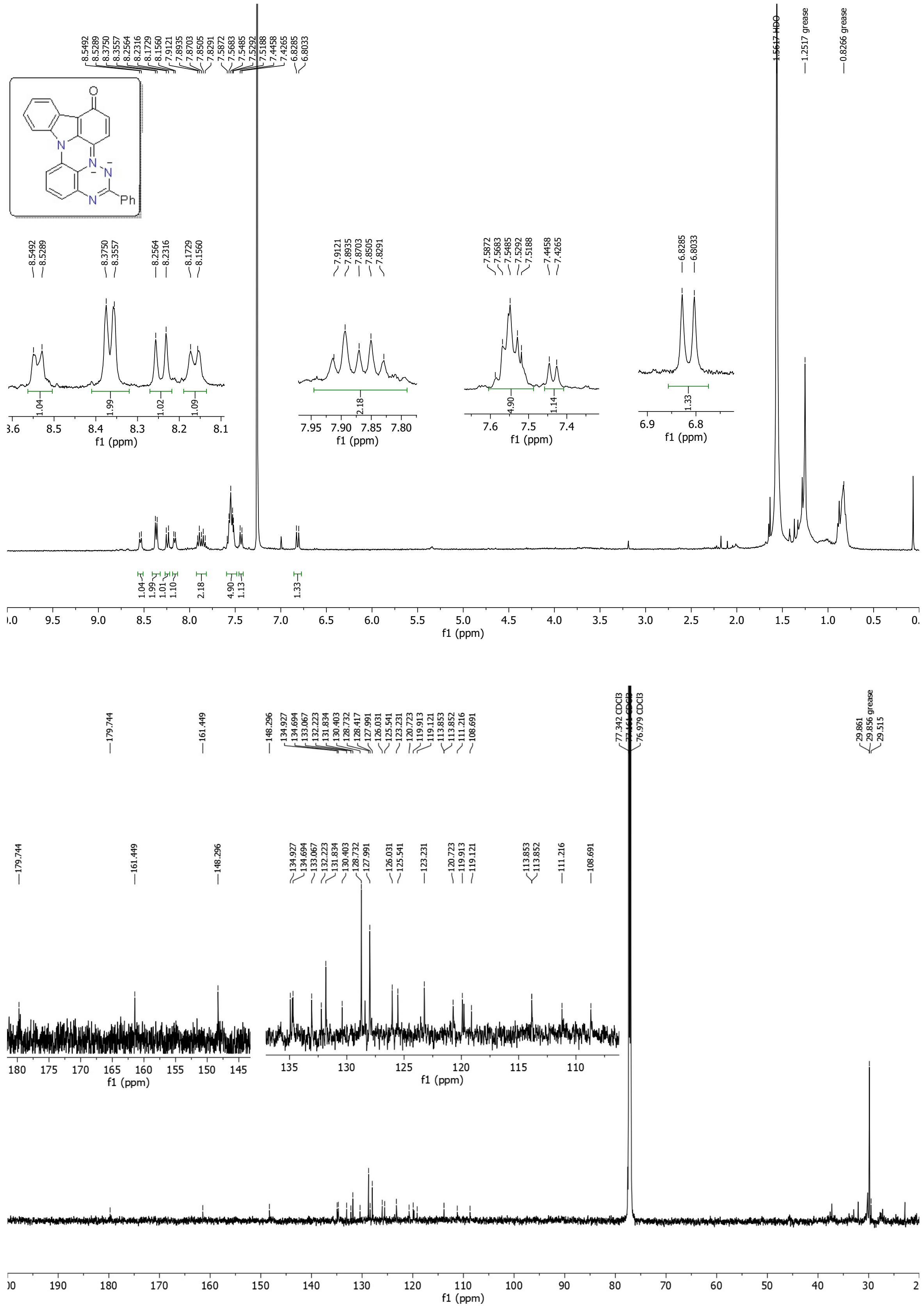

Figure S1. ^{1}H NMR and ^{13}C NMR spectra of 1-oxo-6-phenyl-1*H*-indolo[3,2,1-*de*][1,2,4]triazino[5,6,1-*kl*]phenazin-4-ium-5-ide (**1c-*oxo***) recorded in $CDCl_3$ at 400 and 101 MHz, respectively.

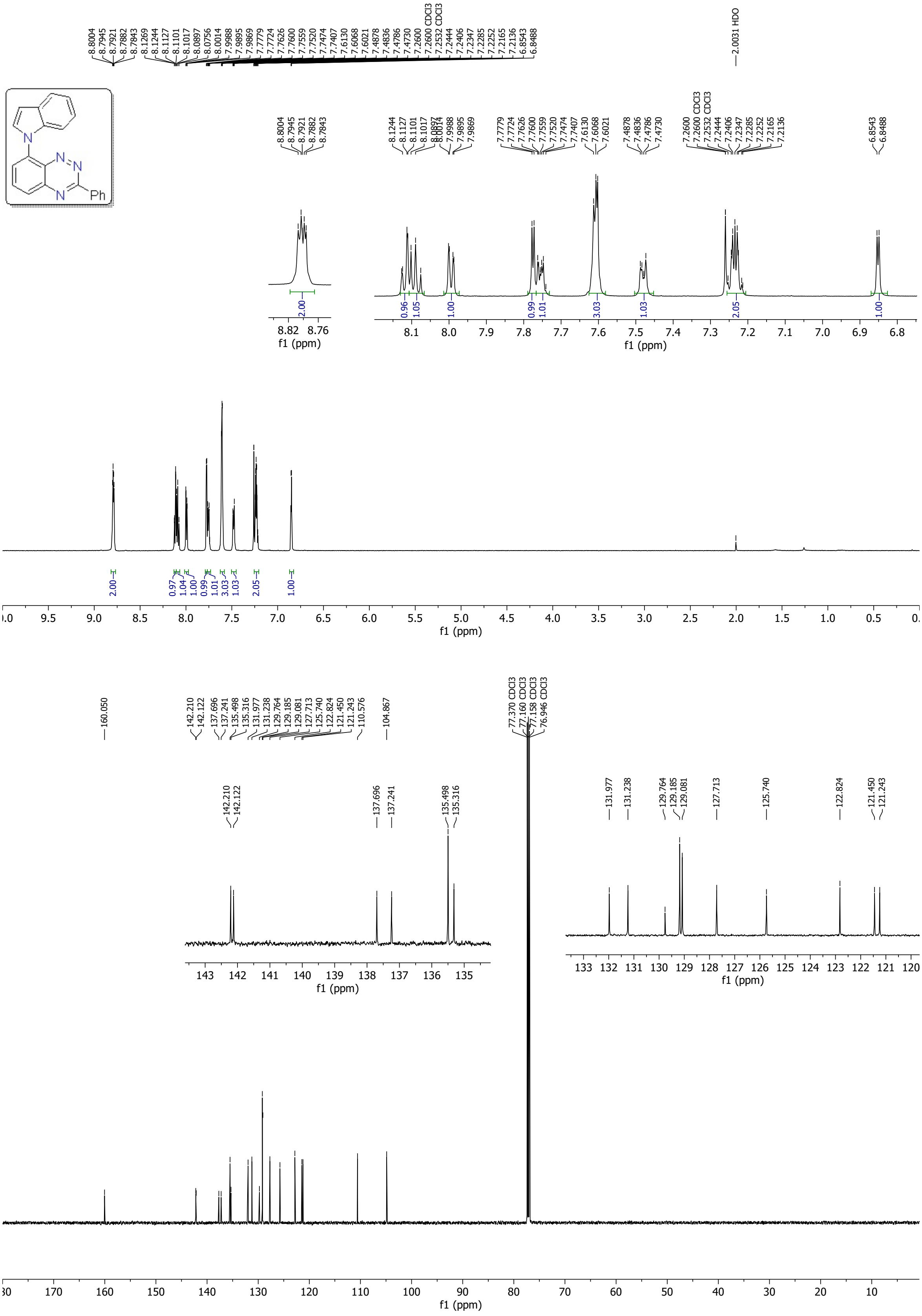

Figure S2. ^{1}H NMR and ^{13}C NMR spectra of 8-(1*H*-indol-1-yl)-3-phenylbenzo[*e*][1,2,4]triazine (**2a**) recorded in $CDCl_3$ at 600 and 151 MHz, respectively.

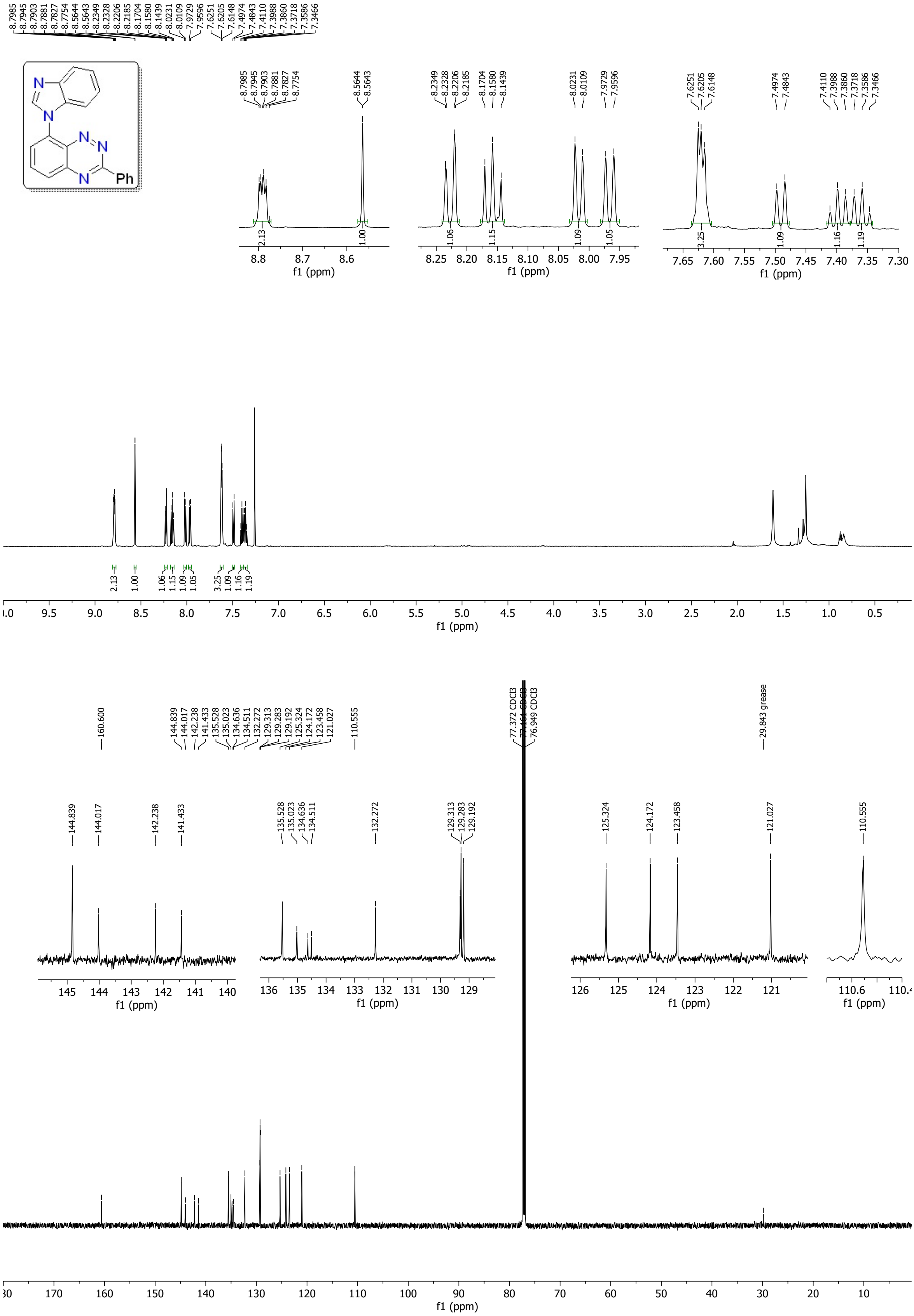

Figure S3. ^{1}H NMR and ^{13}C NMR spectra of 8-(1*H*-benzo[*d*]imidazol-1-yl)-3-phenylbenzo[*e*][1,2,4]triazine (**2b**) recorded in $CDCl_3$ at 600 and 151 MHz, respectively.

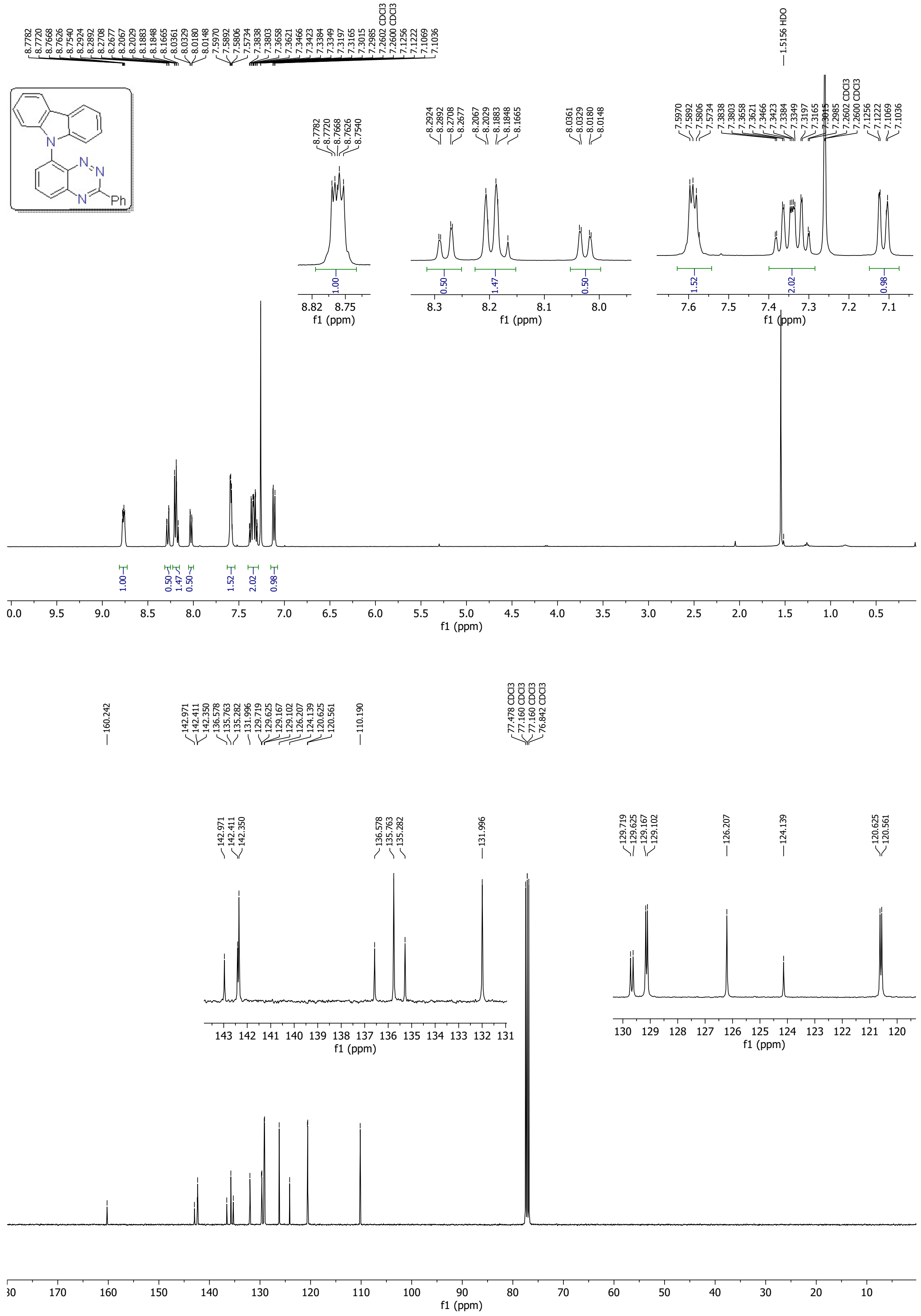

Figure S4. ^{1}H NMR and ^{13}C NMR spectra of 9-(3-phenylbenzo[*e*][1,2,4]triazin-8-yl)-9*H*-carbazole (**2c**) recorded in $CDCl_3$ at 400 and 101 MHz, respectively.

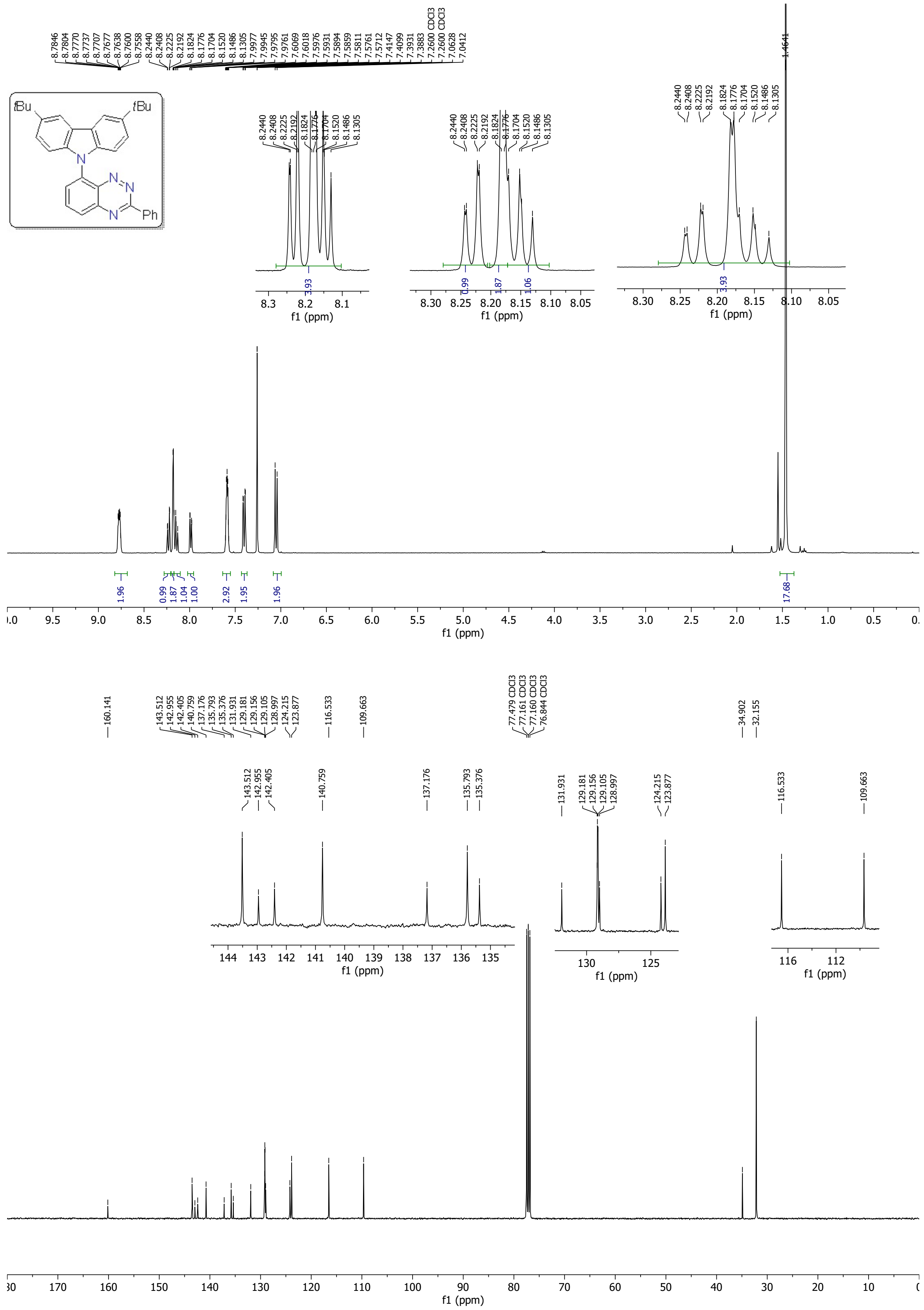

Figure S5. ^{1}H NMR and ^{13}C NMR spectra of 3,6-di-*tert*-butyl-9-(3-phenylbenzo[*e*][1,2,4]triazin-8-yl)-9*H*-carbazole (**2d**) recorded in $CDCl_3$ at 400 and 101 MHz, respectively.

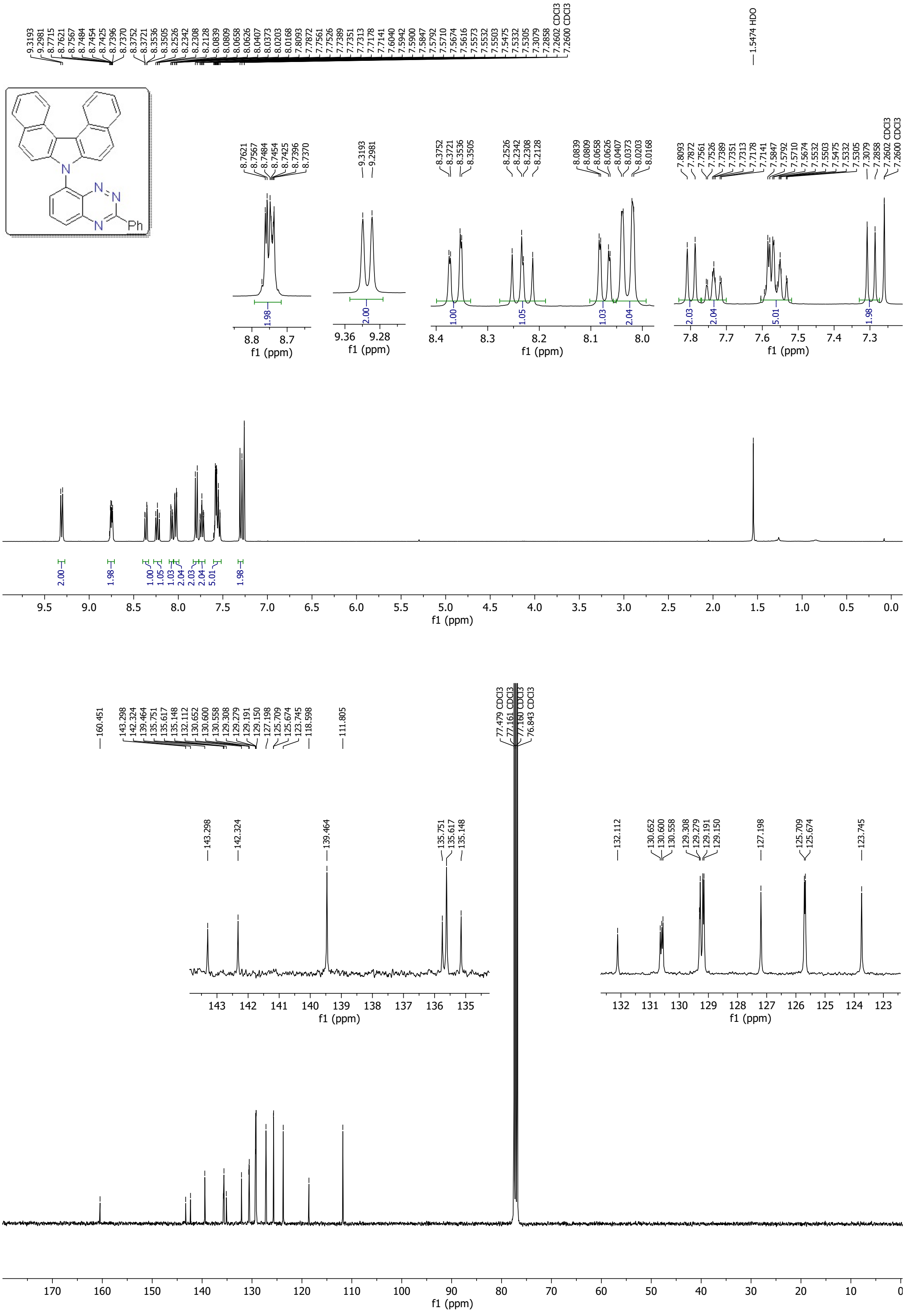

Figure S6. ^{1}H NMR and ^{13}C NMR spectra of 7-(3-phenylbenzo[*e*][1,2,4]triazin-8-yl)-7*H*-dibenzo[*c,g*]carbazole (**2e**) recorded in $CDCl_3$ at 400 and 101 MHz, respectively.

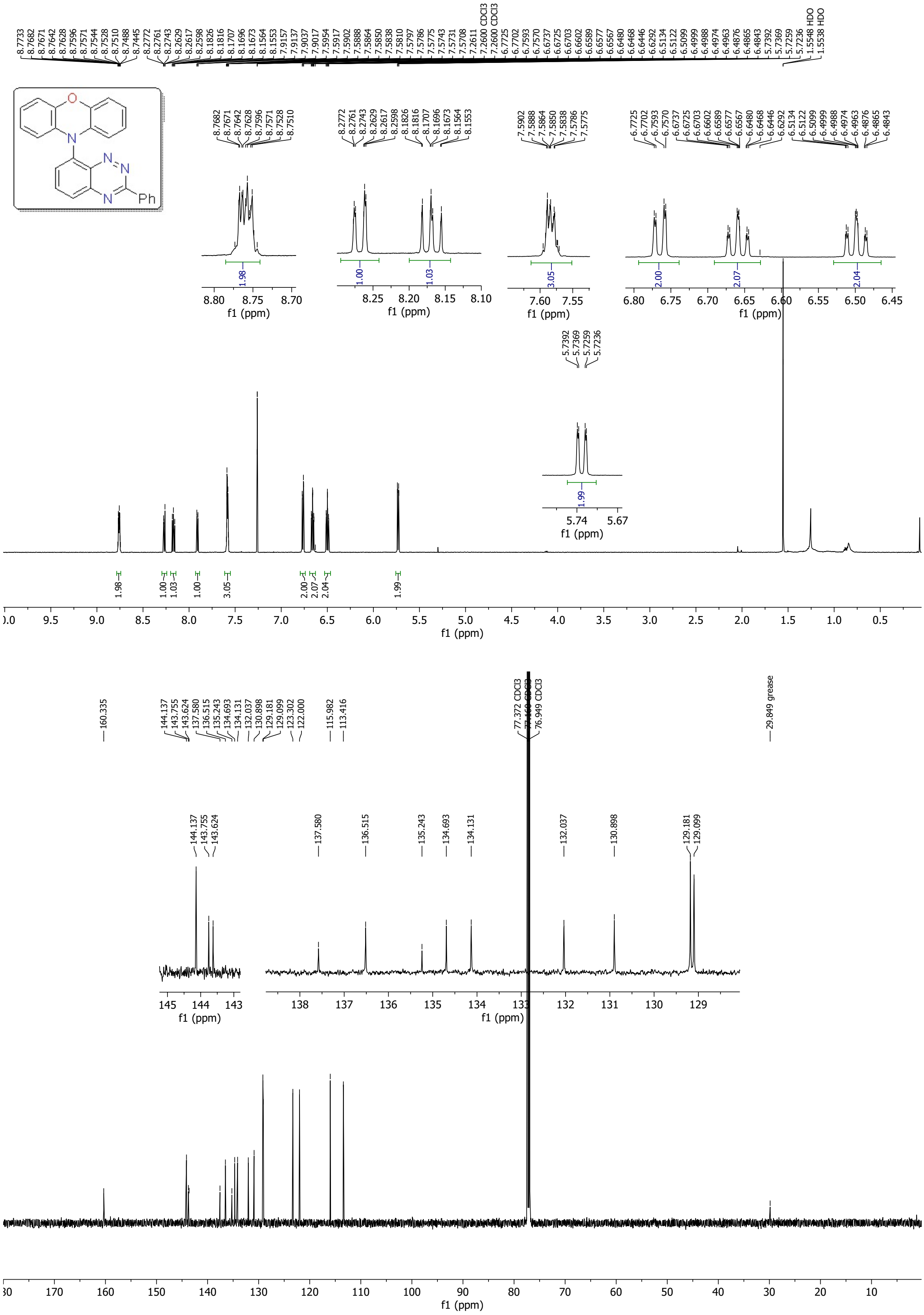

Figure S7. ^{1}H NMR and ^{13}C NMR spectra of 10-(3-phenylbenzo[*e*][1,2,4]triazin-8-yl)-10*H*-phenoxazine (**2f**) recorded in $CDCl_3$ at 600 and 151 MHz, respectively.

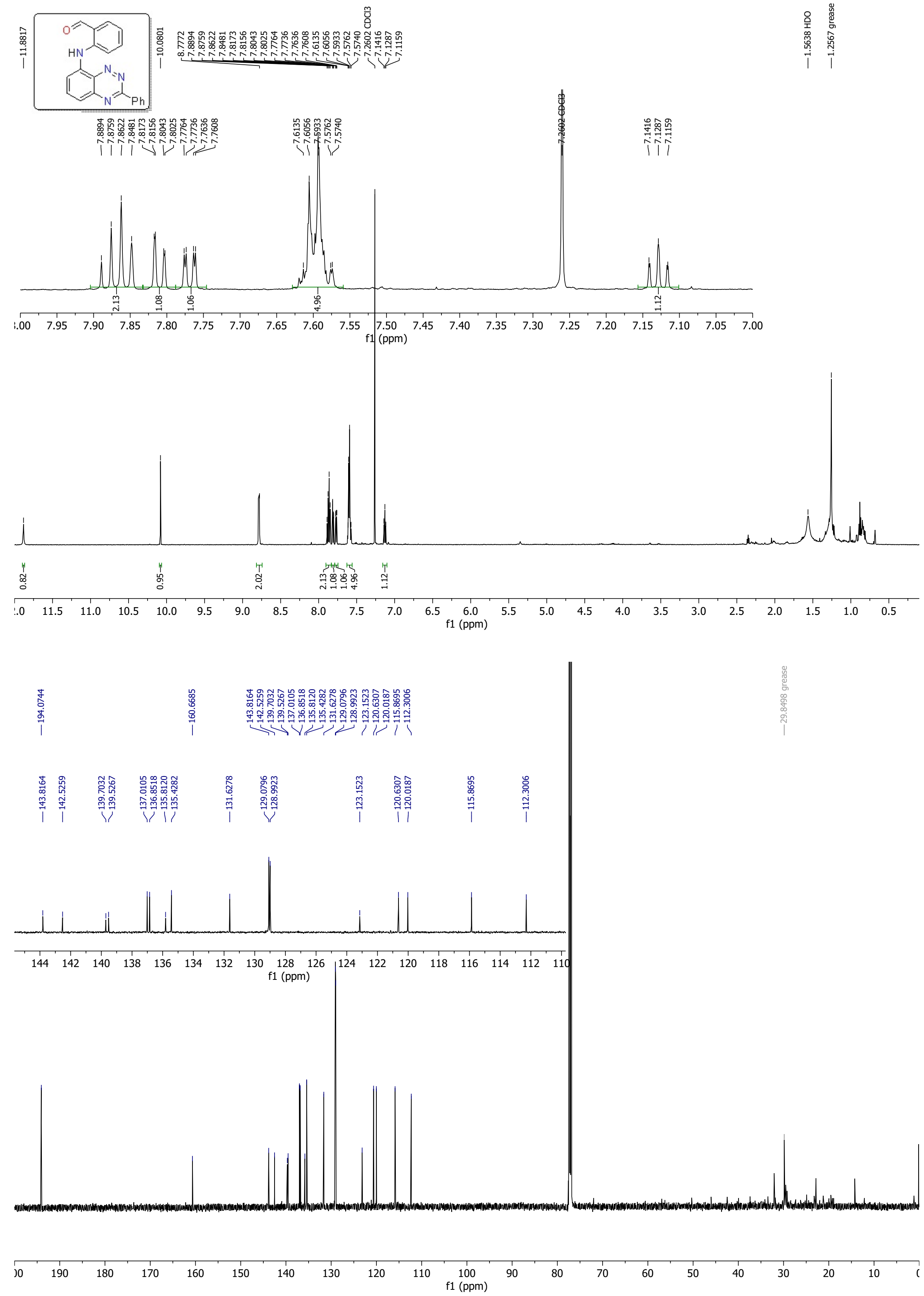

Figure S8. ^{1}H NMR and ^{13}C NMR spectra of 2-((3-phenylbenzo[*e*][1,2,4]triazin-8-yl)amino)benzaldehyde (**5**) recorded in $CDCl_3$ at 600 and 176 MHz, respectively.

4. XRD data collection and refinement for 1d and 1c-*oxo*

Single-crystal XRD measurements for **1d** and **1c-*oxo*** were performed with a Rigaku XtaLAB Synergy, Pilatus 300K diffractometer. The measurements were conducted at 100.0(5) K using the CuK_α radiation (λ=1.54184 Å). The data was integrated using CrysAlisPro program.[3] Intensities for absorption were corrected using multi-scan method as in SCALE3 ABSPACK scaling algorithm implemented in CrysAlisPro program. Additional crystal and refinement information are listed in Table S1. Selected geometrical parameters are provided in Table S2 and compared to those of **A(X=H)** and **B(X=H)**.[2] Selected close contacts for **1d** are presented in Figure S9 and in Table S3. All dimensions and s.u. values are copied as they appear in the CIF.

CCDC: 2327427 and 2327428 contain the supplementary crystallographic data for this paper. The data is provided free of charge by The Cambridge Crystallographic Data Center.

Table S1. Selected Structural Data for **1d** and **1c-*oxo*.**

	**1d**<br>CCDC: 2327427	**1c-*oxo***<br>CCDC: 2327428
Formula	$C_{33}H_{31}N_4$	$C_{25}H_{14}N_4O$
Formula Weight	483.62	386.40
Crystal System	Monoclinic	Monoclinic
Space Group	*C2/c*	*P*2$_1$/n
a/Å	22.1999(3)	7.4588(4)
b/Å	10.4920(1)	23.524(1)
c/Å	21.9787(3)	19.4019(1)
α/°	90	90
β/°	98.613(1)	92.652(4)
γ/°	90	90
Volume/Å^3	5061.6(1)	3400.6(3)
Z	8	8
2θ range for data collection/°	8.056 to 157.474	7.516 to 134.16
Index ranges	-26 ≤ h ≤ 26, -13 ≤ k ≤ 9, -27 ≤ l ≤ 27	-8 ≤ h ≤ 8, -28 ≤ k ≤ 28, -20 ≤ l ≤ 22
No. of measured, independent, and observed [$I>2\sigma(I)$] reflections	33089, 5125, 4376	34713, 5961, 4006
*R*int	0.0298	0.1146
Goodness-of-fit on F^2	1.055	1.060
Final *R* indexes [$F^2 >2\sigma(F^2)$]	R_1=0.0384, wR2= 0.0966	R_1= 0.0616, *wR2*= 0.1542
Final *R* indexes [all data]	R_1=0.0461, wR2= 0.1012	R_1= 0.0948, *wR2*= 0.1756
Data/restraints/parameters	5125/126/396	5961/0/541
Largest diff. peak/hole Å^{-3}	0.27/-0.32	0.32/-0.24

Table S2. Pertinent geometrical parameters for selected structures.[a]

	**1d**	**1c-*oxo***			**A(X=H)**[*b*]	**B(X=H)**[*b*]
	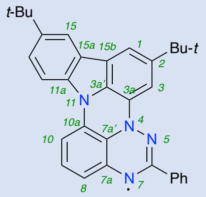	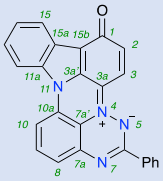			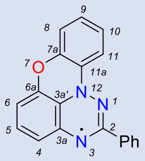	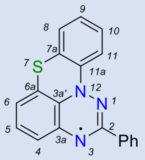
CCDC #	2327427	2327428			1483990	1483989
		A	B			
*d*N5–N4	1.3652(13)	1.366(3)	1.357(3)	*d*N1–N12	1.35(1)	1.368(3)
*d*N5–C6	1.3413(15)	1.333(4)	1.346(4)	*d*N1–C2	1.32(1)	1.331(3)
*d*C6–N7	1.3363(15)	1.332(4)	1.317(4)	*d*C2–N3	1.364(9)	1.340(4)
*d*N7–C7a	1.3772(15)	1.383(4)	1.375(4)	*d*N3–C3a	1.38(1)	1.372(4)
*d*C7a–C7a’	1.4108(16)	1.397(4)	1.422(4)	*d*C3a–C3a’	1.38(1)	1.418(4)
*d*C7a’–C10a	1.4185(16)	1.424(4)	1.420(4)	*d*C3a’–C6a	1.36(1)	1.401(4)
*d*C10a–N11	1.4035(15)	1.387(4)	1.412(3)	*d*C6a–X7	1.39(1)	1.757(3)
*d*N11-C3a’	1.3834(15)	1.385(4)	1.373(4)	*d*X7-C7a	1.381(1)	1.757(3)
*d*N4–C3a	1.3996(15)	1.335(4)	1.360(3)	*d*N12–C11a	1.42(1)	1.420(3)
*d*C6-Ph	1.4904(16)	1.496(4)	1.488(4)	*d*C2-Ph	1.50(1)	1.481(4)
*α*N4-N5-C6	115.13(10)	116.3(2)	115.7(2)	*α*N12-N1-C2	114.1(6)	116.3(2)
*α*N5-C6-N7	128.11(11)	128.4(3)	128.2(3)	*α*N1-C2-N3	130.2(7)	128.1(3)
*α*C3a’-N11-C10a	118.53(10)	118.9(2)	119.0(2)	*α*C7a-X7-C6a	118.5(6)	101.53(13)
*α*C7a’-N4-C3a	119.76(10)	120.4(2)	120.4(2)	*α*C3a’-N12-C11a	118.6(6)	123.2(2)
*θ*N4-N5-C6-N7	0.12(17)	0.7(4)	4.4(4)	*θ*N12-N1-C2-N3	1(1)	-0.8(4)
*θ*C7a’-C10a-N11-C3a’	-6.52(15)	-0.7(4)	2.0(4)	*θ*C3a’-C6a-X7-C7a	0(1)	17.5(3)
*β*triazine-(N12)Ph	5.7	5.7	7.2	*β*triazine-(N12)Ph	3.5	14.3
*β*triazine-(C2)Ph	2.9	5.9	22.4	*β*triazine-(C2)Ph	2.9	30.2

[a] For each structure appropriate systematic numbering scheme is used as shown in table head. *d* - interatomic distance, *α* - interatomic angle, *θ* - dihedral angle, *β* - inter-ring angle. [b] Ref [2].

Table S3. Selected close contacts in the solid-state structure of **1d**.[*a*]

Contact X···Y	$d_{X...Y}$ /Å	$d_{X\text{-}Y\ \text{–VdW}}$ /Å	Calculated spin densities	Type of interaction [*b*]
C(6)···C(10a) [*c*]	3.288(2)	-0.112 [*e*]	+0.021…-0.057	F
C(3)···C(8) [*c*]	3.322(2)	-0.078 [*e*]	+0.094…-0.001	F
C(3a’)-CH_3(tBu) [*d*]	3.286(2)	-0.114 [*e*]	+0.053…0.001	A

[*a*] The systematic numbering scheme of the indolo[3,2,1-*de*][1,2,4]triazino[5,6,1-*kl*]phenazine skeleton for **1d** is used; see pg S8. [*b*] F – ferromagnetic, A –

antiferromagnetic. [c] Symmetry code: -x+$\frac{1}{2}$, -y+$\frac{3}{2}$, -z+1. [d] Symmetry code: -x+1, y, -z+$\frac{3}{2}$. [e] Assuming 1.70 Å for the van der Waals radius of the carbon atom.

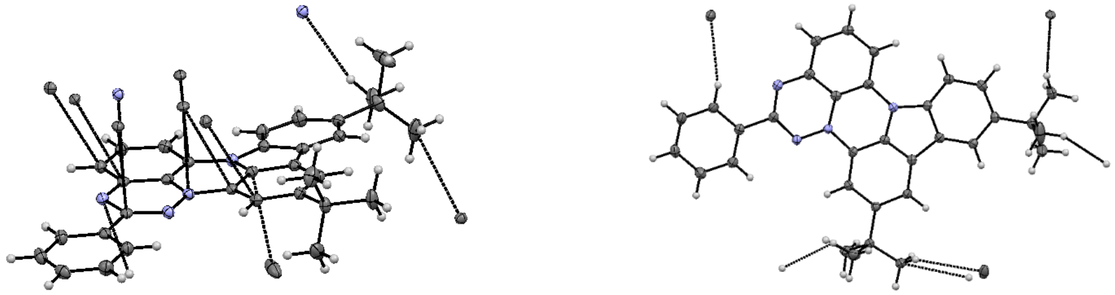

Figure S9. Close contacts in the crystal of **1d**: intradimer (left) and interdimer (right).

Structure solution and refinement

The structures were solved with the ShelXT[4] structure solution program using Intrinsic Phasing and refined by the full-matrix least-squares minimization on F^2 was performed with the ShelXLrefinement package.[5] All non-hydrogen atoms were refined anisotropically and C–H hydrogens were generated geometrically using the HFIX command as in ShelXL. Hydrogen atoms were refined isotropically and constrained to ride on their parent atoms. Two *tert*-butyl groups in **1d** are rotationally disordered over two orientations with occupancy ratios 0.944(2):0.056(2) and 0.861(2):0.139(2). Sums of occupancies of relevant sites were set equal to 1 and refined using free variables. PART instruction was applied to exclude bonding between equivalent disordered atoms. Anisotropic displacement parameters of neighboring disordered atoms were restrained using SIMU and RIGU procedures as in ShelXL. The crystal data and structure refinement descriptors are presented in Table S1. Partial packing diagrams are shown in Figures S10 – S12.

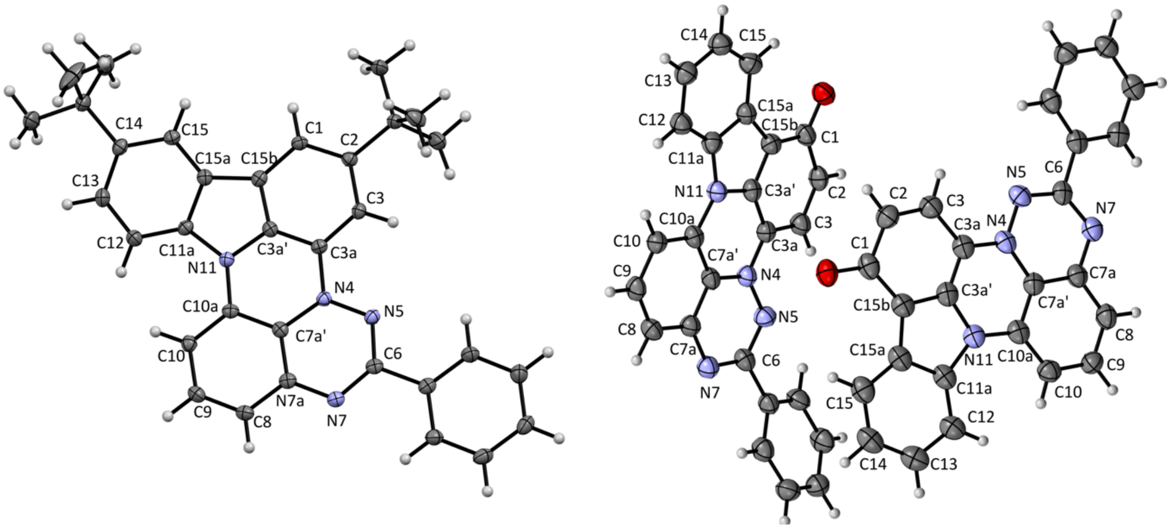

Figure S10. Molecular structures of **1d** and **1c-oxo** with the numbering scheme of the indolo[3,2,1-*de*][1,2,4]triazino[5,6,1-*kl*]phenazine skeleton. The structure of **1c-oxo** contains two molecules in the asymmetric unit. Atomic displacement ellipsoids are drawn at 50% probability level.

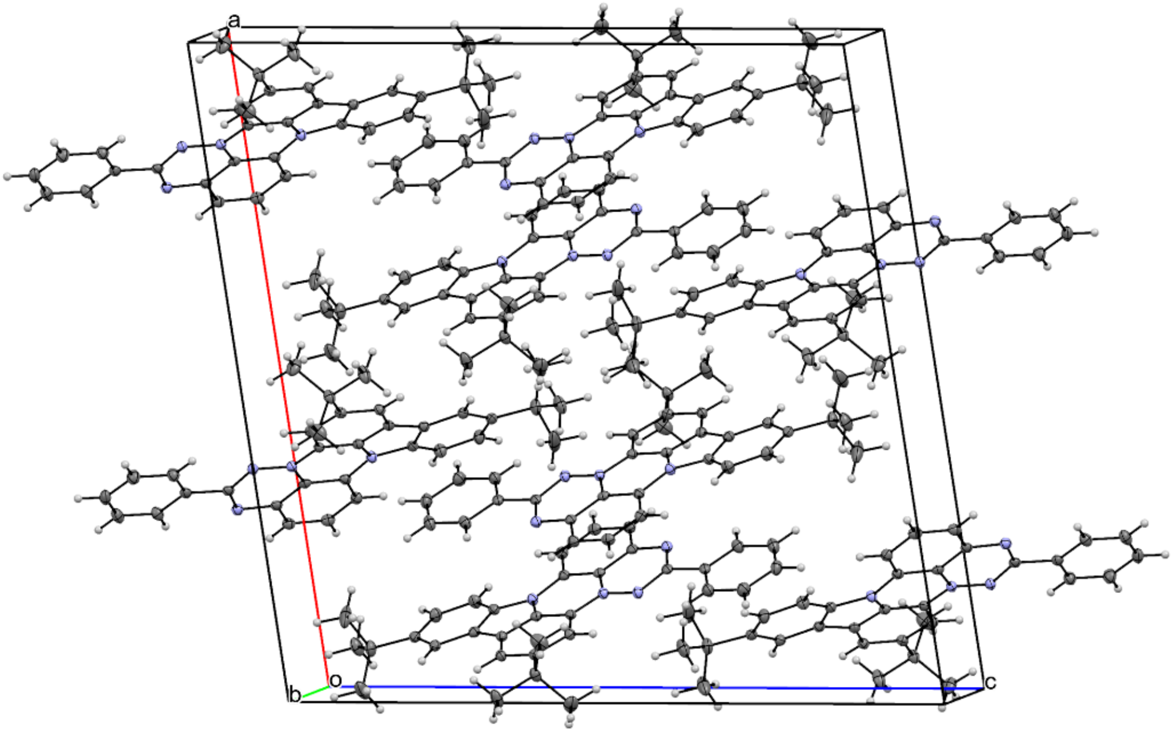

Figure S11. Packing diagram for the unit cell of **1d**.

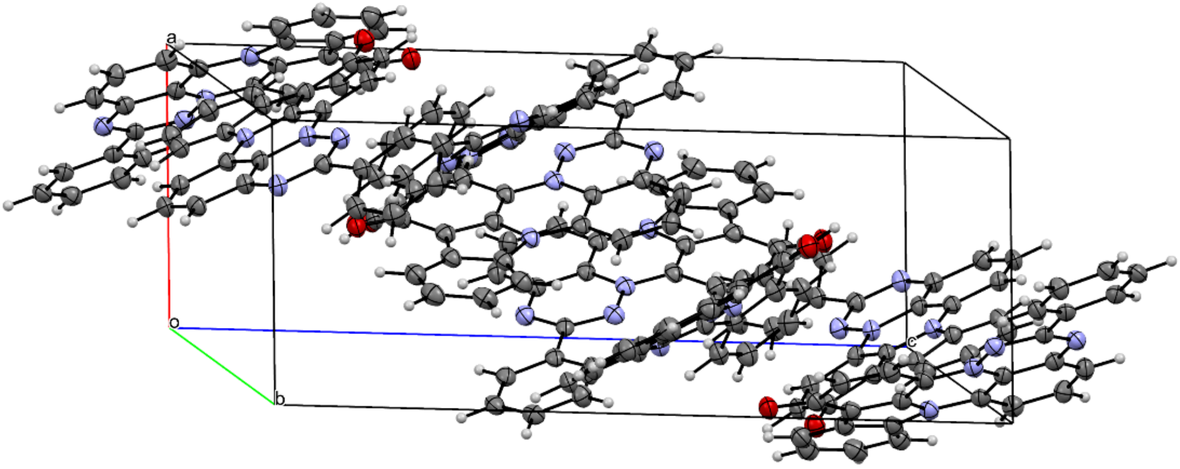

Figure S12. Packing diagram for the unit cell of **1c-*oxo***.

5. Electronic absorption spectroscopy

Electronic absorption spectra for radicals **1** and their precursors **2** were recorded in spectroscopic grade CH_2Cl_2 at concentrations in a range 1–10× 10^{-5} M and fitted to the Beer–Lambert law. Results for radicals **1c** and **1d** are shown in Figures S13 and S14. Figure S15 shows electronic absorption spectra for **1c-*oxo***. Spectra of precursors **2** are shown in Figures S16–S21.

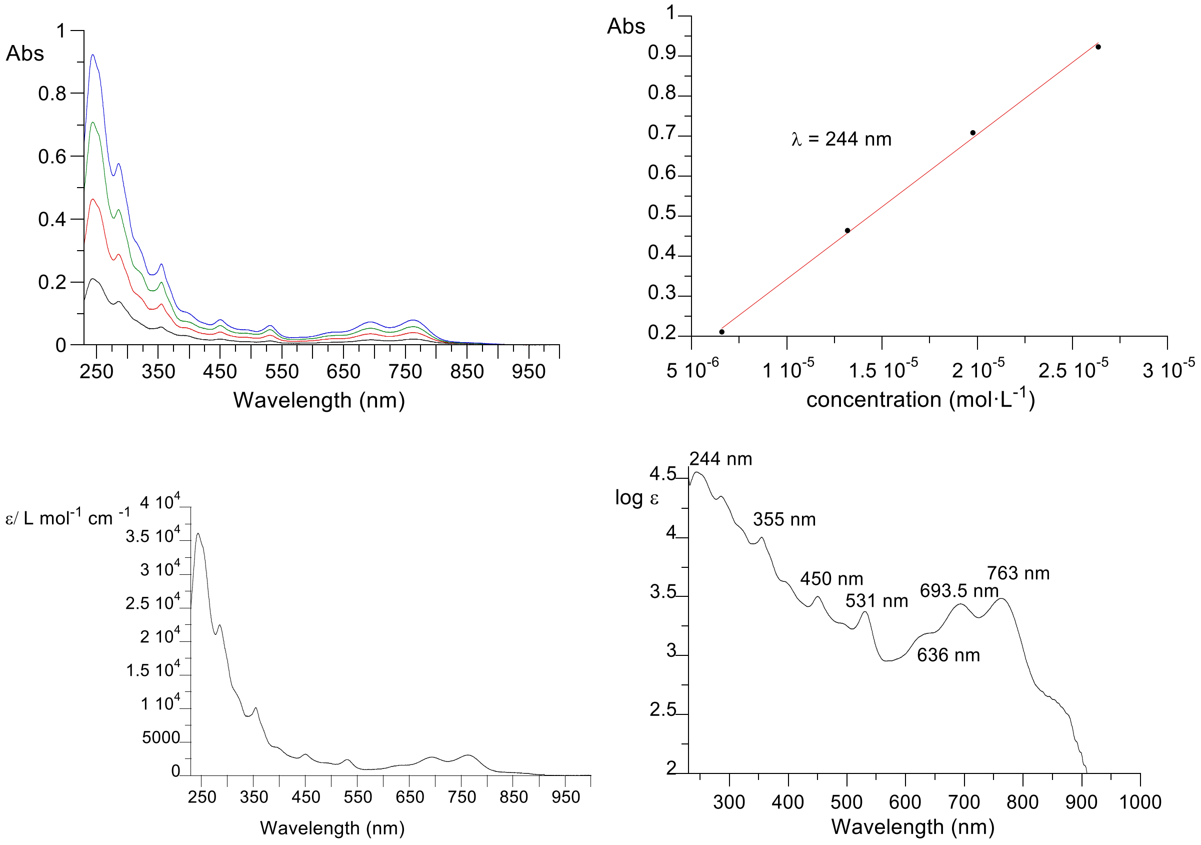

Figure S13. Clockwise: electronic absorption spectra for **1c** in CH_2Cl_2 for four concentrations, determination of molar extinction coefficient ε at λ = 244.0 nm (best fit function: ε = 35169(402)×conc, r^2 = 0.998), molar extinction *log* (ε) plot.

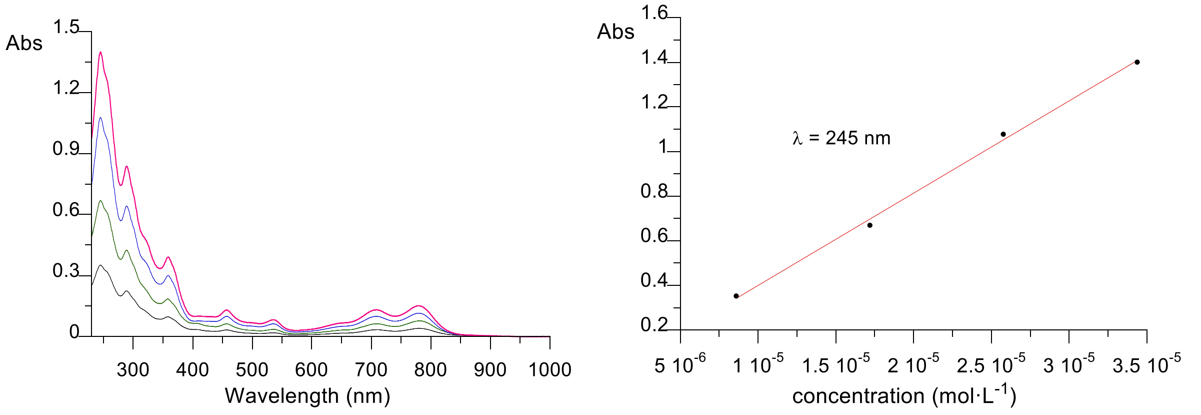

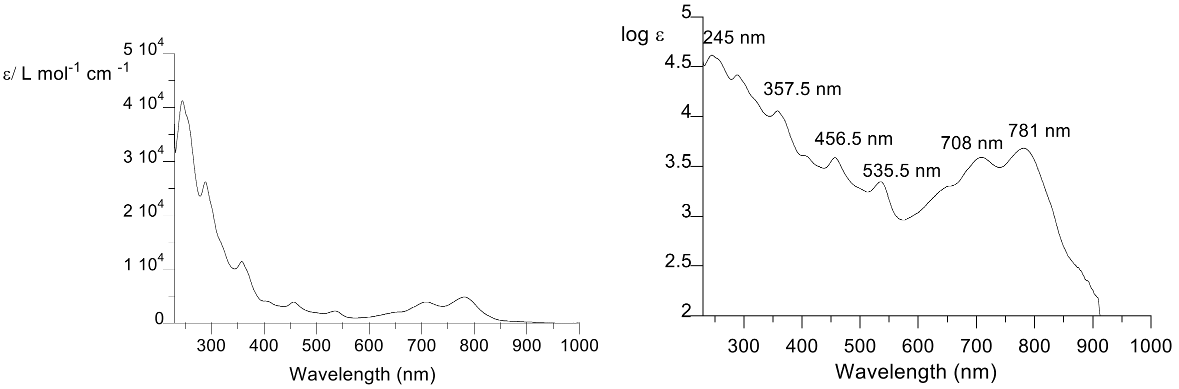

Figure S14. Clockwise: electronic absorption spectra for **1d** in CH_2Cl_2 for four concentrations, determination of molar extinction coefficient ε at λ = 245.0 nm (best fit function: ε = 40831(504)×conc, r^2 = 0.997), molar extinction *log* (ε) plot.

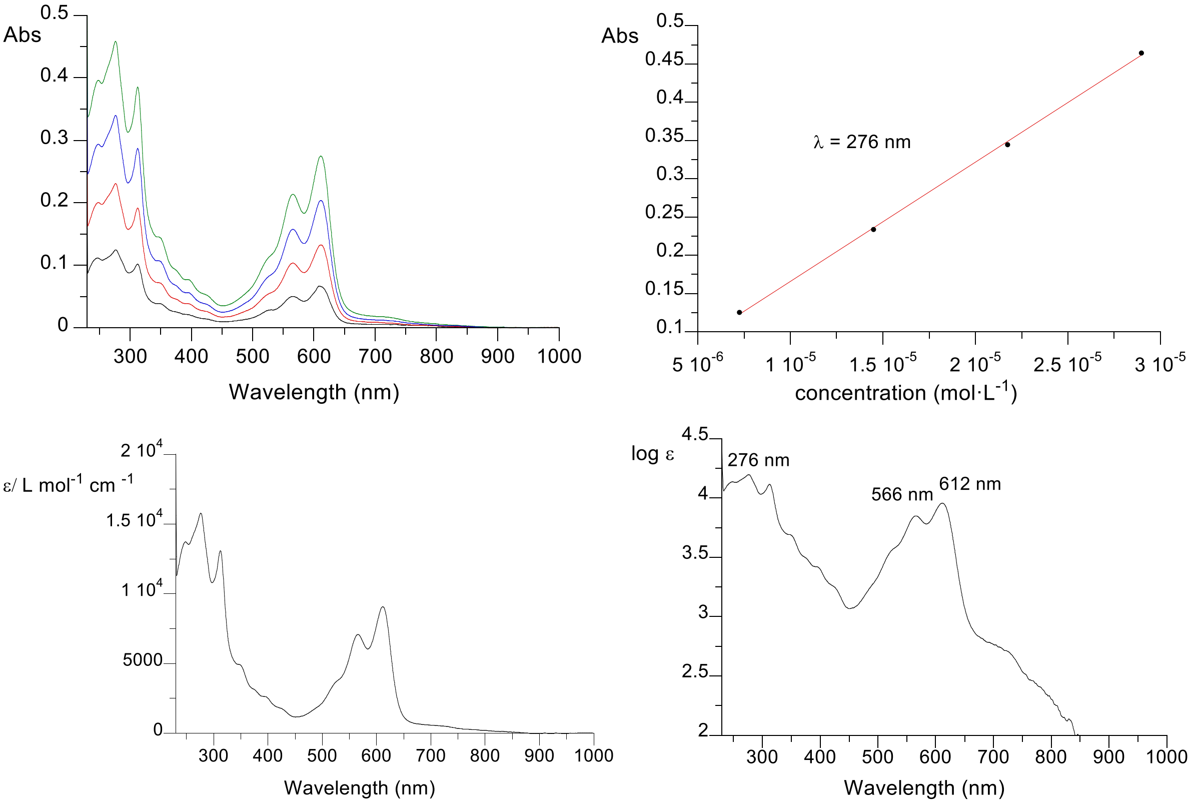

Figure S15. Clockwise: electronic absorption spectra for **1c-*oxo*** in CH_2Cl_2 for four concentrations, determination of molar extinction coefficient ε at λ = 276.0 nm (best fit function: ε = 16030(148)×conc, r^2 = 0.998), molar extinction *log* (ε) plot.

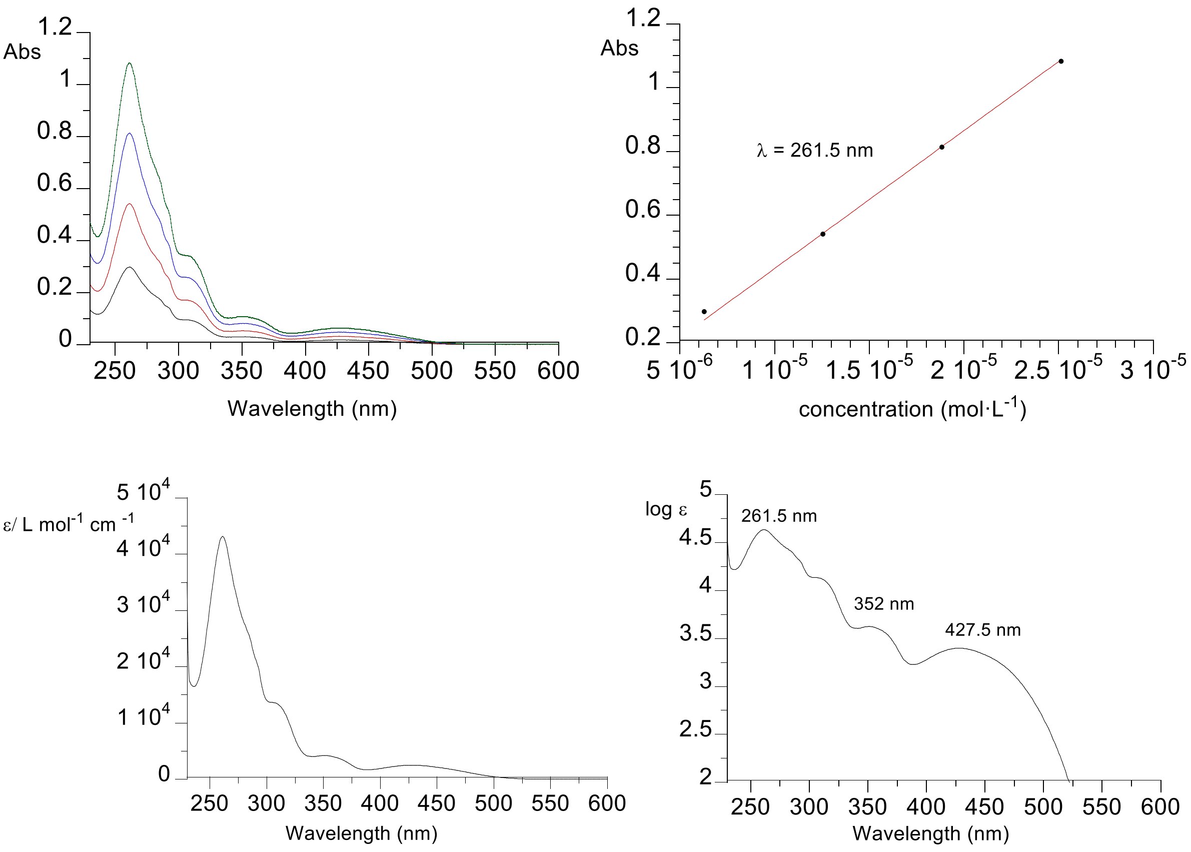

Figure S16. Clockwise: electronic absorption spectra for **2a** in CH_2Cl_2 for four concentrations, determination of molar extinction coefficient ε at λ = 261.5 nm (best fit function: ε = 43285(445)×conc, r^2 = 0.999), molar extinction *log* (ε) plot.

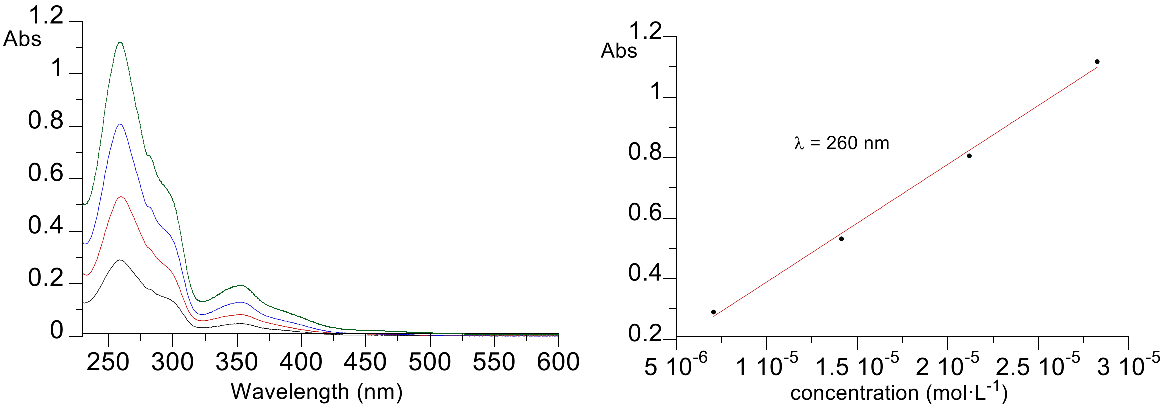

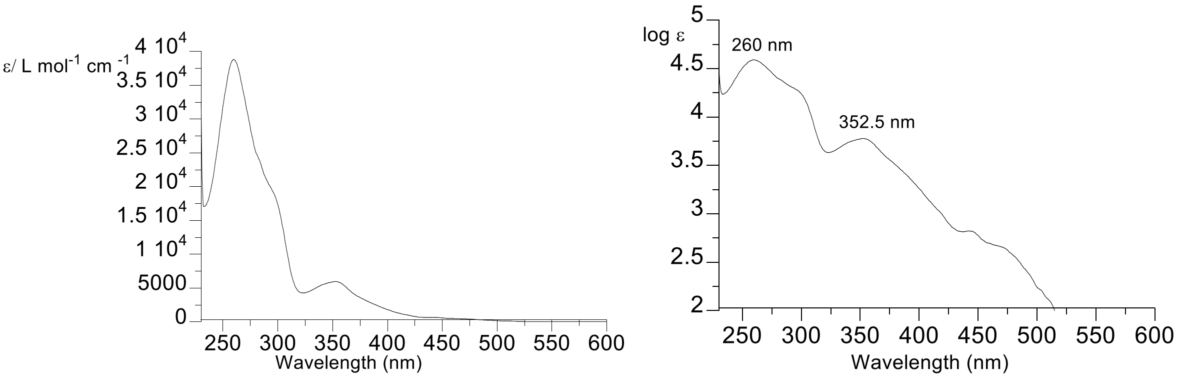

Figure S17. Clockwise: electronic absorption spectra for **2b** in CH_2Cl_2 for four concentrations, determination of molar extinction coefficient ε at λ = 260.0 nm (best fit function: ε = 38896(529)×conc, r^2 = 0.9984), molar extinction *log* (ε) plot.

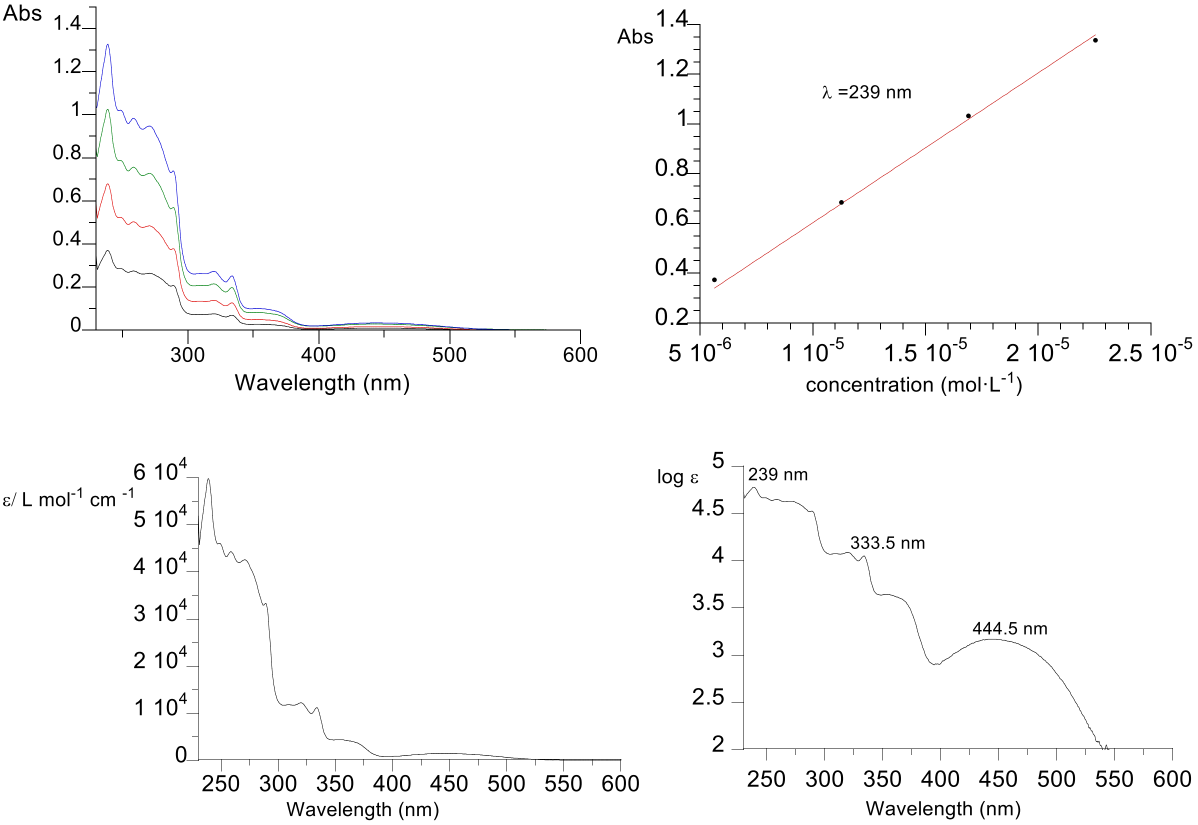

Figure S18. Clockwise: electronic absorption spectra for **2c** in CH_2Cl_2 for four concentrations, determination of molar extinction coefficient ε at λ = 239.0 nm (best fit function: ε = 60247(792)×conc, r^2 = 0.9983), molar extinction *log* (ε) plot.

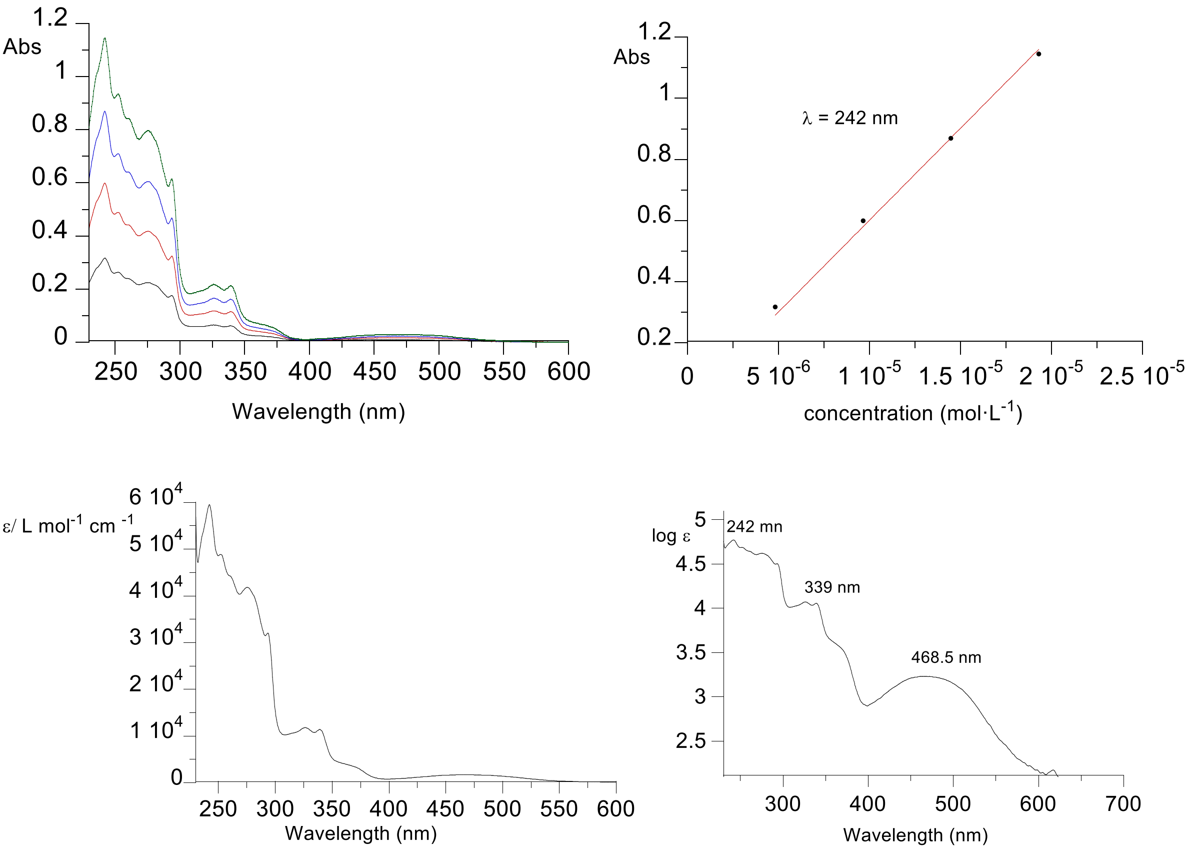

Figure S19. Clockwise: electronic absorption spectra for **2d** in CH_2Cl_2 for four concentrations, determination of molar extinction coefficient ε at λ = 242.0 nm (best fit function: ε = 60138(794)×conc, r^2 = 0.998), molar extinction *log* (ε) plot.

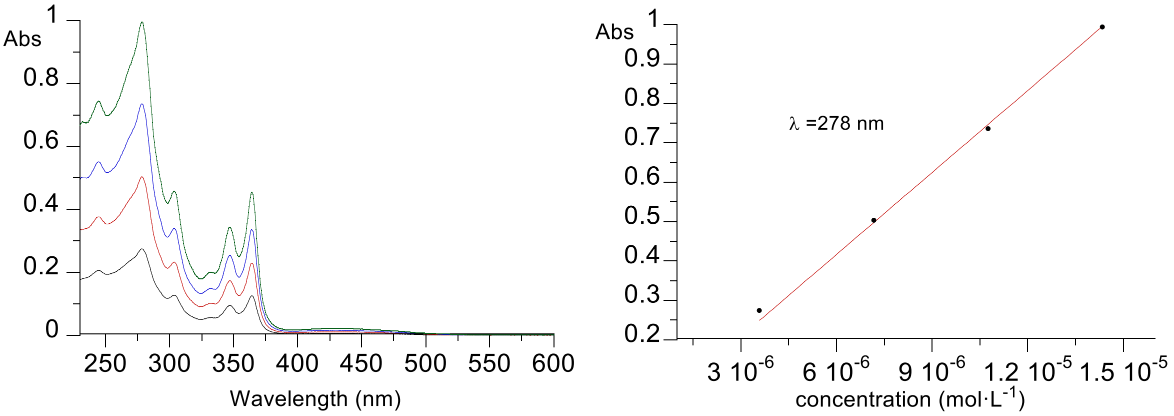

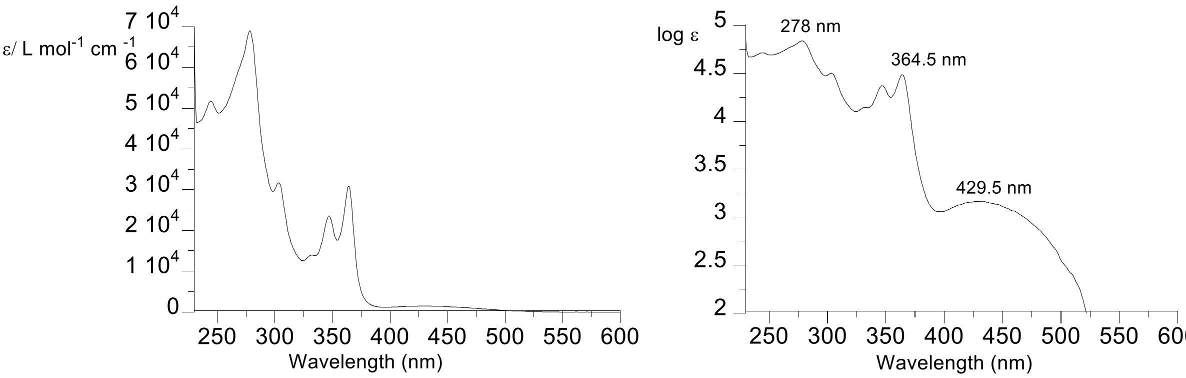

Figure S20. Clockwise: electronic absorption spectra for **2e** in CH_2Cl_2 for four concentrations, determination of molar extinction coefficient ε at λ = 278.0 nm (best fit function: ε = 69413(830)×conc, r^2 = 0.9986), molar extinction *log* (ε) plot.

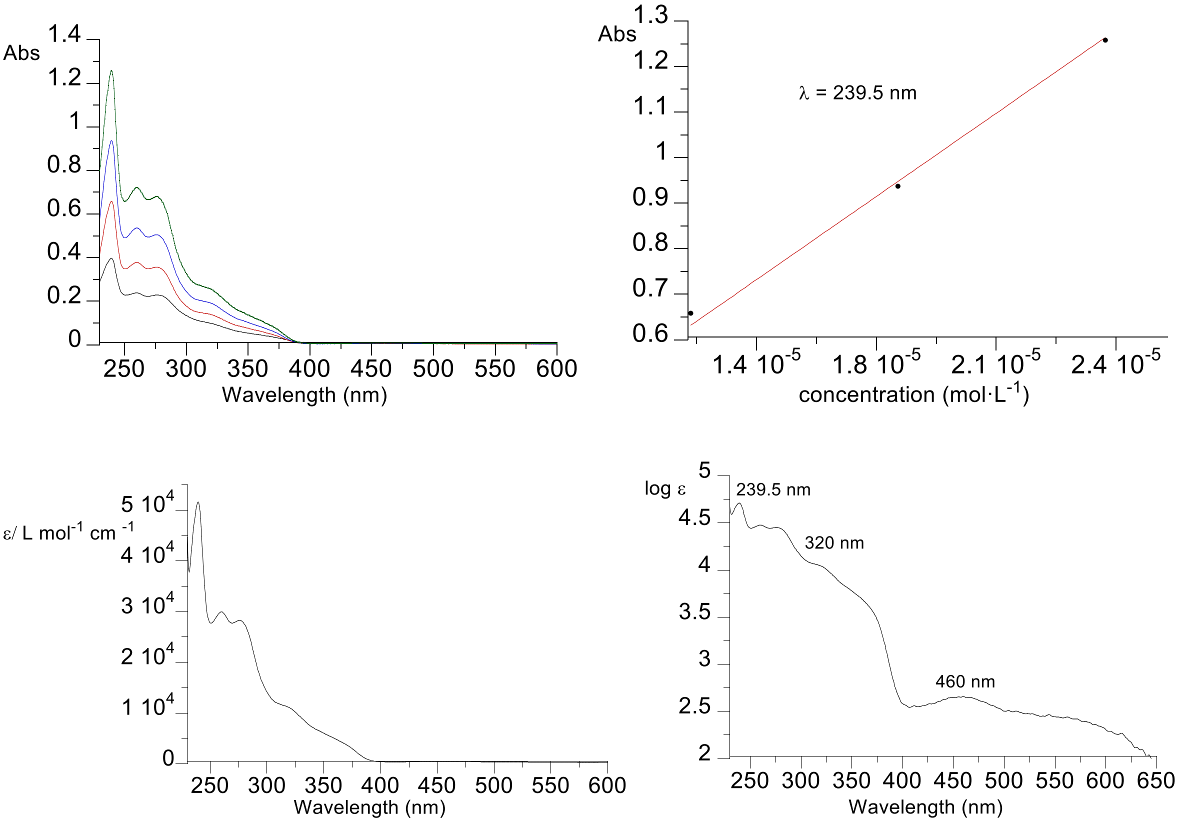

Figure S21. Clockwise: electronic absorption spectra for **2f** in CH_2Cl_2 for four concentrations, determination of molar extinction coefficient ε at λ = 239.5 nm (best fit function: ε = 52258(636)×conc, r^2 = 0.998), molar extinction *log* (ε) plot.

6. Stability of radicals 1c and 1d towards atmospheric oxygen

Oxidative stability of radicals **1c** and **1d** was investigated in CH_2Cl_2 solutions in a quartz cuvette and the absorbance at 762 nm (for **1c**) and 780 nm (for **1d**) was measured periodically. The cuvettes were filled up to 2/3 of the volume with the solution and open daily

to exchange air atmosphere. If needed, fresh solvent was added to maintain the original volume of the solution before each measurement. Results are shown in Figures S22 and S23.

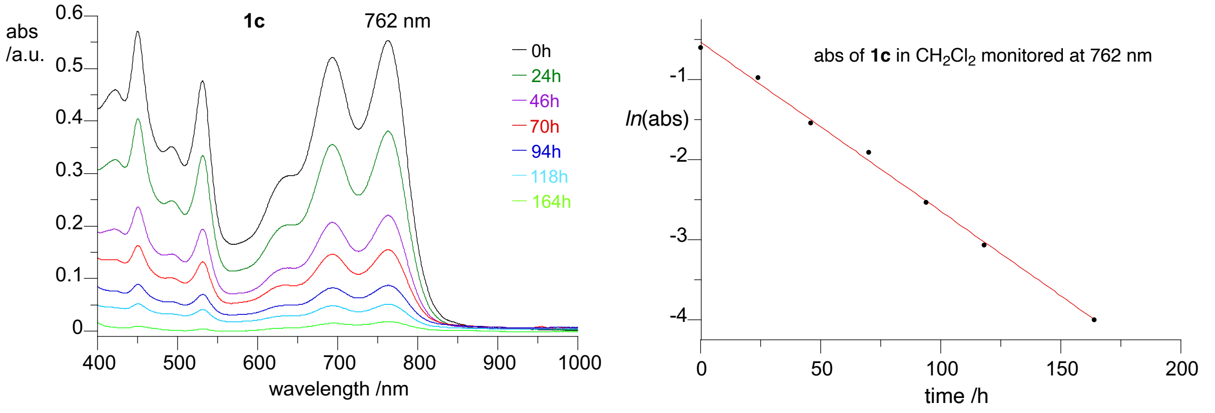

Figure S22. Left: electronic absorption spectra measured for radical **1c** in CH_2Cl_2 solution exposed to air at several time intervals at 273 K. Right: first-order decay of the absorption peak at 762 nm. Best fit line: $ln(\text{abs}) = -0.53(4)–0.0211(5) \times t$, $r^2 = 0.997$.

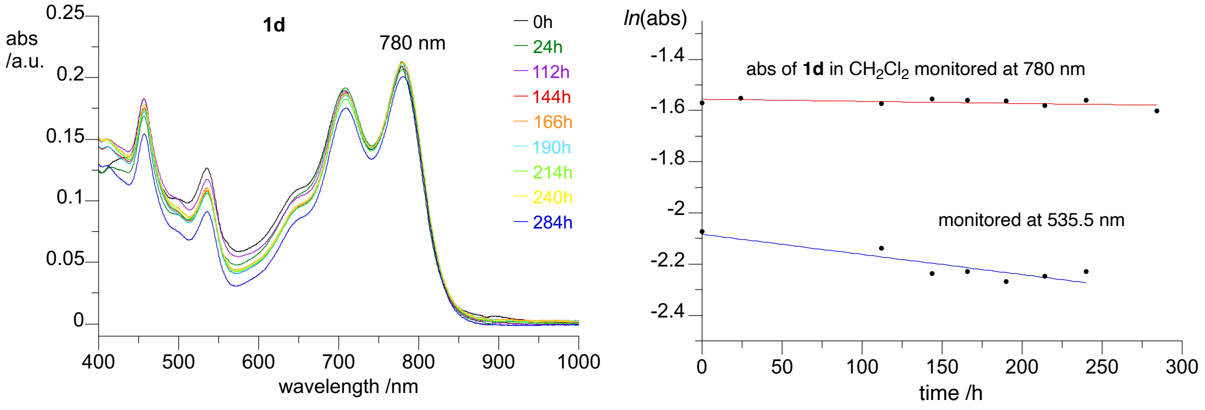

Figure S23. Left: electronic absorption spectra measured for radical **1d** in CH_2Cl_2 solution exposed to air at several time intervals at 273 K. Right: first-order decay of the absorption peak at 780 nm (red) and 535.5 nm (blue). Best fit lines: $ln(\text{abs}_{780}) = -1.556–8.38\times10^{-5} \times t$, $r^2 = 0.28$, and $ln(\text{abs}_{535}) = -2.08–7.9\times10^{-4} \times t$, $r^2 = 0.79$.

7. Electrochemical results

The electrochemical characterization of selected radicals was conducted using Autolab PGSTAT128N potentiostat/galvanostat instrument in dry and degassed CH_2Cl_2 (concentration 0.5 mM) in the presence of $[n\text{-}Bu_4N]^+[PF_6]^-$ as an electrolyte (concentration 50 mM) using glassy carbon as the working electrode and Ag/AgCl as the reference electrode with a scan rate of 50 mV s^{-1} at *ca.* 20 °C. In the end of each measurement ferrocene was added and the peak potentials were referenced to the Fc/Fc^+ couple (0.46 V *vs* SCE).[6]

Cyclic voltammetry (CV) plots are shown in Figures S24 and S25 and numerical result are shown in Table S4. A correlation of $E_{1/2}^{0/+1}$ with Hammett parameters[7] for model substituents is shown in Figure S26.

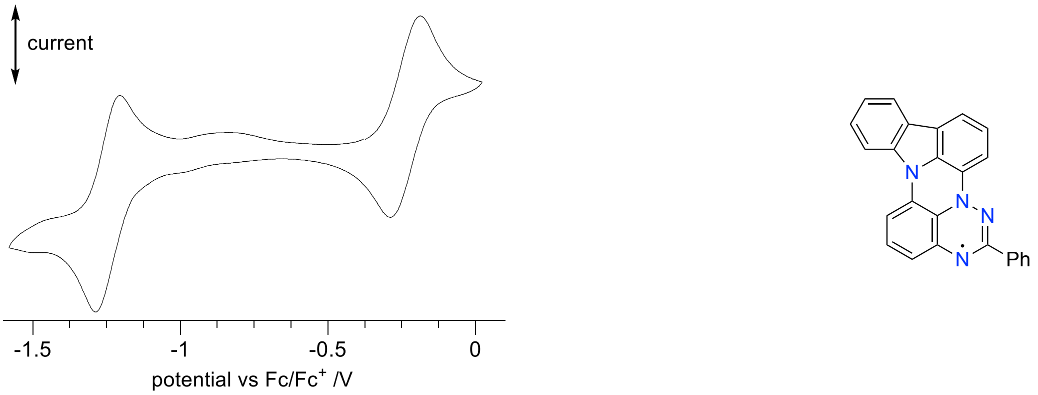

Figure S24. Cyclic voltammogram for **1c**.

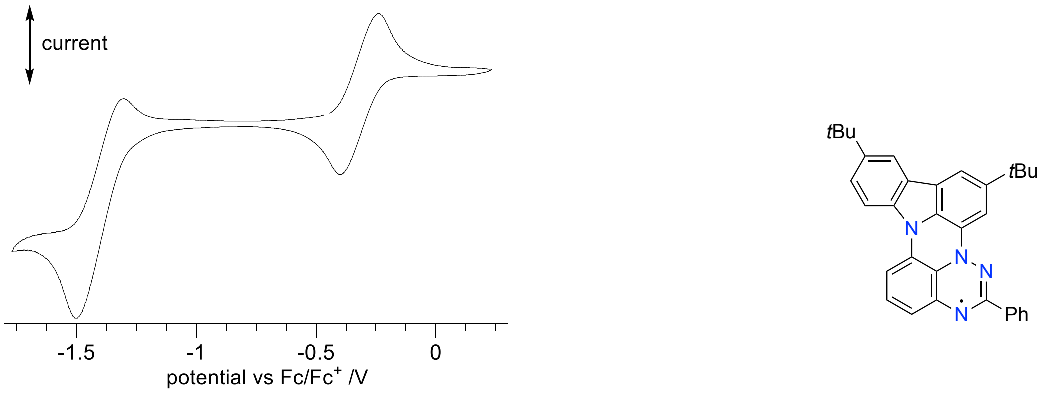

Figure S25. Cyclic voltammogram for **1d**.

Table S4. Cyclic voltammetry data for selected radicals in CH_2Cl_2.[a]

Radical	$E_{1/2}^{-1/0}$ /V	$E_{1/2}^{0/+1}$ /V	E_{cell} /V [a]
**A(X=H)**[b]	-1.317	-0.154	1.163
**B(X=H)**[b]	-1.202	-0.112	1.090
**1c**	-1.243	-0.238	1.005
**1d**	-1.404	-0.320	1.085

[a] Potential *vs* the Fc/Fc+ couple.[b] Ref.[8]

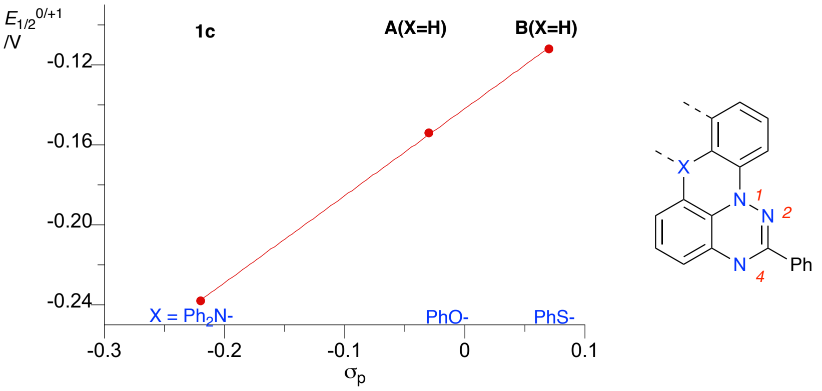

Figure S26. Correlation of $E_{1/2}^{0/+1}$ with Hammett parameter σ_p for model substituents X (blue). Best fitting functions: $a_{N1} = 0.435(6)\times E_{1/2}^{0/+1} - 0.142(1)$, $r^2 = 0.999$.

8. EPR spectroscopy

EPR spectra for radicals **1c** and **1d** were recorded on an X-band EMX-Nano EPR spectrometer at room temperature in dilute and degassed solutions in benzene. The microwave power was set using the Power Sweep program below the saturation of the signal, modulation frequency of 100 kHz, modulation amplitude of 0.5 G_{pp} and spectral width of 100 G. Accurate g-values were obtained using TEMPO as EMX-Nano internal standard. Simulations of the spectra were performed with *Easy Spin* (Matlab) using DFT results (*vide infra*) as the starting point including all nitrogen and 6 hydrogen atoms. The resulting *hfcc* values were perturbed several times until a global minimum for the fit was achieved. For consistency, the previously reported[9] spectrum for **A(X=H)** and a newly recorded spectrum of **B(X=H)** were resimulated using the same parameters. Experimental and simulated spectra for **1c** and **1d** are shown in Figures S27 and S28 and resulting *hfcc* are listed in Table S5.

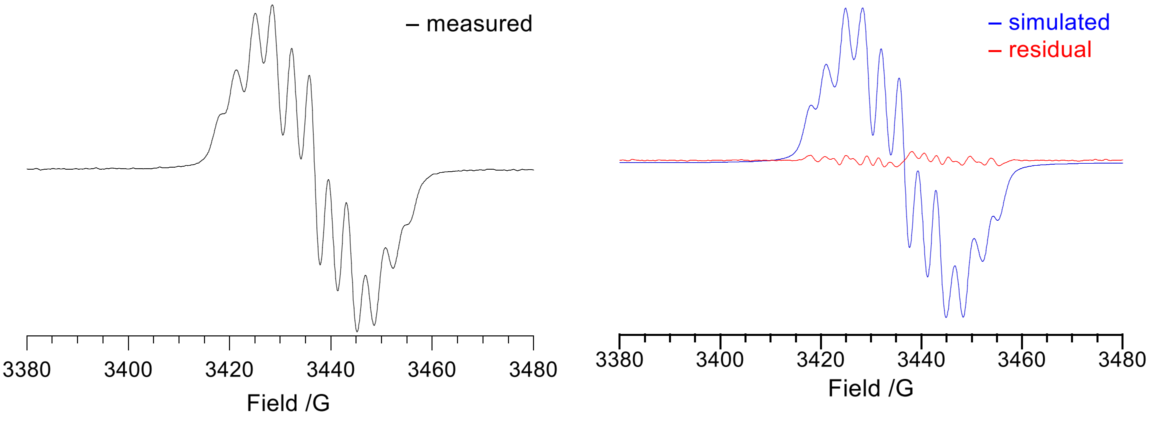

Figure S27. Experimental (black, left), simulated (blue, right) and difference (red, right) spectra for **1c** recorded in benzene at *ca* 20 °C.

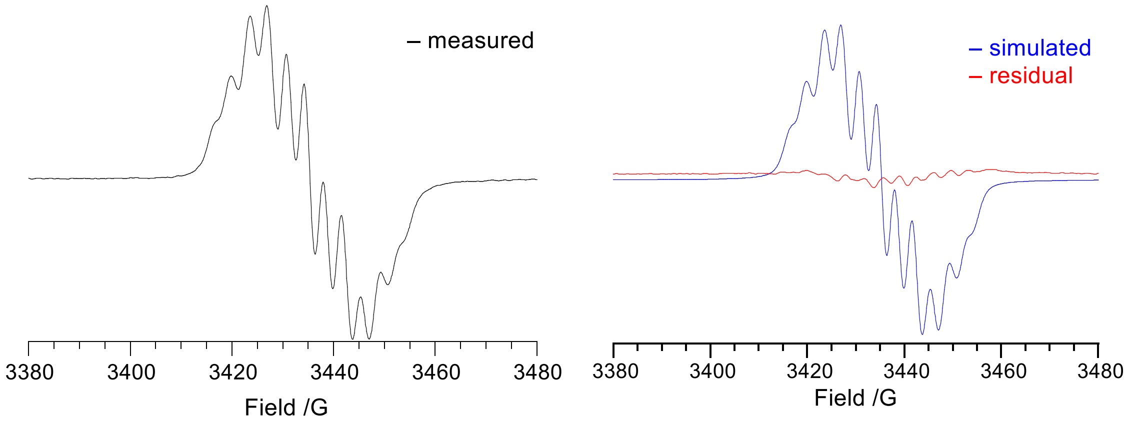

Figure S28. Experimental (black, left), simulated (blue, right) and difference (red, right) spectra for **1d** recorded in benzene at *ca* 20 °C.

Table S5. Experimental hyperfine coupling constants (G) for selected radicals in benzene at *ca*. 20 °C.

atom	A (X=H) [a]	B (X=H)	1c	1d
$a_{N(11)}$	7.53	7.27	7.08	7.14
$a_{N(1)}$	4.35	4.39	4.15	4.00
$a_{N(3)}$	4.42	4.43	4.02	4.00
a_N	-	-	0.35	0.36
a_H	1.88	1.39	2.77	2.79
a_H	1.68	1.26	2.31	2.47
a_H	1.09	1.21	0.34	0.36
a_H	1.07	1.10	0.32	0.35
a_H	1.07	1.10	0.29	0.34
a_H	1.07	0.75	0.29	0.34
g	2.0026	2.0040	2.0036	2.0044

[a] Experimental data for simulation taken from ref [9]

Figure S29 shows correlations of selected hfcc with Hammett parameters[7] for model substituents.

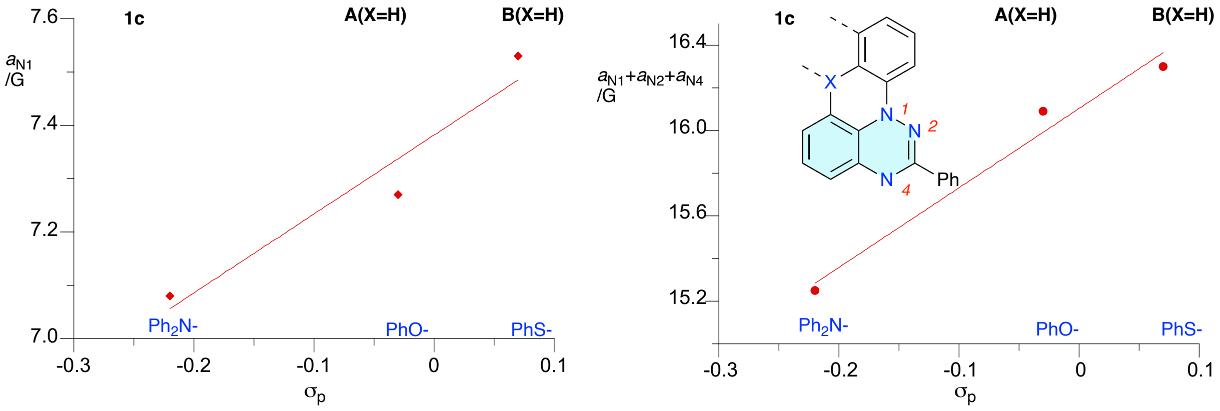

Figure S29. Correlation of a_{N1} *hfcc* (left) and sum of a_N (right) with Hammett parameter σ_p for model substituents (blue). Best fitting functions: $a_{\mathrm{N1}} = 1.5(4)\times\sigma_\mathrm{p} + 7.38(5)$, $r^2 = 0.930$; $\Sigma a_\mathrm{N} = 3.7(6)\times\sigma_\mathrm{p} + 16.10(8)$, $r^2 = 0.976$.

9. SQUID magnetometry

Magnetic susceptibility of polycrystalline sample of radical **1d** was measured in a paramagnetic cavity of known background (a two-part Delrin holder, Figure S30) as a function of temperature in the cooling mode (300 K→ 2 K) with a sweep rate of 0.5 K min^{-1} at 0.6 T, using a SQUID magnetometer (Quantum Design MPMS-XL-7T). Measurements of magnetization *M vs H* for **1d** were conducted at 2 K for several magnetic field strengths (0.05 –7 T).

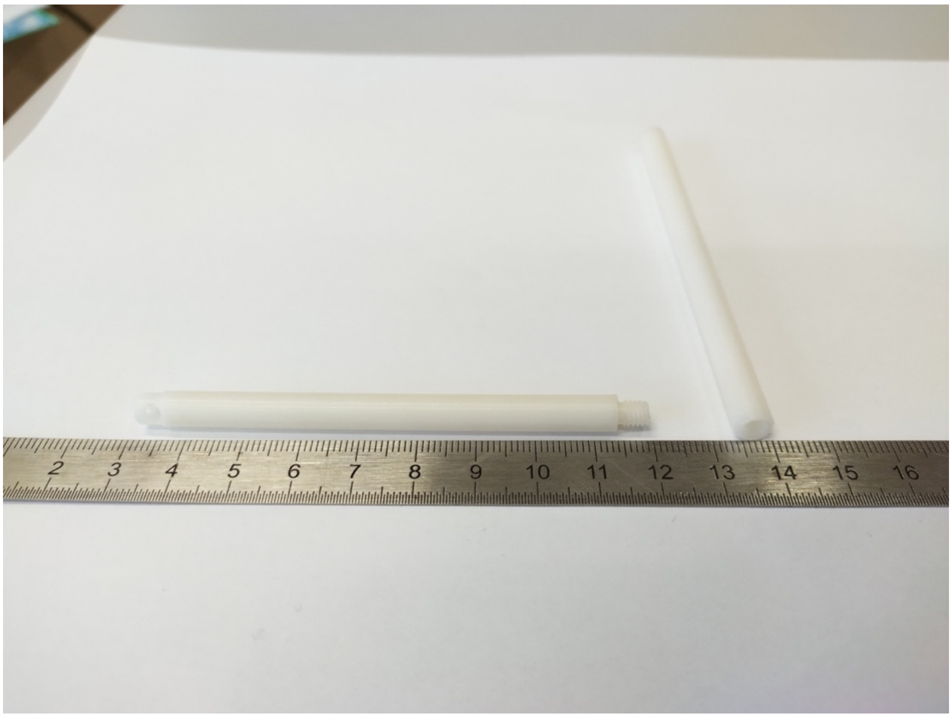

Figure S30. Delrin sample holder used for SQUID measurements.

Raw data workup

The magnetic contribution of the sample holder was removed numerically from the raw data (voltage *vs* sample position, $U(x)$) by subtracting the $U(x)$ contribution for the empty holder in the entire temperature range. The holder brings a large, temperature-constant paramagnetic component.

Analysis for mono-radical **1d**

A microcrystalline sample of derivative **1d** (m = 8.53 mg, 1.764×10^{-5} mol, M_w = 483.64 g mol^{-1}) was analyzed at 0.6 T in the 300–2 K temperature range. Total molar magnetic susceptibility $\chi_{tot}(T)$ and $\chi_{tot}T(T)$ plots are shown in Figures S31 and S32, respectively.

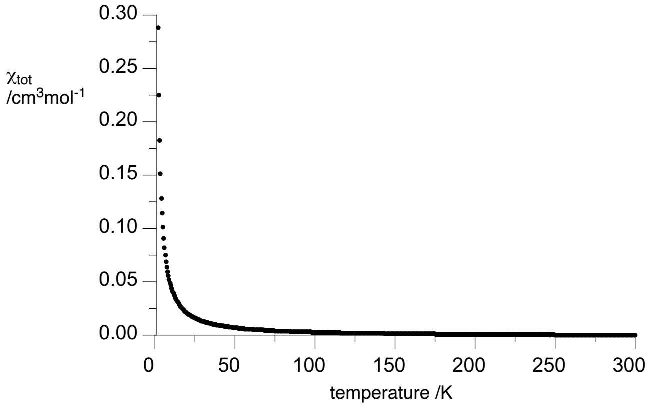

Figure S31. χ_{tot} *vs T* plot for **1d**.

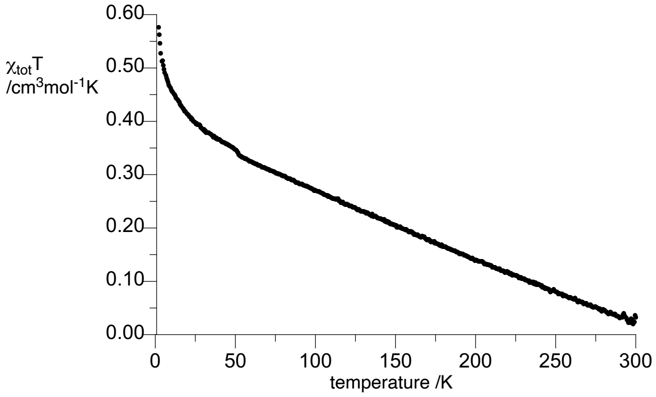

Figure S32. $\chi_{tot}T$ *vs T* plot for **1d**.

The diamagnetic correction for the sample was estimated from the linear portion of high temperature plot χ_{tot}•T *vs* T assuming ideal paramagnetic behavior of the sample using the Curie law (eq S1).

$$\chi_{tot} \bullet T = (\chi_p + \chi_{dia}) \bullet T = C + \chi_{dia} \bullet T \qquad \text{(eq S1)}$$

where C = 0.375 $cm^3mol^{-1}K$ for an ideal paramagnet (S = ½).

Fitting the high temperature portion of the data gave the diamagnetic susceptibility χ_d of –11.95(3)×10^{-4} cm^3mol^{-1} (Figure S33). The total magnetic susceptibility χ_{tot} was corrected for χ_d and the resulting paramagnetic susceptibility is plotted as $\chi_pT(T)$ in Figure S34. The observed bump on the $\chi_pT(T)$ plot at about 50 K is attributed to the presence of small amounts of oxygen (air) in the holder's closed cavity.

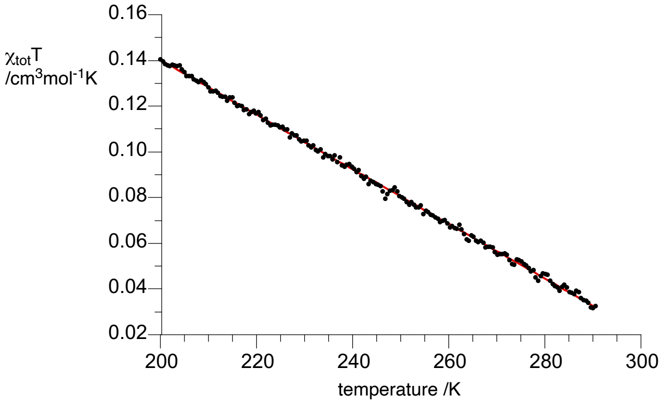

Figure S33. High temperature portion of the $\chi_{tot}T$ *vs* T plot for **1d**. The best fitting function: $\chi_{tot}T$ = -0.001196(3)×T+0.380(1), r^2 = 0.999.

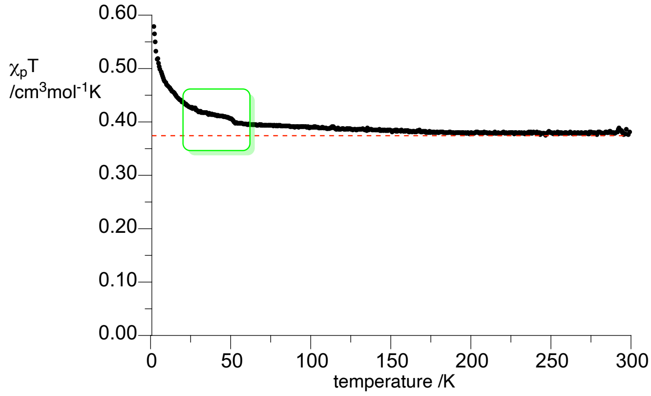

Figure S34. χ_pT *vs* T plot for **1d** after diamagnetic correction χ_d of -11.96×10^{-4} cm^3mol^{-1}. The horizontal red line marks the Curie value of 0.375 Kcm^3mol^{-1} for an ideal paramagnet. The green box marks the feature related to traces of molecular oxygen trapped in the holder cavity with the sample.

The feature in the range 30–50 K related to molecular oxygen was removed from further analysis.

Analysis for radical dimer **1d**

The same experimental magnetization data was analyzed as dimer of two spins. Thus, a microcrystalline sample of derivative **1d** (m = 8.53 mg, 0.882×10^{-5} mol, M_w = 967.28 g mol^{-1} per dimer) was analyzed at 0.6 T in the 300–2 K temperature range at 0.6 T. The total molar magnetic susceptibility $\chi_{tot}(T)$ and $\chi_{tot}T(T)$ plots are shown in Figures S31 and S32, respectively (*vide supra*).

Considering the discrete dimer structure of **1d** in the solid-state, the magnetic data was

analyzed using the Bleaney-Bowers model[10] (the BB model) and the BB model with a mean field approximation based on the Hamiltonian: $\mathcal{H} = -2JS_1S_2$;[11] for two 1/2 spins.

The Bleaney-Bowers model for two interacting spins is shown in eq S2.

$$\chi_{\mathrm{BB}}(T) = \frac{N_A g\mu_B}{B}\left(\frac{\sinh\left(\frac{g\mu_B B}{k_B T}\right)}{1+\exp\left(-\frac{2J}{k_B T}\right)+2\cosh\left(\frac{g\mu_B B}{k_B T}\right)}\right)$$

For low fields it reduces to: $\chi_{BB}(T) = \frac{N_A g^2\mu_B^2}{k_B T\left(3+\exp\left(-\frac{2J}{k_B T}\right)\right)}$ (eq S2)

where N_A-Avogadro number, g-electron g-factor, μ_B-Bohr magneton (in CGS units), k_B – Boltzmann constant, S – electron spin, B – magnetic field (in T), K – temperature (K), J – exchange integral.

Since $N_A g^2\mu_B^2/k_B = 1.504$ K cm^3/mol, eq S2 gives eq S3 after substitution:

$$\chi_{BB} = \frac{1.504}{T\left(3+\exp\left(-\frac{2J}{k_B T}\right)\right)} \text{ or } \chi_{BB}T = \frac{1.504}{\left(3+\exp\left(-\frac{2J}{k_B T}\right)\right)} \qquad \text{(eq S3)}$$

and with the diamagnetic component it gives eq S4:

$$\chi_{tot}T = \chi_{BB}T + \chi_{dia}T = \frac{1.504}{\left(3+\exp\left(-\frac{2J}{k_B T}\right)\right)} + \chi_{dia}T \qquad \text{(eq S4)}$$

The correction for a mean field (the zJ' model),[11] which accounts for interdimer interactions gives eq S5:

$$\chi'T = \frac{\chi T}{1-\left(\frac{zJ'}{N_A g^2\mu_B^2}\right)\chi} = \frac{\chi T}{1-\left(\frac{zJ'}{k_B}\right)\left(\frac{k_B}{N_A g^2\mu_B^2}\right)\chi} \qquad \text{(eq S5)}$$

Where χ-molar susceptibility for a given model, zJ'/k_B- interaction parameter between the nearest neighbor magnetic species in K, and $N_A g^2\mu_B^2/k_B = 1.504$ K cm^3/mol. For the Bleaney-Bowers model[10] (BB model) the mean filed approximation takes the form of equation S6:

$$\chi'T = \frac{\chi_{BB}T}{1-\left(\frac{zJ'}{1.504\times k_B}\right)\chi_{BB}} \qquad \text{(eq S6)}$$

After inclusion of the diamagnetic correction, χ_{dia}, the total magnetization χ_{tot} can be expressed as eq S7:

$$\chi'_{tot}T = \frac{\chi_{BB}T}{1-\left(\frac{zJ'}{1.504\times k_B}\right)\chi_{BB}} + \chi_{dia}T \qquad \text{(eq S7)}$$

Which gives the fitting function eq S8:

$$\chi'_{tot}T = \frac{1.504}{3+exp(\frac{-m1}{M_0})} \times \frac{1}{1-(\frac{m_2}{1.504})\times 1.504/(3+exp(\frac{-m1}{M_0}))} + m_3T \qquad \text{(eq S8)}$$

where

$m1 = 2J/k_B$

$m2 = zJ'/k_B$

$m3 = \chi_{dia}$

The total molar magnetic susceptibility data, χ_{tot}, was fitted to the pure BB model containing the diamagnetic correction, χ_{dia} (eq S4, Figure S35). Expansion of the low temperature section of the plot shows discrepancy between the data and the model. The resulting χ_{dia} was used to obtain the paramagnetic susceptibility χ_p, and the $\chi_p T$ *vs* T plot is shown in Figure S36.

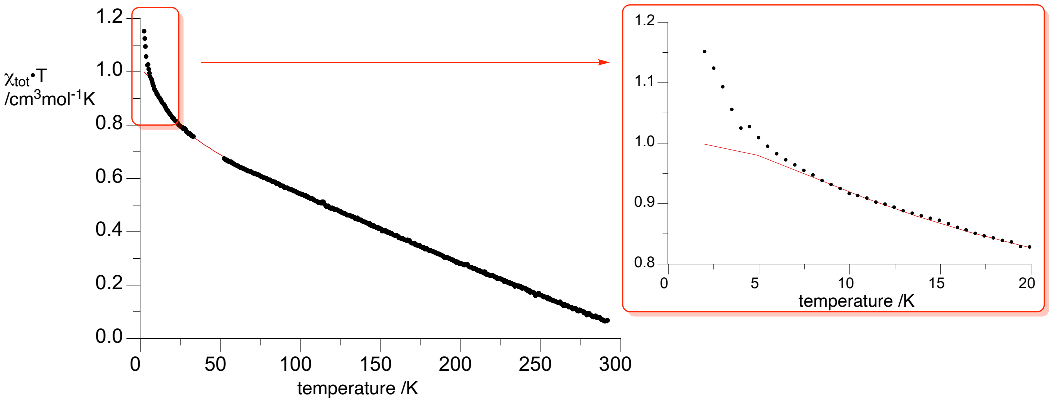

Figure S35. Left: $\chi_{tot}T$ *vs* T plot for dimeric **1d** and fitting to the Bleaney-Bowers model (eq. S4). Fitting parameters: $2J/k_B$ = 16.6(2) K and χ_{dia}=-0.002418(3) cm^3mol^{-1}; r^2 = 0.998. Right: expanded low temperature portion of the plot.

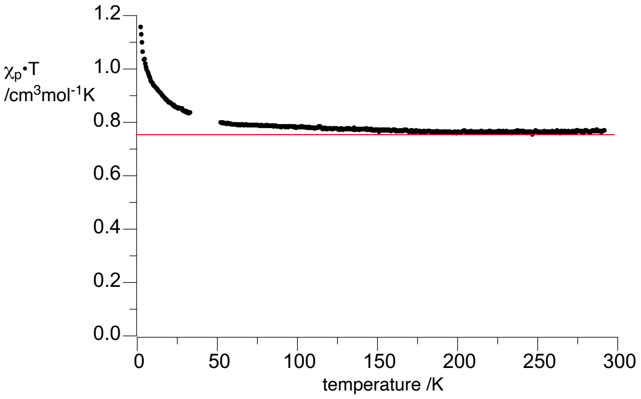

Figure S36. $\chi_p T$ *vs* T plot for dimer **1d** plot after applying the diamagnetic correction χ_{dia}= -0.002418(3) cm^3mol^{-1}. The horizontal red line marks the Curie value of 0.75 Kcm^3mol^{-1} for two ideal spins S =1/2.

Fitting the χ_{tot} data to the BB model with mean field approximation (eq S8) is shown in Figure S37.

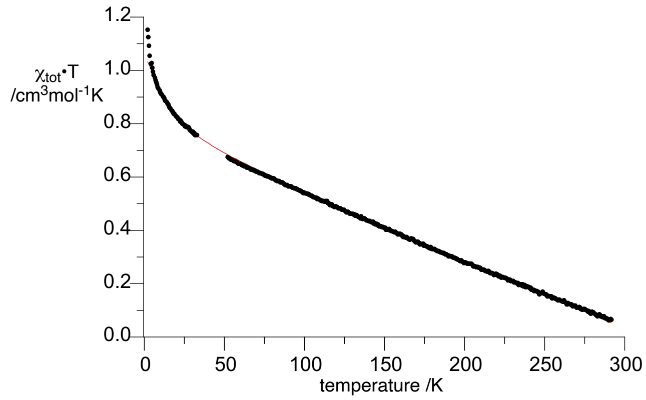

Figure S37. $\chi_{\text{tot}}T$ *vs T* plot for dimer **1d** plot and fitting to the Bleaney-Bowers model with mean field approximation (eq. S8). Fitting parameters: $2J/k_B$ = 12.0(4) K, zJ'/k_B = 0.10(1) K, and χ_{dia}=-0.002496(7) cm^{3}mol^{-1}; r^2 = 0.998.

Results demonstrate that the pure BB model describes well magnetic behavior of the analyzed solid-state sample of **1d**, which indicates that the sole exchange interaction is between molecules in the dimer and essentially negligible between the dimers above 8 K.

Magnetization *M* vs magnetic field *B* for monomeric radical **1d**

Paramagnetic susceptibility is described by the Brillouin model (the B model, eq S9):

$$\chi_p(T,B) = n_s \frac{N_A g \mu_B}{B}\left(\frac{(S+0.5)}{\tanh\left((S+0.5)g\mu_B B/k_B T\right)} - \frac{0.5}{\tanh\left(0.5 g\mu_B B/k_B T\right)}\right) \quad \text{eq S9}$$

where N_A-Avogadro number, g-electron g-factor, μ_B-Bohr magneton (in CGS units), k_B-Boltzmann constant, S-electron spin, B – magnetic field (in T), T – temperature (K), and n_s – fraction of units with spin S in 1 mol.

Combined constants give:

$\frac{g\mu_B}{k_B} = 1.3450$ K/T and $\mu_B N_A g = 11183.45$ cm^3 G/mol (or emu/mol)

Considering that $Mp = \chi_p \times B$ substitution gives eq S10:

$$Mp = n_s \times 11183.45 \times \left(\frac{(S+0.5)}{\tanh\left(\frac{(S+0.5)*1.3450*B}{T}\right)} - \frac{0.5}{\tanh\left(\frac{0.67248*B}{T}\right)}\right) \quad \text{(eq S10)}$$

For n_s to express the number of active spins in the material per 1 mol, eq S9 is divided by the number of spins in the fundamental spin unit S, which leads to eq S10:

$$Mp = \frac{n_s}{2S} \times 11183.45 \times \left(\frac{(S+0.5)}{\tanh\left(\frac{(S+0.5)*1.3450*B}{T}\right)} - \frac{0.5}{\tanh\left(\frac{0.67248*B}{T}\right)}\right) \quad \text{(eq S11)}$$

The fitting of the experimental data to equation S11 is shown in Figure S38.

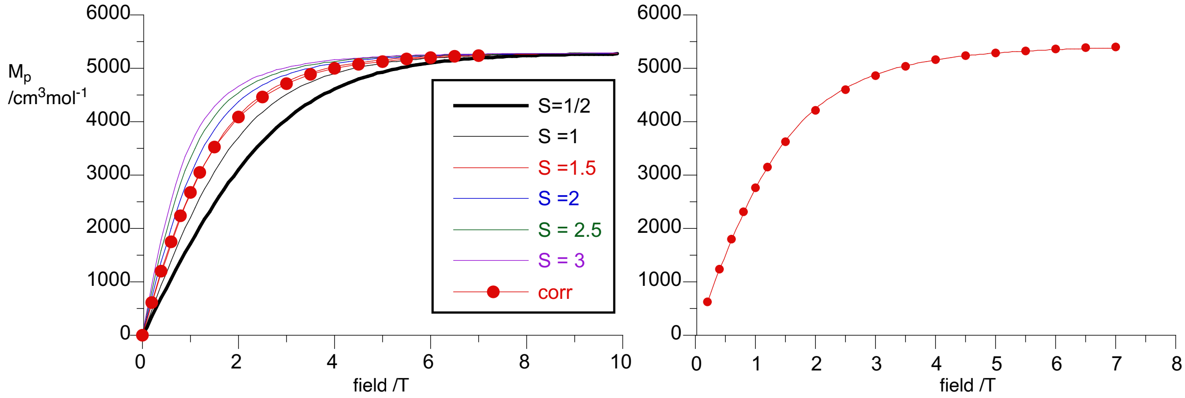

Figure S38. Left: M_p *vs B* experimental data for monoradical **1d** (red dots) and Brillouin function plots for several S values. Right: M_p *vs B* plot for monoradical **1d** (red dots). Numerical fitting to eq S11 gave $S = 1.56(1)$, $n_s = 0.968(1)$, $r^2 = 0.9999$. Measurement performed at T = 2 K.

10. Computational details and results

Quantum-mechanical calculations were carried out using Gaussian 16 suite of programs.[12] Geometry optimizations of the precursors **2** were conducted at the CAM-B3LYP/6-311G(d,p) level of theory in ethyl acetate dielectric medium (PCM model[13]) requested with the SCRF(Solvent=EthylEthanoate) keyword and using tight convergence limits and without symmetry constrains. Geometries of radicals **1** and reference radicals **A(X=H)** and **B(X=H)** were optimized at the UB3LYP/6-311G(d,p) level of theory in vacuum using tight convergence limits and without symmetry constrains.

a) mechanistic investigation of photocyclization of 2

Mechanistic investigation of photocyclization of model **2'** (in which t-Bu was approximated with Me) was conducted at the CAM-B3LYP/6-311G(d,p) level of theory in EtOAc dielectric medium (PCM model[13]) requested with the SCRF(Solvent=EthylEthanoate) keyword and tight convergence limits. Excitation calculations of **2'** were conducted using the TD-DFT method for closed-shell systems. Geometry optimization in the S_1 state was performed using Fopt with default convergent limits and TD=(singlets, root=1, NStates=3) keywords in AcOEt dielectric medium (PCM model[13]).

The triplet state geometries of precursors **2** were obtained using the UCAM-B3LYP/6-311G(d,p) method and starting with GS geometries of **2**. TD-DFT calculations for closed-shell singlet at the triplet geometries using CAM-B3LYP/6-311G(d,p) method and TD=(triplets, root=1, NStates=12) keyword gave the forbidden S_0–>T_n transitions. Partial Jablonski diagrams for **2a**–**2f** are shown in Figure S39.

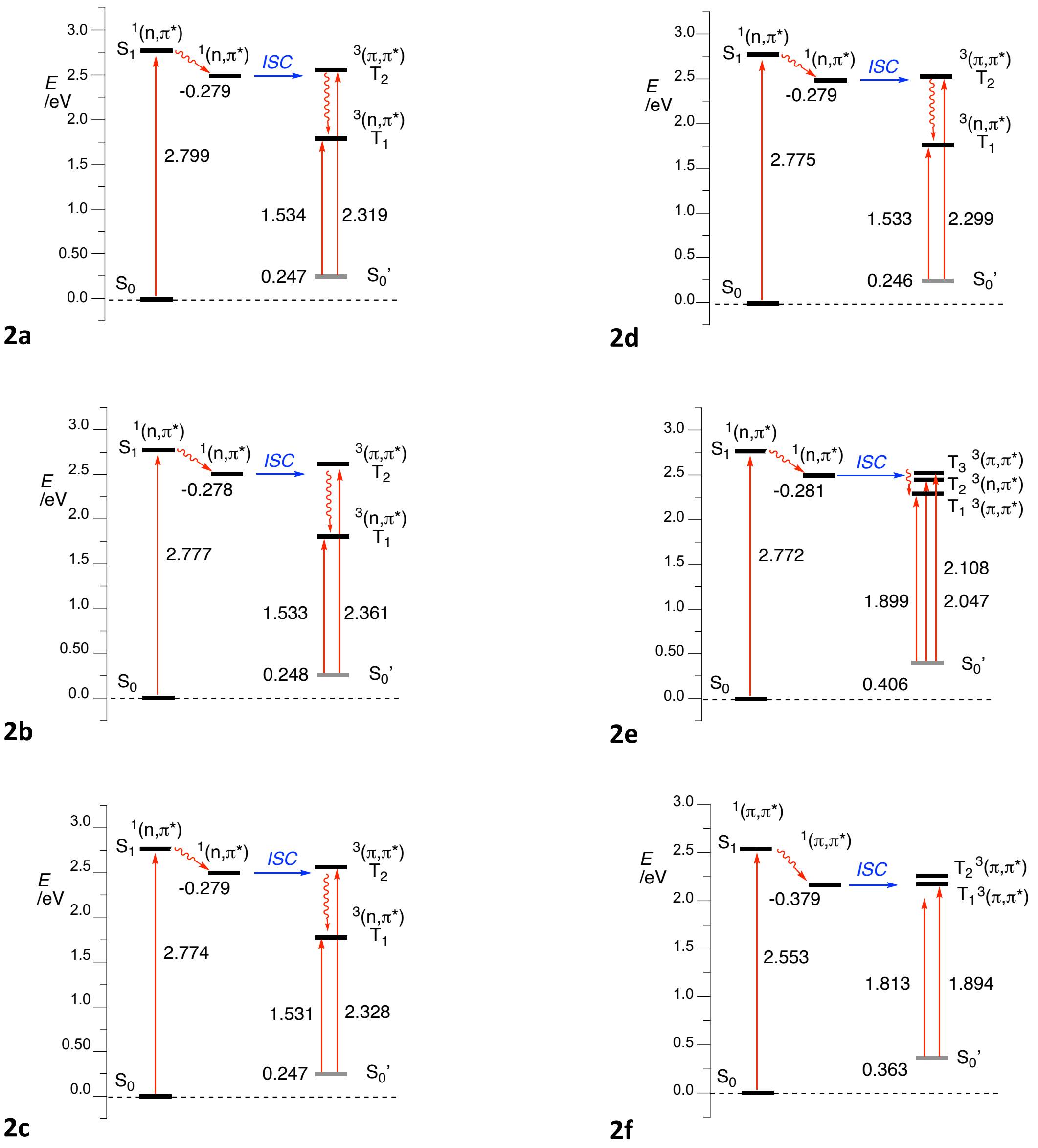

Figure S39. Partial Jablonski diagrams for precursors **2a**–**2f**.

Natural charges for radical ions $\mathbf{2}^{+\bullet}$ and $\mathbf{2}^{-\bullet}$ were obtained by population analysis (pop=NBO) of the UCAM-B3LYP/6-311G+(d,p) wavefunction obtained by single point calculation of the radical ion generated by adding or subtracting an electron from precursors **2** at the optimized GS geometry (CAM-B3LYP/6-311G(d,p) level of theory in AcOEt dielectric medium) in AcOEt dielectric medium (PCM model).[13]

b) isotropic Fermi contact coupling constants (hfcc) and spin densities for radicals

Isotropic Fermi contact coupling constants for radicals **A**, **B**, **1** and carbazole substituted reference radicals were calculated using the UCAM-B3LYP/EPR-III // UB3LYP/6-311G(d,p) method in benzene dielectric medium requested with the SCRF(Solvent=Benzene) keywords (PCM model).[13] Fermi contact parameters for **B(X=H)** were obtained with the EPR-III basis

set for all atoms expect for the sulfur atom, for which 6-311G+(2df) basis set was used implemented with the "gen" keyword. The resulting *hfcc* values are shown in Table S6 and spin densities are listed in Table S7 and S8.

Spin densities calculated for the triazine, benzo[*e*][1,2,4]triazine with the Ph ring at the N(1) position of the benzo[*e*][1,2,4]triazine ring were correlated with Hammett parameters[7] for model substituents and results are shown in Figure S40.

Table S6. DFT calculated hyperfine coupling constants (G) of radicals **1** in benzene at the UCAM-B3LYP/EPR-III // UB3LYP/6-311G(d,p) level of theory.[a]

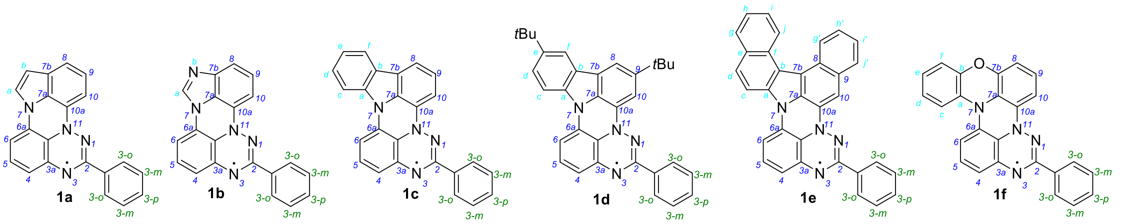

hfcc /G	**1a**	**1b**	**1c**	**1d**	**1e**	**1f**
$a_{N(1)}$	3.81	4.05	3.78	3.71	3.94	3.87
$a_{N(3)}$	4.03	4.16	4.04	4.01	4.09	4.09
$a_{N(11)}$	6.04	5.88	6.08	6.18	5.62	6.32
$a_{N(7)}$	0.07	-0.09	0.39	0.41	0.04	0.77
$a_{N(b)}$	-	0.29	-	-	-	-
$a_{H(4)}$	-0.55	-0.89	-0.43	-0.37	-0.75	-0.31
$a_{H(5)}$	-0.74	-0.53	-0.82	-0.88	-0.73	-0.89
$a_{H(6)}$	-0.84	-1.26	-0.73	-0.65	-1.12	-0.65
$a_{H(8)}$	-3.32	-3.10	-2.94	-2.92	-	-2.01
$a_{H(9)}$	1.33	1.29	1.17	-	-	1.09
$a_{H(10)}$	-3.62	-3.32	-3.15	-3.17	-4.50	-2.11
$a_{H(a)}$	0.49	-0.03	-	-	-	-
$a_{H(b)}$	-0.91	-	-	-	-	-
$a_{H(c)}$	-	-	-0.29	-0.29	-0.18	-0.29
$a_{H(d)}$	-	-	0.29	0.32	0.32	0.18
$a_{H(e)}$	-	-	-0.32	-	-	-0.29
$a_{H(f)}$	-	-	0.28	0.26	-	0.14
$a_{H(g)}$<br>$a_{H(g')}$	-	-	-	-	0.11<br>1.03	-
$a_{H(h)}$<br>$a_{H(h')}$	-	-	-	-	-0.12<br>-1.68	-
$a_{H(i)}$<br>$a_{H(i')}$	-	-	-	-	0.11<br>0.89	-
$a_{H(j)}$<br>$a_{H(j')}$	-	-	-	-	-0.10<br>-1.48	-
$a_{H(3\text{-}o)}$ avg	0.50	0.52	0.50	0.49	0.54	0.50
$a_{H(3\text{-}m)}$ avg	-0.29	-0.31	-0.30	-0.29	-0.32	-0.29
$a_{H(3\text{-}p)}$	0.41	0.44	0.41	0.40	0.46	0.42

[a] For comparative purposes, the numbering of the systems is according to radical **1a**.

Table S7. DFT calculated spin densities of radicals **1** in benzene at the UCAM-B3LYP/EPR-III//UB3LYP/6-311G(d,p) level of theory.[a]

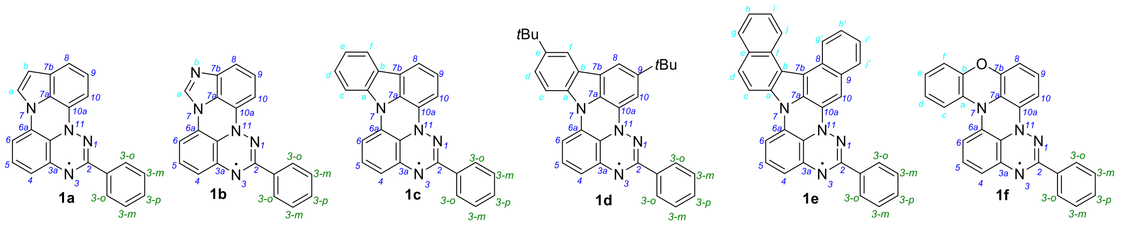

Spin density	1a	1b	1c	1d	1e	1f
$\rho_{N(1)}$	0.270	0.281	0.266	0.266	0.280	0.269
$\rho_{N(3)}$	0.271	0.280	0.272	0.268	0.275	0.275
$\rho_{N(11)}$	0.235	0.232	0.235	0.236	0.219	0.243
$\rho_{N(7)}$	0.016	0.010	0.026	0.027	0.011	0.044
$\rho_{C(2)}$	-0.058	-0.067	-0.058	-0.057	-0.062	-0.063
$\rho_{C(3a)}$	0.014	0.010	0.021	0.022	0.017	0.014
$\rho_{C(3b)}$	0.066	0.064	0.071	0.070	0.063	0.076
$\rho_{C(4)}$	0.006	0.020	0.002	-0.001	0.014	-0.002
$\rho_{C(5)}$	0.023	0.014	0.025	0.026	0.020	0.030
$\rho_{C(6)}$	0.028	0.044	0.022	0.021	0.040	0.020
$\rho_{C(6a)}$	0.022	0.014	0.019	0.021	0.014	0.027
$\rho_{C(7a)}$	0.053	0.053	0.021	0.053	0.037	0.090
$\rho_{C(7b)}$	-0.042	-0.040	-0.041	-0.028	-0.024	-0.043
$\rho_{C(8)}$	0.125	0.114	0.112	0.103	0.063	0.075
$\rho_{C(9)}$	-0.062	-0.058	-0.054	-0.049	-0.045	-0.043
$\rho_{C(10)}$	0.128	0.116	0.107	0.094	0.149	0.069
$\rho_{C(10a)}$	-0.079	-0.073	-0.069	-0.053	-0.075	-0.062
$\rho_{C(a)}$	-0.022	-0.001	-0.011	-0.013	-0.013	-0.009
$\rho_{X\,(b)}$	0.037	0.019	0.014	0.015	0.015	0.012
$\rho_{C(c)}$	-	-	0.013	0.012	0.010	0.011
$\rho_{C(d)}$	-	-	-0.011	-0.012	-0.011	-0.007
$\rho_{C(e)}$	-	-	0.013	0.012	0.005	0.010
$\rho_{C(f)}$	-	-	-0.008	-0.007	-0.006	-0.008
$\rho_{C(g)}$<br>$\rho_{C(g')}$	-	-	-	-	-0.004<br>-0.047	-
$\rho_{C(h)}$<br>$\rho_{C(h')}$	-	-	-	-	0.004<br>-0.036	-
$\rho_{C(i)}$<br>$\rho_{C(i')}$	-	-	-	-	-0.004<br>0.064	-
$\rho_{C(j)}$<br>$\rho_{C(j')}$	-	-	-	-	0.004<br>0.057	-
$\rho_{C(3\text{-}ipso)}$	0.005	0.006	0.003	0.003	0.006	0.020
$\rho_{C(3\text{-}o)}$ avg	-0.021	-0.022	-0.020	-0.020	-0.022	-0.022
$\rho_{C(3\text{-}m)}$ avg	0.011	0.012	0.012	0.012	0.013	0.011
$\rho_{C(3\text{-}p)}$	-0.017	-0.018	-0.017	-0.016	-0.018	-0.017

[a] For comparative purposes, the numbering of the systems is according to radical **1a**.

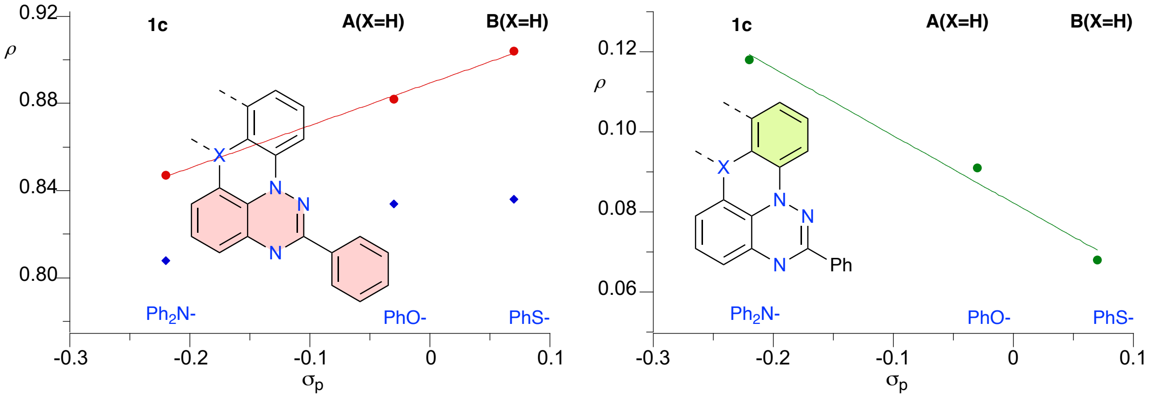

Figure S40. Left: Correlation of DFT spin densities for the triazine ring (blue diamonds) and benzo[*e*][1,2,4]triazine containing the phenyl ring (red) with Hammett parameter σ_p for model substituents (blue). Best fitting function: $\rho = 0.195(9)\times\sigma_p + 0.889(1)$, $r^2 = 0.998$.
Right: A similar correlation of DFT spin density on the N-Ph ring (green) with σ_p. Best fitting function: $\rho = -0.17(2)\times\sigma_p + 0.082(3)$, $r^2 = 0.983$.

Table S8. DFT calculated spin densities of substituted carbazole radicals in benzene at the UCAM-B3LYP/EPR-III//UB3LYP/6-311G(d,p) level of theory.[a]

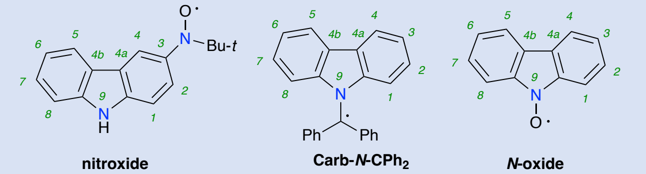

Spin density	nitroxide	carb-*N*-CPh₂	*N*-oxide
$\rho_{C(1)}$	-0.031	0.009	0.103
$\rho_{C(2)}$	0.053	-0.005	-0.048
$\rho_{C(3)}$	-0.040	0.009	0.095
$\rho_{C(4)}$	0.063	-0.006	-0.048
$\rho_{C(4a)}$	-0.022	0.009	0.083
$\rho_{C(4b)}$	0.005	0.009	0.083
$\rho_{C(5)}$	-0.005	-0.006	-0.048
$\rho_{C(6)}$	0.004	0.009	0.095
$\rho_{C(7)}$	-0.005	-0.005	-0.048
$\rho_{C(8)}$	0.005	0.009	0.103
$\rho_{C(8a)}$	-0.004	0.011	-0.077
$\rho_{N(9)}$	0.005	0.004	0.287
$\rho_{C(9a)}$	0.039	0.011	-0.077
carbazole total	0.066	0.061	0.505
ρ_N	0.418	–	–
ρ_O	0.507	–	0.495
ρ_{tBu}	0.009	–	–
ρ_C	–	0.592	–
ρ_{Ph}x2	–	0.347	–

c) spin delocalization in radicals in benzene dielectric medium

Spin delocalization parameter RDV (Radical Delocalization Value)[14] was calculated according to the formula (eq S12):

$$RDV = \sum_{i=1}^{n}(\rho_i)^2 \qquad \text{eq S12}$$

where spin concentration ρ_i on heavy atoms i (hydrogen atoms summed up to heavy atoms) is obtained with the UCAM-B3LYP/EPR-III // UB3LYP/6-311G(d,p) method in benzene dielectric mediums using the PCM model.[13] For the purpose of this work, an inverse is reported: RDV^{-1} = 1/RDV, since now larger values corresponds to greater delocalization. Results are shown in Table S9 and in Figure S41. Figure S42 shows correlations RDV^{-1} with Hammett parameters for model substituents.

Table S9. Radical delocalization value (RDV^{-1}) for radicals **1**.

Radical	RDV^{-1} (in benzene)
**A (X=H)**	3.843
**B (X=H)**	3.591
**1a**	3.826
**1b**	3.782
**1c**	3.988
**1d**	4.173
**1e**	3.822
**1f**	4.017

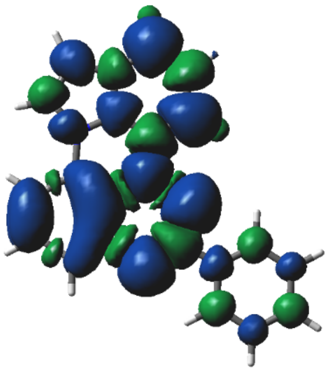

1a

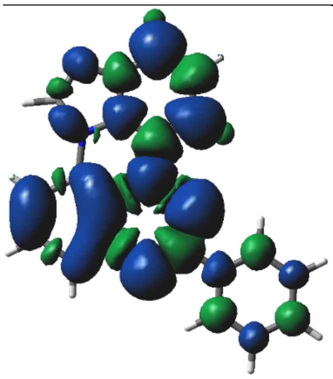

1b

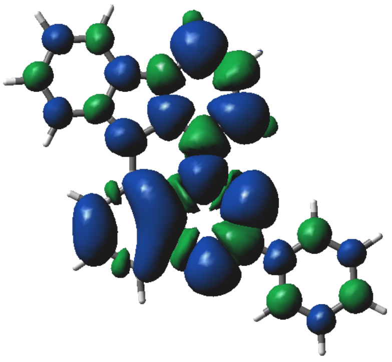

1c

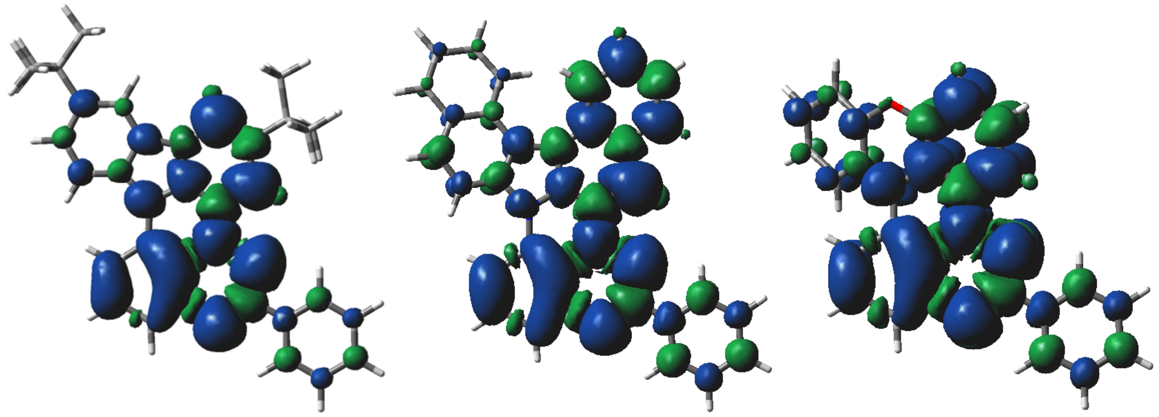

1d **1e** **1f**

Figure S41. Spin densities of radicals **1** at the UCAM-B3LYP/EPR-III // UB3LYP/6-311G(d,p) level of theory in benzene dielectric medium. Isovalue: MO = 0.020, Density = 0.0004.

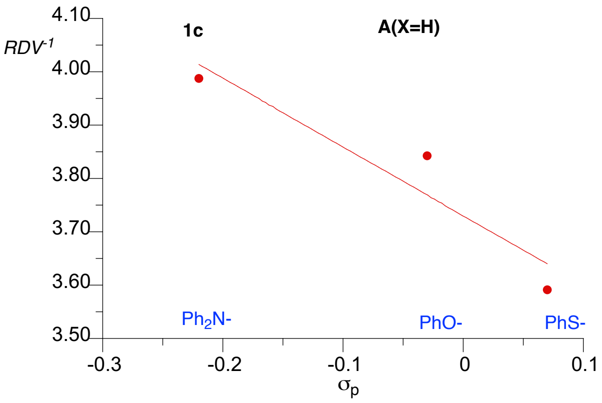

Figure S42. Correlation of RDV^{-1} with Hammett parameters σ_p for model substituents (blue). Best fitting functions: $a_{N1} = -1.3(4) \times \sigma_p + 3.73(6)$, $r^2 = 0.89$.

d) electronic excitations

Electronic excitation energies in CH_2Cl_2 dielectric medium were obtained at the UB3LYP/6-311++G(d,p) // UB3LYP/6-311G(d,p) level of theory using the time-dependent TD-DFT method[15] supplied in the Gaussian 16 package. Solvation models in calculations were implemented with the PCM model[13] using the SCRF(solvent=CH_2Cl_2) keyword. Three lowest excitation energies, classified as $\pi \rightarrow \pi^*$ transitions are listed in Table S10.

Energies of FMOs involved in the low energy transitions are listed in Table S11. The calculated energies of HOMO and LUMO orbitals together with their visualizations are shown in Figure 43.

Table S10. Electronic transition energies and oscillator strength values with the indicated main electronic transition obtained at the TD-UB3LYP/6-311++G(d,p) // UB3LYP/6-311G(d,p) level of theory in CH_2Cl_2 dielectric medium.

Radical	π→π* β-HOMO→β-LUMO /nm (*f*)	π→π* α-HOMO→α-LUMO /nm (*f*)	π→π* β-HOMO-1→β-LUMO /nm (*f*)	π→π* α-HOMO→α-LUMO+1 /nm (*f*)
**A(X=H)**	586.6 (0.053)	614.8 (0.002)	389.7 (0.040)	452.5 (0.041)
**1a**	679.2 (0.080)	614.0 (0.005)	476.6 (0.003)	453.0 (0.008)
**1b**	627.7 (0.067)	597.8 (0.015)	447.7 (0.004)	453.6 (0.010)
**1c**	662.4 (0.084)	626.6 (0.007)	465.4 (0.019)	494.4 (0.047)
**1d**	679.0 (0.099)	632.8 (0.007)	504.5 (0.016)	440.4 (0.021)
**1e**	707.8 (0.063)	605.5 (0.014)	638.6 (0.044)	499.7 (0.116)
**1f**	754.1 (0.073)	621.2 (0.004)	468.0 (0.025)	488.8 (0.011)

Table S11. Energies of MO involved in low energy transitions obtained from the UB3LYP/6-311++G(d,p) // UB3LYP/6-311G(d,p) method in CH_2Cl_2 dielectric medium.

Radical	α-HOMO π /eV	α-LUMO π* /eV	α-LUMO+1 π* /eV	β-HOMO-1 π /eV	β-HOMO π* /eV	β-LUMO π* /eV
**A(X=H)**	-4.933	-1.870	-1.195	-6.975	-6.095	-3.066
**B(X=H)**	-5.019	-1.850	-1.337	-6.874	-5.953	-3.170
**1a**	-4.866	-1.834	-1.182	-6.598	-5.782	-3.129
**1b**	-5.036	-1.929	-1.398	-6.927	-6.112	-3.276
**1c**	-4.823	-1.840	-1.466	-6.487	-5.771	-3.090
**1d**	-4.762	-1.796	-1.341	-6.299	-5.660	-3.048
**1e**	-4.939	-1.927	-1.784	-5.908	-5.741	-3.250
**1f**	-4.806	-1.804	-1.306	-6.513	-5.461	-3.032

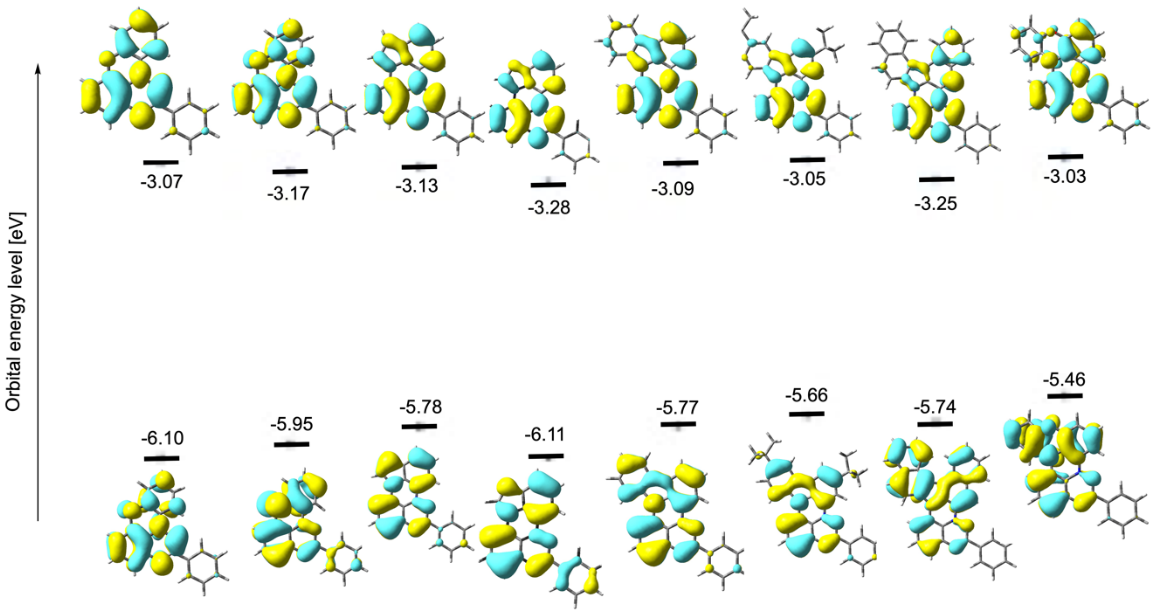

Figure S43. The contours and energies of β-HOMO (lower) and β-LUMO (upper) orbitals calculated at the B3LYP/6-311++G(d,p)//B3LYP/6-311G(d,p) level of theory in CH_2Cl_2 dielectric medium.

e) partial output data from TD-DFT calculations for radicals 1

Method: UB3LYP/6-311++G(d,p)// UB3LYP/6-311G(d,p)

Keywords: TD(nstates=20, root=1) SCRF(solvent=CH2CL2) SCF=tight

A(X=H)

```
Excited State   1: 2.048-A'  2.0167 eV  614.79 nm  f=0.0019  <S**2>=0.799
     78A -> 79A         0.94989
     76B -> 78B        -0.10713
     77B -> 78B        -0.20245
 This state for optimization and/or second-order correction.
 Total Energy, E(TD-HF/TD-DFT) =  -970.866917142
 Copying the excited state density for this state as the 1-particle RhoCI
density.

 Excited State   2:  2.078-A'     2.1136 eV  586.60 nm  f=0.0529  <S**2>=0.830
     78A -> 79A         0.19398
     78A -> 80A        -0.10304
     77B -> 78B         0.95413

 Excited State   3:  2.191-A'     2.7399 eV  452.51 nm  f=0.0407  <S**2>=0.950
     77A -> 80A         0.12804
     78A -> 80A         0.94501
     72B -> 78B         0.13306
     77B -> 80B        -0.10501

 Excited State   4:  2.047-A"     2.9565 eV  419.35 nm  f=0.0011  <S**2>=0.798
     73B -> 78B         0.98747

 Excited State   5:  2.397-A'     3.1101 eV  398.66 nm  f=0.0398  <S**2>=1.186
     76A -> 79A         0.12731
```

```
     77A -> 79A        0.29700
     78A -> 79A        0.12701
     76B -> 78B        0.87466
     76B -> 79B       -0.12656
     77B -> 79B       -0.17488
```

1a

```
Excited State   1:  2.253-A' 2.0194 eV  613.96 nm  f=0.1017  <S**2>=1.019
     83A -> 86A       -0.11775
     84A -> 87A       -0.11051
     83B -> 84B        0.94510
 This state for optimization and/or second-order correction.
 Total Energy, E(TD-HF/TD-DFT) =  -1026.50058751
 Copying the excited state density for this state as the 1-particle RhoCI density.

 Excited State   2:  2.166-A'     2.3691 eV  523.34 nm  f=0.0052  <S**2>=0.923
     83A -> 85A        0.26477
     84A -> 85A        0.85893
     84A -> 87A        0.18849
     84A -> 95A        0.15933
     81B -> 84B        0.10106

 Excited State   3:  2.767-A'     2.9184 eV  424.84 nm  f=0.0192  <S**2>=1.664
     81A -> 85A       -0.11226
     82A -> 86A        0.16170
     82A -> 87A       -0.10915
     83A -> 87A       -0.20464
     84A -> 85A       -0.20513
     84A -> 86A        0.12892
     84A -> 87A        0.45994
     84A -> 95A       -0.13869
     79B -> 84B        0.18215
     81B -> 84B       -0.10938
     81B -> 85B        0.12765
     82B -> 84B        0.55750
     82B -> 88B        0.16317
     83B -> 86B       -0.21225
     83B -> 88B       -0.18785

 Excited State   4:  2.362-A'     3.1631 eV  391.97 nm  f=0.0031  <S**2>=1.145
     84A -> 86A        0.72851
     84A -> 87A        0.31048
     82B -> 84B       -0.46102
     83B -> 85B       -0.13668
     83B -> 86B       -0.10248
```

1b

```
Excited State   1:  2.109-A'   1.9751 eV  627.72 nm  f=0.0666  <S**2>=0.862
     84A -> 85A       -0.20493
     84A -> 87A       -0.13510
     83B -> 84B        0.94996
 This state for optimization and/or second-order correction.
 Total Energy, E(TD-HF/TD-DFT) =  -1043.28180792
 Copying the excited state density for this state as the 1-particle RhoCI density.

 Excited State   2:  2.035-A'     2.0742 eV  597.76 nm  f=0.0152  <S**2>=0.785
```

```
     84A -> 85A         0.94166
     84A -> 87A        -0.11511
     81B -> 84B        -0.10339
     83B -> 84B         0.19847

 Excited State   3:  2.089-A'     2.7342 eV  453.46 nm  f=0.0099  <S**2>=0.841
     84A -> 86A         0.92726
     84A -> 87A         0.25105
     82B -> 84B        -0.19683

 Excited State   4:  2.230-A'     2.7696 eV  447.66 nm  f=0.0038  <S**2>=0.993
     82A -> 87A        -0.10477
     84A -> 85A         0.10309
     84A -> 87A         0.39042
     81B -> 84B        -0.22890
     82B -> 84B         0.82190

 Excited State   5:  2.045-A"     2.8632 eV  433.02 nm  f=0.0011  <S**2>=0.795
     79B -> 84B         0.98300
     79B -> 87B        -0.10458

 Excited State   6:  2.220-A'     2.9983 eV  413.52 nm  f=0.0320  <S**2>=0.982
     83A -> 85A         0.12461
     84A -> 85A         0.13074
     84A -> 86A        -0.10269
     84A -> 87A         0.47322
     78B -> 84B         0.13388
     81B -> 84B         0.79117

 Excited State   7:  2.157-A'     3.0221 eV  410.26 nm  f=0.1628  <S**2>=0.913
     83A -> 86A         0.11670
     84A -> 86A        -0.27813
     84A -> 87A         0.56728
     78B -> 84B         0.22875
     81B -> 84B        -0.48728
     82B -> 84B        -0.45850
     83B -> 85B        -0.12236
     83B -> 86B        -0.10159
```

1c

```
Excited State   1:  2.078-A   1.8719 eV  662.35 nm  f=0.0835  <S**2>=0.830
     97A -> 99A        -0.13791
     96B -> 97B         0.97392
 This state for optimization and/or second-order correction.
 Total Energy, E(TD-HF/TD-DFT) =  -1180.91934695
 Copying the excited state density for this state as the 1-particle RhoCI density.

 Excited State   2:  2.040-A      1.9788 eV  626.57 nm  f=0.0072  <S**2>=0.790
     96A -> 98A        -0.12894
     97A -> 98A         0.95847
     97A -> 99A        -0.12347
     94B -> 97B        -0.11344

 Excited State   3:  2.219-A      2.5080 eV  494.36 nm  f=0.0472  <S**2>=0.981
     97A -> 98A         0.11540
     97A -> 99A         0.70330
     97A ->100A         0.24740
     95B -> 97B        -0.57360
     96B -> 97B         0.11355
```

```
 Excited State   4:  2.136-A      2.6639 eV  465.42 nm  f=0.0190  <S**2>=0.890
     97A -> 99A        0.24337
     97A ->100A        0.70774
     95B -> 97B        0.61888

 Excited State   5:  2.251-A      2.7581 eV  449.53 nm  f=0.0483  <S**2>=1.016
     95A -> 99A        0.11688
     97A -> 99A       -0.56112
     97A ->100A        0.60484
     95B -> 97B       -0.44774
     96B -> 98B        0.15889

 Excited State   6:  2.044-A      2.9473 eV  420.68 nm  f=0.0011  <S**2>=0.795
     92B -> 97B        0.98139

 Excited State   7:  2.223-A      2.9905 eV  414.59 nm  f=0.0630  <S**2>=0.986
     94A -> 98A        0.10939
     96A -> 98A        0.23972
     97A -> 98A        0.13365
     97A -> 99A       -0.10856
     94B -> 97B        0.90862
     94B -> 98B       -0.10142
```

1d

```
Excited State   1:  2.074-A   1.8260 eV  678.98 nm  f=0.0987  <S**2>=0.825
    129A -> 131A       0.12438
    128B -> 129B       0.97648
 This state for optimization and/or second-order correction.
 Total Energy, E(TD-HF/TD-DFT) =  -1495.51217571
 Copying the excited state density for this state as the 1-particle RhoCI
density.

 Excited State   2:  2.040-A      1.9593 eV  632.79 nm  f=0.0069  <S**2>=0.791
    128A -> 130A      -0.14351
    129A -> 130A       0.96025
    126B -> 129B      -0.10457

 Excited State   3:  2.189-A      2.4578 eV  504.45 nm  f=0.0161  <S**2>=0.948
    127A -> 131A       0.13381
    129A -> 131A       0.39244
    127B -> 129B       0.87195
    127B -> 131B      -0.10107

 Excited State   4:  2.188-A      2.6519 eV  467.53 nm  f=0.0430  <S**2>=0.946
    129A -> 130A       0.10897
    129A -> 131A      -0.58068
    129A -> 132A       0.66865
    122B -> 129B      -0.11005
    127B -> 129B       0.35754
    128B -> 131B       0.10689

 Excited State   5:  2.242-A      2.7575 eV  449.63 nm  f=0.0531  <S**2>=1.007
    128A -> 131A       0.10505
    129A -> 131A       0.60358
    129A -> 132A       0.68232
    126B -> 129B      -0.11020
    127B -> 129B      -0.20748
    128B -> 130B       0.19854
```

1e

```
Excited State   1:  2.143-A 1.7516 eV  707.84 nm  f=0.0634  <S**2>=0.899
    123A ->124A        0.16408
    123A ->125A       -0.13404
    120B ->123B       -0.11867
    121B ->123B       -0.17298
    122B ->123B        0.92740
 This state for optimization and/or second-order correction.
 Total Energy, E(TD-HF/TD-DFT) =  -1488.27105042
 Copying  the  excited  state  density  for  this  state  as  the  1-particle  RhoCI
density.

 Excited State   2:  2.141-A      1.9416 eV  638.56 nm  f=0.0438  <S**2>=0.896
    121B ->123B        0.96373
    122B ->123B        0.15525

 Excited State   3:  2.042-A      2.0478 eV  605.46 nm  f=0.0141  <S**2>=0.793
    123A ->124A        0.85894
    123A ->125A        0.41401
    119B ->123B        0.10439

 Excited State   4:  2.239-A      2.4811 eV  499.72 nm  f=0.1163  <S**2>=1.003
    122A ->125A        0.12254
    123A ->124A       -0.38969
    123A ->125A        0.79567
    123A ->126A       -0.13064
    123A ->127A        0.10499
    119B ->123B        0.11090
    120B ->123B        0.14770
    121B ->124B        0.10370
    122B ->123B        0.19988

 Excited State   5:  3.211-A      2.6444 eV  468.85 nm  f=0.0234  <S**2>=2.328
    120A ->125A        0.13014
    122A ->124A       -0.31029
    122A ->125A        0.44240
    122A ->126A        0.14443
    123A ->124A        0.14201
    123A ->125A       -0.18742
    120B ->123B        0.35574
    120B ->125B        0.15802
    121B ->125B        0.17234
    122B ->123B       -0.13281
    122B ->124B        0.24811
    122B ->125B        0.45589
```

1f

```
Excited State 1:  2.063-A 1.6443 eV  754.05 nm  f=0.0725  <S**2>=0.814
    100B ->101B        0.98432
 This state for optimization and/or second-order correction.
 Total Energy, E(TD-HF/TD-DFT) =  -1256.14705961
 Copying  the  excited  state  density  for  this  state  as  the  1-particle  RhoCI
density.

 Excited State   2:  2.043-A      1.9957 eV  621.25 nm  f=0.0037  <S**2>=0.793
    100A ->102A        0.15877
    101A ->102A        0.95124
    101A ->103A        0.11748

 Excited State   3:  2.196-A      2.5366 eV  488.78 nm  f=0.0105  <S**2>=0.956
    101A ->102A       -0.11604
    101A ->103A        0.93773
```

```
    101A ->104A       -0.13897
    101A ->105A        0.12040
     99B ->101B        0.11137

 Excited State   4:  2.208-A      2.6493 eV  467.98 nm  f=0.0246  <S**2>=0.969
    100A ->102A       -0.13768
    100A ->103A        0.11633
    101A ->102A        0.12778
    101A ->103A       -0.10219
     95B ->101B       -0.12006
     99B ->101B        0.92079
    100B ->102B       -0.11260

 Excited State   5:  3.117-A      2.8509 eV  434.89 nm  f=0.0153  <S**2>=2.179
    100A ->102A        0.43767
    100A ->104A       -0.20391
    101A ->102A       -0.12060
    101A ->104A        0.51509
     99B ->101B        0.18848
    100B ->102B        0.54778
    100B ->104B       -0.21668
```

Mechanistic studies

CAM-B3LYP/6-311G(d,p)//CAM-B3LYP/6-311G(d,p), SCRF(Solvent=EthylEthanoate)

2a,

```
Excited State   1: Singlet-A  2.7791 eV  446.13 nm  f=0.0031  <S**2>=0.000
      81 -> 85         0.65592
      81 -> 90         0.10214
      82 -> 85         0.11243
      83 -> 85         0.12334
 This state for optimization and/or second-order correction.
 Total Energy, E(TD-HF/TD-DFT) =  -1027.23335250
 Copying the excited state density for this state as the 1-particle RhoCI
density.

 Excited State   2:  Singlet-A  3.3572 eV  369.31 nm  f=0.0651
<S**2>=0.000
      79 -> 85        -0.11450
      83 -> 85        -0.14677
      84 -> 85         0.66657

 Excited State   3: Singlet-A  3.9529 eV  313.65 nm  f=0.0150  <S**2>=0.000
      82 -> 85        -0.19600
      83 -> 85         0.63391
      84 -> 85         0.17816

 Excited State   4: Singlet-A   4.0176 eV  308.60 nm  f=0.1545
<S**2>=0.000
      77 -> 85        -0.14536
      79 -> 86         0.10852
      81 -> 85        -0.13847
      82 -> 85         0.61395
      83 -> 85         0.19123
```

2a, S1 opt

```
Excited State   1: Singlet-A  2.1744 eV  570.21 nm  f=0.0026  <S**2>=0.000
      82 -> 85         0.55412
      82 -> 90         0.10724
      83 -> 85         0.38444
 This state for optimization and/or second-order correction.
 Total Energy, E(TD-HF/TD-DFT) =  -1027.24358869
 Copying the excited state density for this state as the 1-particle RhoCI
density.
```

```
 Excited State   2: Singlet-A  3.3270 eV  372.66 nm  f=0.0855  <S**2>=0.000
      79 -> 85        -0.11205
      82 -> 85         0.11472
      83 -> 85        -0.10173
      84 -> 85         0.66215

 Excited State   3: Singlet-A  3.6059 eV  343.83 nm  f=0.0006  <S**2>=0.000
      82 -> 85         0.10111
      82 -> 86         0.55280
      83 -> 86         0.38464
```

2a, at T geom

```
Excited State   1: Triplet-A  1.5340 eV  808.26 nm  f=0.0000  <S**2>=2.000
      82 -> 85         0.59010
      82 -> 86        -0.15730
      82 -> 90         0.15169
      83 -> 85        -0.26020
 This state for optimization and/or second-order correction.
 Total Energy, E(TD-HF/TD-DFT) =  -1027.27003263
 Copying the excited state density for this state as the 1-particle RhoCI
density.

 Excited State   2: Triplet-A  2.3185 eV  534.75 nm  f=0.0000  <S**2>=2.000
      78 -> 85         0.13654
      79 -> 85        -0.28365
      79 -> 86         0.11744
      81 -> 85         0.43756
      81 -> 86         0.13353
      82 -> 85         0.11613
      83 -> 85         0.14880
      84 -> 85         0.29719

 Excited State   3: Triplet-A  3.1418 eV  394.63 nm  f=0.0000  <S**2>=2.000
      79 -> 85         0.28118
      80 -> 88        -0.12424
      80 -> 89         0.15578
      81 -> 85         0.34816
      81 -> 86         0.23957
      81 -> 88         0.10548
      83 -> 85        -0.10687
      84 -> 85        -0.29857
```

2b,

```
Excited State   1:  Singlet-A  2.7770 eV  446.46 nm  f=0.0031  <S**2>=0.000
      81 -> 85         0.65692
      83 -> 85         0.11846
 This state for optimization and/or second-order correction.
 Total Energy, E(TD-HF/TD-DFT) =  -1043.29067015
 Copying the excited state density for this state as the 1-particle RhoCI
density.

 Excited State   2:  Singlet-A  3.7814 eV  327.88 nm  f=0.0710
<S**2>=0.000
      79 -> 85        -0.16037
      82 -> 85         0.23504
      83 -> 85        -0.15080
      84 -> 85         0.60724

 Excited State   3:  Singlet-A  4.0396 eV  306.92 nm  f=0.1865
<S**2>=0.000
```

```
      77 -> 85        -0.13460
      81 -> 85        -0.13609
      82 -> 85         0.60096
      83 -> 85         0.16056
      84 -> 85        -0.17431
```

2b, S1 opt

```
Excited State   1: Singlet-A   2.1737 eV  570.39 nm  f=0.0027  <S**2>=0.000
      82 -> 85         0.31135
      83 -> 85         0.42143
      84 -> 85         0.42622
 This state for optimization and/or second-order correction.
 Total Energy, E(TD-HF/TD-DFT) =  -1043.30089767
 Copying the excited state density for this state as the 1-particle RhoCI
density.

 Excited State   2:  Singlet-A  3.6127 eV  343.19 nm  f=0.0008
<S**2>=0.000
      82 -> 86        -0.31176
      83 -> 86        -0.42243
      84 -> 86        -0.42455

 Excited State   3: Singlet-A   3.7347 eV  331.98 nm  f=0.1300
<S**2>=0.000
      79 -> 85        -0.14589
      81 -> 85         0.24618
      82 -> 85         0.26817
      83 -> 85         0.31851
      84 -> 85        -0.46884
```

2b, at T geom

```
Excited State   1: Triplet-A  1.5333 eV  808.64 nm  f=0.0000  <S**2>=2.000
      82 -> 85         0.45417
      82 -> 86        -0.11708
      82 -> 90         0.11380
      83 -> 85         0.38490
      84 -> 85         0.24908
 This state for optimization and/or second-order correction.
 Total Energy, E(TD-HF/TD-DFT) =  -1043.32728476
 Copying the excited state density for this state as the 1-particle RhoCI
density.

 Excited State   2: Triplet-A  2.3607 eV  525.19 nm  f=0.0000  <S**2>=2.000
      78 -> 85        -0.10217
      79 -> 85        -0.30953
      79 -> 86         0.12052
      81 -> 85         0.43316
      81 -> 86         0.14984
      82 -> 85         0.17249
      84 -> 85        -0.27613

 Excited State   3:  Triplet-A 3.2031 eV  387.08 nm  f=0.0000  <S**2>=2.000
      77 -> 86         0.13253
      79 -> 85         0.36871
      80 -> 88        -0.11763
      80 -> 89         0.18988
      81 -> 85         0.31660
      81 -> 86         0.24175
      81 -> 88         0.14491
      84 -> 85         0.16193
```

2c,

```
Excited State   1: Singlet-A  2.7739 eV  446.96 nm  f=0.0027  <S**2>=0.000
      92 -> 98         0.14719
      94 -> 98         0.65629
 This state for optimization and/or second-order correction.
 Total Energy, E(TD-HF/TD-DFT) =  -1180.83162975
 Copying the excited state density for this state as the 1-particle RhoCI
density.

 Excited State   2: Singlet-A   3.2178 eV  385.30 nm  f=0.0385
<S**2>=0.000
      97 -> 98         0.68592

 Excited State   3:  Singlet-A  3.9275 eV  315.69 nm  f=0.0242
<S**2>=0.000
      95 -> 98         0.25948
      96 -> 98         0.63835

 Excited State   4: Singlet-A  4.0002 eV  309.94 nm  f=0.1335  <S**2>=0.000
      89 -> 98        -0.13149
      91 -> 99        -0.11177
      95 -> 98         0.60076
      96 -> 98        -0.27905
```

2c, S1 opt

```
Excited State   1:  Singlet-A   2.1686 eV  571.73 nm  f=0.0025
<S**2>=0.000
      95 -> 98         0.66897
      95 -> 99        -0.11518
      95 ->104         0.12679
 This state for optimization and/or second-order correction.
 Total Energy, E(TD-HF/TD-DFT) =  -1180.84188883
 Copying the excited state density for this state as the 1-particle RhoCI
density.

 Excited State   2:  Singlet-A 3.1945 eV  388.11 nm  f=0.0528  <S**2>=0.000
      97 -> 98         0.68040

 Excited State   3: Singlet-A  3.6105 eV  343.40 nm  f=0.0005  <S**2>=0.000
      95 -> 98         0.11841
      95 -> 99         0.66777
      95 ->113         0.12332
```

2c, at T geom

```
Excited State   1: Triplet-A  1.5306 eV  810.03 nm  f=0.0000  <S**2>=2.000
      95 -> 98         0.64180
      95 -> 99        -0.16943
      95 ->104         0.16479
 This state for optimization and/or second-order correction.
 Total Energy, E(TD-HF/TD-DFT) =  -1180.86825041
 Copying the excited state density for this state as the 1-particle RhoCI
density.

 Excited State   2:  Triplet-A  2.3276 eV  532.67 nm  f=0.0000
<S**2>=2.000
      91 -> 98        -0.33464
      91 -> 99         0.12478
      91 ->101         0.10886
      94 -> 98         0.45876
      94 -> 99         0.13826
      97 -> 98         0.29601
```

```
 Excited State   3: Triplet-A   3.1072 eV  399.03 nm  f=0.0000
<S**2>=2.000
      91 -> 98        -0.21829
      92 ->101         0.10225
      92 ->102        -0.14168
      94 -> 98        -0.33338
      94 -> 99        -0.21054
      94 ->101        -0.12006
      97 -> 98         0.41138
```

2d,

```
Excited State   1:  Singlet-A  2.7749 eV  446.80 nm  f=0.0026  <S**2>=0.000
     101 ->106        -0.35156
     102 ->106         0.56694
     103 ->106         0.10597
 This state for optimization and/or second-order correction.
 Total Energy, E(TD-HF/TD-DFT) =  -1259.43484969
 Copying the excited state density for this state as the 1-particle RhoCI
density.

 Excited State   2: Singlet-A  3.0766 eV  402.99 nm  f=0.0489  <S**2>=0.000
     105 ->106         0.68465

 Excited State   3:  Singlet-A  3.7960 eV  326.62 nm  f=0.0042
<S**2>=0.000
     104 ->106         0.68788

 Excited State   4: Singlet-A   3.9908 eV  310.67 nm  f=0.1344
<S**2>=0.000
      97 ->106        -0.14195
      99 ->107        -0.12465
     102 ->106        -0.13187
     103 ->106         0.62717
     104 ->106        -0.10571
     105 ->107         0.13349
```

2d, S1 opt

```
Excited State   1:  Singlet-A   2.1712 eV  571.05 nm  f=0.0024
<S**2>=0.000
     103 ->106         0.66641
     103 ->107         0.11578
     103 ->112        -0.11960
 This state for optimization and/or second-order correction.
 Total Energy, E(TD-HF/TD-DFT) =  -1259.44509320
 Copying the excited state density for this state as the 1-particle RhoCI
density.

 Excited State   2:  Singlet-A   3.0515 eV  406.30 nm  f=0.0648
<S**2>=0.000
     105 ->106         0.67983

 Excited State   3:  Singlet-A   3.6080 eV  343.64 nm  f=0.0005
<S**2>=0.000
     103 ->106         0.11873
     103 ->107        -0.66486
```

2d, at T geom

```
Excited State   1:  Triplet-A   1.5325 eV  809.04 nm  f=0.0000
<S**2>=2.000
     103 ->106         0.63822
```

```
     103 ->107         0.16985
     103 ->112        -0.15780
 This state for optimization and/or second-order correction.
 Total Energy, E(TD-HF/TD-DFT) =  -1259.47145839
 Copying the excited state density for this state as the 1-particle RhoCI
density.

 Excited State   2:  Triplet-A   2.2994 eV  539.20 nm  f=0.0000
<S**2>=2.000
      99 ->106        -0.32416
      99 ->107        -0.12305
      99 ->109         0.10554
     102 ->106         0.44126
     102 ->107        -0.12646
     105 ->106         0.33278

 Excited State   3:  Triplet-A  3.0365 eV  408.32 nm  f=0.0000
<S**2>=2.000
      99 ->106        -0.14201
     100 ->110        -0.12128
     102 ->106        -0.35048
     102 ->107         0.19191
     105 ->106         0.46246
```

2e,

```
Excited State   1: Singlet-A   2.7719 eV  447.29 nm  f=0.0025  <S**2>=0.000
     118 ->124         0.63125
     119 ->124        -0.24218
 This state for optimization and/or second-order correction.
 Total Energy, E(TD-HF/TD-DFT) =  -1487.99999072
 Copying the excited state density for this state as the 1-particle RhoCI
density.

 Excited State   2: Singlet-A   3.2448 eV  382.10 nm  f=0.0028
<S**2>=0.000
     123 ->124         0.69637

 Excited State   3: Singlet-A   3.4351 eV  360.93 nm  f=0.0176
<S**2>=0.000
     122 ->124         0.67723

 Excited State   4: Singlet-A  3.9295 eV  315.52 nm  f=0.4637  <S**2>=0.000
     121 ->129        -0.10945
     122 ->127        -0.14014
     123 ->126         0.67168

 Excited State   5:  Singlet-A  3.9731 eV  312.06 nm  f=0.1720
<S**2>=0.000
     114 ->124        -0.10797
     116 ->125         0.11871
     119 ->124         0.20320
     120 ->124         0.63321
```

2e, S1 opt

```
Excited State   1: Singlet-A   2.1640 eV  572.93 nm  f=0.0025  <S**2>=0.000
     120 ->124        -0.10339
     121 ->124         0.66726
     121 ->125         0.11256
     121 ->132        -0.12708
 This state for optimization and/or second-order correction.
 Total Energy, E(TD-HF/TD-DFT) =  -1488.01029994
```

```
 Copying the excited state density for this state as the 1-particle RhoCI
density.

 Excited State   2: Singlet-A   3.2304 eV  383.80 nm  f=0.0041
<S**2>=0.000
     123 ->124         0.69467

 Excited State   3: Singlet-A  3.4052 eV  364.10 nm  f=0.0402  <S**2>=0.000
     122 ->124         0.67110
```

2e, at T geom

```
Excited State   1:  Triplet-A   1.8986 eV  653.03 nm  f=0.0000
<S**2>=2.000
     103 ->124         0.10788
     116 ->124         0.45497
     116 ->128        -0.10901
     119 ->124         0.12749
     120 ->124        -0.44017
     120 ->125         0.11156
     122 ->124        -0.14884
     116 <-124         0.13057
     120 <-124        -0.10330
 This state for optimization and/or second-order correction.
 Total Energy, E(TD-HF/TD-DFT) =  -1488.01716911
 Copying the excited state density for this state as the 1-particle RhoCI
density.

 Excited State   2:  Triplet-A  2.0471 eV  605.66 nm  f=0.0000
<S**2>=2.000
     117 ->124         0.63699
     117 ->128         0.11955
     117 ->132        -0.10194
     123 ->124        -0.12159

 Excited State   3: Triplet-A  2.1075 eV  588.31 nm  f=0.0000  <S**2>=2.000
     121 ->127        -0.18052
     121 ->129         0.10066
     122 ->127         0.11038
     123 ->126         0.63324
     123 <-126         0.13370
```

2f,

```
Excited State   1: Singlet-A   2.5531 eV  485.62 nm  f=0.0002  <S**2>=0.000
      98 ->102        -0.10787
     101 ->102         0.67932
 This state for optimization and/or second-order correction.
 Total Energy, E(TD-HF/TD-DFT) =  -1256.03595057
 Copying the excited state density for this state as the 1-particle RhoCI
density.

 Excited State   2:  Singlet-A   2.7849 eV  445.19 nm  f=0.0027
<S**2>=0.000
      98 ->102         0.66991
     101 ->102         0.11500

 Excited State   3:  Singlet-A   3.8223 eV  324.37 nm  f=0.0003
<S**2>=0.000
     101 ->103         0.65236
     101 ->104        -0.18500
```

```
 Excited State   4:  Singlet-A   3.9631 eV  312.85 nm  f=0.1659
<S**2>=0.000
      93 ->102        -0.13837
      94 ->103         0.12290
      99 ->102         0.66856
```

2f, S1 opt

```
Excited State   1:  Singlet-A  1.8261 eV  678.96 nm  f=0.0001  <S**2>=0.000
     101 ->102         0.69516
 This state for optimization and/or second-order correction.
 Total Energy, E(TD-HF/TD-DFT) =  -1256.04986137
 Copying the excited state density for this state as the 1-particle RhoCI
density.

 Excited State   2: Singlet-A  2.7970 eV  443.28 nm  f=0.0038  <S**2>=0.000
      96 ->102         0.68894

 Excited State   3: Singlet-A   3.5387 eV  350.36 nm  f=0.1106
<S**2>=0.000
      93 ->102         0.11162
      94 ->102         0.17594
      99 ->102        -0.66283
```

2f, at T geom

```
Excited State 1:   Triplet-A    1.8133 eV  683.76 nm  f=0.0000
<S**2>=2.000
     101 ->102         0.69471
 This state for optimization and/or second-order correction.
 Total Energy, E(TD-HF/TD-DFT) =  -1256.04981757
 Copying the excited state density for this state as the 1-particle RhoCI
density.

 Excited State   2:  Triplet-A   1.8936 eV  654.75 nm  f=0.0000
<S**2>=2.000
      94 ->102        -0.46104
      99 ->102         0.48535
      99 ->103         0.11643
      94 <-102        -0.13274
      99 <-102         0.11471

 Excited State   3: Triplet-A   2.0616 eV  601.39 nm  f=0.0000
<S**2>=2.000
      96 ->102         0.66639
      96 ->105         0.10623
      96 ->107        -0.10183

 Excited State   4:  Triplet-A   2.6284 eV  471.71 nm  f=0.0000
<S**2>=2.000
      95 ->108        -0.13009
      97 ->110         0.18624
     100 ->106        -0.11561
     101 ->103         0.12784
     101 ->104         0.56920
     101 ->105         0.28399
```

f) intermolecular interaction energy calculations

The spin-spin exchange interaction J_{DFT} for the close pair of molecules in the crystal lattice of **1d** were determined using the Yamaguchi formalism (eq S13):[16]

$$\Delta E_{ST} = 2J_{DFT} = 2\frac{E_{BS}-E_T}{\langle S^2\rangle_T-\langle S^2\rangle_{BS}} \qquad \text{eq S13}$$

where the SCF energies of the triplet (E_T) and broken symmetry singlet (E_{BS}) and total spin angular momenta $\langle S^2\rangle$ before spin annihilation were obtained by single point calculations for selected pairs of molecules at crystallographically determined coordinates at the UB3LYP/6-311+G(d) level of theory.[17] The factor "2" in eq S13 makes the experimental and calculated exchange interactions comparable. The input geometries and calculated exchange interaction energies are shown in Figure S44.

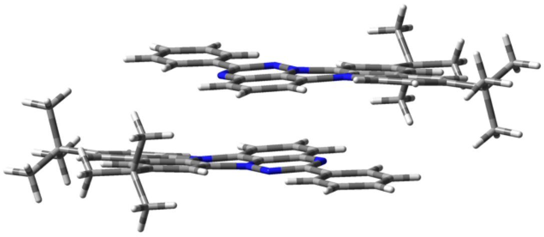

crystallographic close pair J_{DFT} = +76 cal mol^{-1}.

Figure S44. Pairs of molecules of radical **1d** in their crystallographic coordinates used for DFT calculations and the resulting unprojected spin-spin interaction energies.

11. Archive for DFT geometry optimization results.

1a

```
1\1\GINC-GAUSSIAN-PRECISION-WORKSTATION-T7500\FOpt\UB3LYP\6-311G(d,p)\
 C21H13N4(2)\GAUSSIAN\29-Mar-2024\0\\#p freq=noraman b3lyp/6-311g(d,p)
 fopt=tight geom=(nodistance,noangle) #P scf=direct\\benzotrazinyl indo
 le radical\\0,2\N,-0.2511110822,-0.3171637738,0.\N,1.6289855588,1.6830
 637674,0.\N,-0.6556188719,0.9759006692,0.\C,0.3133708925,1.8989729284,
 0.\C,2.8952711992,-2.3104997987,0.\C,1.5342795901,-2.0280865372,0.\C,1
 .1037597319,-0.6808976675,0.\C,2.0399928506,0.3796622589,0.\C,3.411458
 1958,0.0600145627,0.\C,3.8212060139,-1.2608448152,0.\C,-0.7855726906,-
 2.627910587,0.\C,-1.6123626005,-3.7590692972,0.\C,-1.2395237263,-1.312
 6504053,0.\C,-3.0050758522,-3.5430705006,0.\C,-2.618374195,-1.11372015
 89,0.\C,-3.4729650866,-2.2331854636,0.\H,4.1185478641,0.8791687246,0.\
 H,4.8791934103,-1.4953323252,0.\H,3.2344774276,-3.3378814867,0.\C,-0.1
 52036837,3.3127405713,0.\C,-1.5166641831,3.6307772263,0.\C,0.788175195
 1,4.3508111197,0.\C,-1.9292138074,4.9586776679,0.\H,-2.2443846629,2.83
 0695166,0.\C,0.3714731187,5.6777156523,0.\H,1.8393967661,4.0959213915,
 0.\C,-0.9875692323,5.9868671512,0.\H,-2.9880740757,5.1923516,0.\H,1.10
 91592167,6.4724391838,0.\H,-1.3113687628,7.0218078115,0.\C,-0.71388315
 59,-4.8856058731,0.\C,0.5693715808,-4.4016965928,0.\N,0.5348850001,-3.
```

0123690208,0.\H,-3.7000697733,-4.3741111906,0.\H,-4.5423771289,-2.0567
758609,0.\H,-3.0129302095,-0.1085878432,0.\H,-0.983460087,-5.930015412
1,0.\H,1.5059378185,-4.9333584324,0.\\Version=ES64L-G16RevA.03\State=2
-A"\HF=-1027.2850617\S2=0.767107\S2-1=0.\S2A=0.750235\RMSD=1.994e-09\R
MSF=2.623e-07\Dipole=0.1996715,-1.0925411,0.\Quadrupole=4.9272343,7.75
69219,-12.6841561,-4.547521,0.,0.\PG=CS [SG(C21H13N4)]\\@

1b

1\1\GINC-GAUSSIAN-PRECISION-WORKSTATION-T7500\FOpt\UB3LYP\6-311G(d,p)\
C20H12N5(2)\GAUSSIAN\02-Apr-2024\0\\#p freq=noraman b3lyp/6-311g(d,p)
fopt=tight geom=(nodistance,noangle) #P scf=direct\\benzotrazinyl benz
imidazole radical\\0,2\N,-0.2580209781,-0.3147744871,0.\N,1.6373961155
,1.6686033787,0.\N,-0.6521865174,0.9798077744,0.\C,0.3236366748,1.8968
675409,0.\C,2.8827787882,-2.3322958323,0.\C,1.5254713507,-2.0405745925
,0.\C,1.095804918,-0.6921869522,0.\C,2.0385455501,0.3627331809,0.\C,3.
4085727638,0.0348119752,0.\C,3.8142822724,-1.2871027946,0.\C,-0.796329
4934,-2.613150737,0.\C,-1.5925415942,-3.7551889643,0.\C,-1.2536193879,
-1.3048380251,0.\C,-2.9866001998,-3.5800465189,0.\C,-2.6365803877,-1.1
333293133,0.\C,-3.4690347194,-2.2722333856,0.\H,4.1192145892,0.8509497
246,0.\H,4.8711407976,-1.5257016671,0.\H,3.2147425739,-3.3623693214,0.
\C,-0.1305217762,3.3133254018,0.\C,-1.4929608501,3.6411268726,0.\C,0.8
177474965,4.3441758108,0.\C,-1.8954402796,4.9719957201,0.\H,-2.2268969
111,2.8467486328,0.\C,0.4108399798,5.6739936994,0.\H,1.8671980277,4.08
21803598,0.\C,-0.9459524166,5.9929542578,0.\H,-2.9524272935,5.21366595
28,0.\H,1.1542486398,6.4632710029,0.\H,-1.2620256416,7.030227773,0.\C,
0.4600264696,-4.4078857417,0.\N,0.5139333114,-3.020805595,0.\H,-3.6579
17379,-4.4290267466,0.\H,-4.540926322,-2.1125177633,0.\H,-3.0555654798
,-0.1378598007,0.\H,1.3535867479,-5.0124225119,0.\N,-0.7637560792,-4.8
733201475,0.\\Version=ES64L-G16RevA.03\State=2-A"\HF=-1043.3314566\S2=
0.766609\S2-1=0.\S2A=0.750219\RMSD=9.708e-09\RMSF=1.657e-06\Dipole=0.5
396237,-0.0235291,0.\Quadrupole=10.728196,-2.8016514,-7.9265446,-8.579
4856,0.,0.\PG=CS [SG(C20H12N5)]\\@

1c

1\1\GINC-GAUSSIAN-PRECISION-WORKSTATION-T7500\FOpt\UB3LYP\6-311G(d,p)\
C25H15N4(2)\GAUSSIAN\12-Sep-2024\0\\#p freq=noraman ub3lyp/6-311g(d,p)
fopt=tight geom=(nodistance,noangle) #P scf=direct\\benzotrazinyl Tcar
bazole radicaled\\0,2\N,0.5612694871,-0.2876287692,-0.0010660458\N,2.5
14264207,1.6336923849,-0.0045462006\N,1.863322225,-0.6638914008,0.0032
350917\C,2.7635729652,0.3252671701,0.0026719566\C,-1.4984907966,2.8091
046237,-0.0180882543\C,-1.2051393433,1.4480547953,-0.0026228977\C,0.15
87163934,1.0551794067,-0.0023504062\C,0.8594935027,3.3781145853,-0.017
7092538\C,-0.4676969783,3.7563520122,-0.0248597292\C,-2.8098449352,-1.
7880222078,-0.0072403421\C,-0.4044953119,-1.3009994938,-0.0098538808\C
,-2.5392423384,-3.1642558359,-0.0191438794\C,-0.1416891951,-2.67123958
64,-0.0216081884\C,-1.2105045659,-3.5798115692,-0.0265940346\H,1.66576
62705,4.0996739571,-0.0216075315\H,-0.7289344108,4.8080387935,-0.03630
23097\H,-2.5200063576,3.1474509993,-0.030421616\C,4.1882177077,-0.1055
020973,0.009941771\C,4.5399266825,-1.461700747,0.0244762415\C,5.202742
5057,0.8599904644,0.0023434691\C,5.8775975205,-1.8413602184,0.03105577
64\H,3.7582661984,-2.2091582666,0.0309968677\C,6.5395430978,0.47627179
15,0.0088184507\H,4.9218105229,1.9045077584,-0.0086430504\C,6.88219688
85,-0.8746333635,0.023146879\H,6.1373941246,-2.8940625112,0.0424788577
\H,7.3157950581,1.2333434245,0.0026831895\H,7.9248136455,-1.1727412904
,0.028244325\C,-3.9985837876,-0.9598911128,0.0085912324\C,-3.578080288
3,0.4022351873,0.0212722149\N,-2.1709106914,0.423795551,0.008818416\C,
-5.3583597279,-1.2727104162,0.0172650083\H,-5.6784999421,-2.3083903671
,0.0072460807\C,-6.2919728064,-0.2451943158,0.0404832995\H,-7.35086384
16,-0.4746461217,0.0473596954\C,-5.8710315553,1.0896966608,0.057899506
9\H,-6.6097000613,1.8825937674,0.0798395844\C,-4.5221868419,1.43188724

76,0.0493451717\H,-4.246902966,2.4740121813,0.0703345236\H,-3.34197778
53,-3.8915703734,-0.02164173\H,-0.985745666,-4.6397932263,-0.035688716
3\H,0.8832838825,-3.0105186925,-0.0261753693\C,-1.7355277059,-0.895299
9241,-0.0044415934\C,1.200566014,2.0131711451,-0.0079425801\\Version=E
S64L-G16RevA.03\State=2-A\HF=-1180.9673308\S2=0.765862\S2-1=0.\S2A=0.7
50201\RMSD=4.880e-09\RMSF=2.373e-07\Dipole=-1.092949,-0.0313867,0.0014
335\Quadrupole=6.4768953,7.3119803,-13.7888756,-3.079821,-0.0684126,0.
0177513\PG=C01 [X(C25H15N4)]\\@

1c-*oxo*

1\1\GINC-GAUSIANDELL\FOpt\RB3LYP\6-311G(d,p)\C25H14N4O1\PKASZYNSKI\29-
Mar-2024\0\\#P B3LYP/6-311(d,p) FOpt=tight SCRF=(solvent=Benzene) geom
=(noangle,nodistance)\\Flat Blatter Carbazole oxidized, Cs\\0,1\N,-0.6
680907945,-0.1769131444,0.\N,-2.7115694909,1.6609592209,0.\N,-1.944886
9933,-0.5976317841,0.\C,-2.8992054155,0.3512494707,0.\C,1.2470088512,3
.0117103858,0.\C,1.0177077872,1.6384390702,0.\C,-0.3334859955,1.184745
4477,0.\C,-1.4226137302,2.0974693371,0.\C,-1.1396660572,3.4744863234,0
.\C,0.171306267,3.9062906407,0.\C,1.6493483433,-0.6686953042,0.\C,2.75
06240372,-1.4977259088,0.\C,0.3156566222,-1.1136374437,0.\C,2.57909862
14,-2.9407811326,0.\C,0.1013305041,-2.5299531797,0.\C,1.1632278567,-3.
378721855,0.\H,-1.9731481965,4.1639812728,0.\H,0.3846430555,4.96874927
66,0.\H,2.2506432964,3.3986076475,0.\C,-4.2959747039,-0.1580737769,0.\
C,-4.5709226188,-1.5325093479,0.\C,-5.362640775,0.7507505348,0.\C,-5.8
859871773,-1.9855314828,0.\H,-3.7514276566,-2.2381104054,0.\C,-6.67564
58007,0.2936103579,0.\H,-5.1439726358,1.8100847362,0.\C,-6.9420277935,
-1.0753124483,0.\H,-6.0870328606,-3.050755078,0.\H,-7.4927557245,1.006
0050768,0.\H,-7.9663374614,-1.4306433664,0.\C,3.8992753277,-0.63435033
14,0.\C,3.4309120691,0.7127630891,0.\N,2.019446297,0.6672995413,0.\C,5
.2730823296,-0.8993458085,0.\H,5.6147064736,-1.9259693082,0.\C,6.15838
35969,0.1681123164,0.\C,5.6893168076,1.4904404326,0.\H,6.3990378336,2.
3093086529,0.\C,4.3296732613,1.7826936046,0.\H,4.0160897731,2.81423108
06,0.\H,-0.9150102418,-2.8964552248,0.\H,7.2260954651,-0.0176885041,0.
\H,1.0072588523,-4.4511544099,0.\O,3.5099079944,-3.7510778014,0.\\Vers
ion=ES64L-G09RevD.01\State=1-A'\HF=-1255.6105422\RMSD=8.180e-09\RMSF=1
.962e-06\Dipole=-0.9673213,2.1416958,0.\Quadrupole=5.2461145,1.958586,
-7.2047005,20.6217421,0.,0.\PG=CS [SG(C25H14N4O1)]\\@

1d

1\1\GINC-GAUSSIAN-PRECISION-WORKSTATION-T7500\FOpt\UB3LYP\6-311G(d,p)\
C33H31N4(2)\GAUSSIAN\26-Mar-2024\0\\#p freq=noraman b3lyp/6-311g(d,p)
fopt=tight geom=(nodistance,noangle) #P scf=direct\\benzotrazinyl carb
azole\\0,2\N,1.7099182611,-0.4674908509,0.0578949741\N,3.7113165241,-2
.3387172277,0.0025449922\N,3.0023513216,-0.0591601479,0.0739524265\C,3
.9268788147,-1.0251504173,0.0451299235\C,-0.2710914657,-3.6164962878,-
0.0475520422\C,-0.0117519628,-2.2488757001,-0.003132387\C,1.341774426
3,-1.8201349667,0.0137138984\C,2.4073144008,-2.7513866876,-0.013675189
3\C,2.1009398723,-4.1237477029,-0.0583515721\C,0.7835162719,-4.5361981
612,-0.0745970468\C,-0.5996704849,0.0752709425,0.0699703926\C,-1.68762
01276,0.9437721374,0.0952892653\C,0.7166397963,0.5203032483,0.08724931
38\C,-1.4492052413,2.3267910148,0.1401338076\C,0.9403313523,1.89322716
69,0.1317364445\C,-0.1370370513,2.8081729696,0.1585658666\H,2.92538586
37,-4.824201351,-0.0788996757\H,0.5501020601,-5.5939625283,-0.10902804
95\H,-1.2850887857,-3.9781847554,-0.0614773978\C,5.3406862374,-0.55920
68676,0.0633145873\C,5.6591850876,0.8044929742,0.1077594791\C,6.378850
6516,-1.498825565,0.0355818891\C,6.9871030307,1.2168595187,0.124029892
1\H,4.859288013,1.5321145137,0.1292455085\C,7.7058394802,-1.0825907791
,0.0519734488\H,6.1234817054,-2.5493841051,0.0013203686\C,8.0152923097
,0.2756466816,0.0962224197\H,7.2209016221,2.2751771717,0.1584917781\H,
8.5004572171,-1.8200989979,0.0301589793\H,9.0502635051,0.5991339812,0.
1089703985\C,-2.8594719504,0.0892902273,0.0652318419\C,-2.4092177135,-

1.2581542617,0.0231102202\N,-1.0020332731,-1.2520408216,0.026533702\C,
-4.2263148154,0.3778283882,0.0713380644\H,-4.5367261336,1.4137729279,0
.1038037272\C,-5.169717112,-0.6472413509,0.0363459042\C,-4.6920562256,
-1.9734599119,-0.0050294197\H,-5.4012590967,-2.7921556552,-0.032872499
1\C,-3.3413131966,-2.297088299,-0.0122965326\H,-3.0551424668,-3.336665
3441,-0.0449276261\H,-2.2865852349,3.0100152285,0.1600937188\H,1.96383
93896,2.235471446,0.1450495595\C,0.1849554458,4.3146372714,0.207554907
6\C,-6.6866971972,-0.3861022063,0.0412299359\C,-1.0852816509,5.1842965
485,0.2324915689\H,-1.6980663839,5.0363262817,-0.6609222064\H,-1.70213
74319,4.9795495439,1.1117845401\H,-0.8040334785,6.240032037,0.26695086
28\C,1.0034940724,4.6263430262,1.4821353251\H,1.9463007716,4.075626872
,1.5055385717\H,1.239290309,5.69390457,1.5293691885\H,0.4401542703,4.3
621848761,2.3812203104\C,-7.0193340587,1.1162985156,0.0885796324\H,-6.
6229515491,1.6484367697,-0.7805194906\H,-8.104084967,1.2515307641,0.09
04223718\H,-6.6270163713,1.5916817701,0.9917524807\C,-7.3227315095,-1.
0575879528,1.2807413972\H,-7.1632421483,-2.1382648621,1.2848884549\H,-
6.8977765401,-0.6538106102,2.203650499\H,-8.4025546157,-0.8804764725,1
.2968024362\C,1.0092878092,4.7071449246,-1.0406978661\H,1.2451415866,5
.7755063256,-1.0184387183\H,1.9522727682,4.1589163436,-1.0949676991\H,
0.4501179887,4.5011440303,-1.9574190322\C,-7.3169476369,-0.976813005,-
1.24160545\H,-6.8878278269,-0.5148765508,-2.1348326929\H,-7.1572828331
,-2.0550338014,-1.3141563511\H,-8.3967117196,-0.7988779436,-1.25124882
03\\Version=ES64L-G16RevA.03\State=2-A\HF=-1495.5583069\S2=0.765625\S2
-1=0.\S2A=0.750195\RMSD=6.686e-09\RMSF=3.097e-07\Dipole=-1.2910709,0.2
0343,0.003556\Quadrupole=3.906653,8.6423632,-12.5490162,4.9583935,0.19
66713,0.6910214\PG=C01 [X(C33H31N4)]\\@

1e

1\1\GINC-GAUSSIAN-PRECISION-WORKSTATION-T7500\FOpt\UB3LYP\6-311G(d,p)\
C33H19N4(2)\GAUSSIAN\30-Mar-2024\0\\#p freq=noraman b3lyp/6-311g(d,p)
fopt=tight geom=(nodistance,noangle) #P scf=direct\\benzotrazinyl Benz
carbazole radical\\0,2\N,-0.2926326694,0.5747248284,0.0207453568\N,1.6
395178259,2.5088824753,-0.1788351282\N,-0.6577027261,1.8799525043,-0.0
068713212\C,0.3361212993,2.7711330907,-0.0949154815\C,2.7686077681,-1.
5147612835,-0.0999012425\C,1.4195719112,-1.1976094156,0.0439310847\C,1
.0422338283,0.165602671,-0.0136980693\C,2.0076164052,1.1937937657,-0.1
447831891\C,3.3633386069,0.8329792547,-0.244673773\C,3.7231515481,-0.5
000679106,-0.2365427975\C,-0.9300115505,-1.727283843,0.1376091588\C,-1
.8102609817,-2.8057584514,0.1875092558\C,-1.3184093278,-0.3771758041,0
.0519921631\C,-3.2178193449,-2.5075431539,0.3120366838\C,-2.6621986914
,-0.0887125428,0.0157878884\C,-3.6192384942,-1.1326231797,0.1635083264
\H,4.0916349202,1.6271635433,-0.3426217968\H,4.7651211518,-0.778360487
8,-0.341861171\H,3.086312284,-2.5427668675,-0.1385761886\C,-0.08283913
93,4.1989505008,-0.1046920204\C,-1.4275249523,4.5648596789,0.040558491
7\C,0.8823837646,5.201892228,-0.2596271839\C,-1.7964079635,5.905487414
7,0.0296772588\H,-2.1740827242,3.7923086678,0.165350784\C,0.5091629146
,6.5415618369,-0.271458675\H,1.9182976284,4.910388062,-0.3689790096\C,
-0.8303912486,6.8984540903,-0.127164966\H,-2.83993201,6.1767051766,0.1
446011547\H,1.2655008579,7.3088793278,-0.3933145606\H,-1.1201599936,7.
9433643978,-0.1358396514\C,-0.9606542439,-3.9878721141,0.2135265532\C,
0.3761302116,-3.5360135558,0.3144899275\N,0.3872761697,-2.1427070271,0
.2040917758\C,-1.1932835115,-5.4016438659,0.1000014121\C,-0.0970256276
,-6.2873665506,0.3640561409\C,1.1934793484,-5.7576738376,0.6465325414\
H,1.9970974732,-6.4467541453,0.8810681041\C,1.4492544602,-4.4150183379
,0.5836007486\H,2.4420576337,-4.0483829907,0.7913252948\H,-2.986856669
8,0.9378176817,-0.0687035692\C,-4.2151491489,-3.4442061881,0.665157941
7\C,-4.9965550104,-0.8055821896,0.2393701565\C,-5.544435451,-3.0853112
024,0.7637042195\C,-5.9463774988,-1.7603272817,0.5187968126\H,-6.99370
24298,-1.488151821,0.580949482\H,-5.2870856786,0.2298599798,0.09751605
51\H,-6.2801325371,-3.8305700425,1.0441877415\H,-3.93003336,-4.4571435

571,0.9042605735\C,-2.4051443681,-5.9795021571,-0.3541675365\H,-3.2068
722215,-5.3368861329,-0.6851269301\C,-0.299521668,-7.6874232257,0.2911
318364\H,0.5345142195,-8.3422860197,0.5211290815\C,-2.5604938815,-7.34
52076437,-0.4476201069\H,-3.4961608615,-7.7530060564,-0.8134811636\C,-
1.5099967219,-8.2136495563,-0.0926793136\H,-1.6475043232,-9.2870349473
,-0.1534150699\\Version=ES64L-G16RevA.03\State=2-A\HF=-1488.3115342\S2
=0.768329\S2-1=0.\S2A=0.750272\RMSD=7.510e-09\RMSF=1.656e-07\Dipole=0.
1438285,-0.8487309,0.0495424\Quadrupole=9.8586221,6.2579398,-16.116561
8,-5.0854711,0.1243348,-0.8142658\PG=C01 [X(C33H19N4)]\\@

1f

1\1\GINC-GAUSSIAN-PRECISION-WORKSTATION-T7500\FOpt\UB3LYP\6-311G(d,p)\
C25H15N4O1(2)\GAUSSIAN\02-Apr-2024\0\\#p freq=noraman b3lyp/6-311g(d,p
) fopt=tight geom=(nodistance,noangle) #P scf=direct\\benzotrazinyl ph
enoxazine radical\\0,2\N,-0.2996144073,0.6893061403,0.2192479929\N,1.7
02067982,2.560939027,0.368458041\N,-0.5977143443,1.9984079299,0.025482
6744\C,0.4308874929,2.8523894193,0.0925126788\C,2.6138790104,-1.497302
0539,0.8358591102\C,1.3067520986,-1.1302904866,0.512220219\C,1.0090700
469,0.2430229028,0.4091736218\C,2.0076296536,1.2374520787,0.5298429011
\C,3.3222326404,0.8269450772,0.8117595078\C,3.6027522803,-0.5178723755
,0.9778967139\C,-1.0637331684,-1.5748359426,0.4522338849\C,-2.09710657
27,-2.488359879,0.657140439\C,-1.3685648786,-0.2078297291,0.3797131015
\C,-3.4141450735,-2.0701190336,0.7719220289\C,-2.6920998492,0.22687813
53,0.5102354215\C,-3.7015058186,-0.7064319198,0.702065613\H,4.08447856
5,1.5896659957,0.9018316021\H,4.6111474741,-0.8295736983,1.2244627149\
H,2.8595375257,-2.5383847077,0.985967565\C,0.0902427994,4.2798209433,-
0.1567694947\C,-1.195815358,4.662226884,-0.5606554453\C,1.0715822399,5
.2649565752,0.0100952025\C,-1.4920257309,6.0015052907,-0.7895385105\H,
-1.9526722074,3.9018857598,-0.6992138146\C,0.7706282027,6.6037024331,-
0.217155939\H,2.0626450049,4.9598038071,0.3180163005\C,-0.5111929366,6
.9772726461,-0.6171756765\H,-2.4901666956,6.2848807007,-1.1046146169\H
,1.5385594637,7.3572978537,-0.0822973062\H,-0.7443178204,8.0212376183,
-0.7953702261\C,-0.6646303496,-4.2652805911,0.1243999545\C,0.402182249
3,-3.3865147357,-0.1215183881\N,0.2567744401,-2.0461778681,0.316006916
8\O,-1.8096808953,-3.8326583479,0.7683123012\C,-0.610563232,-5.5922020
544,-0.2719345753\H,-1.4584639661,-6.2285862198,-0.050888304\C,0.51674
80169,-6.0724144586,-0.9350126976\H,0.565275646,-7.1127941679,-1.23227
28304\C,1.5624468851,-5.2031399079,-1.2280928206\H,2.4318107494,-5.556
5591064,-1.7694421214\C,1.5018416036,-3.8667780143,-0.8380372558\H,2.3
096937413,-3.1956201908,-1.0924813075\H,-4.1864681402,-2.8124991014,0.
9258011734\H,-4.7261237735,-0.3704942661,0.8033265457\H,-2.894220464,1
.2860953179,0.4663397138\\Version=ES64L-G16RevA.03\State=2-A\HF=-1256.
1843188\S2=0.764376\S2-1=0.\S2A=0.75016\RMSD=8.537e-09\RMSF=3.290e-07\
Dipole=-0.0770277,-1.1020447,-0.1659398\Quadrupole=5.8453873,5.1448637
,-10.990251,-4.5257557,-0.0659791,1.7876395\PG=C01 [X(C25H15N4O1)]\\@

2a, GS

1\1\GINC-LOCALHOST\FOpt\RCAM-B3LYP\6-311G(d,p)\C21H14N4\PIOTR\27-Dec-2
024\0\\#P CAM-B3LYP/6-311G(d,p) FOpt=tight SCF=Direct freq(noraman) #P
Geom=(NoDistance,NoAngle) fcheck SCRF(Solvent=EthylEthanoate)\\benzot
razine indole\\0,1\N,-0.2145378739,-0.3101522597,-1.2667580259\N,-0.64
8720431,2.3984149336,-1.4202752345\N,0.6327431304,0.4531548031,-1.8604
603878\C,0.4096899345,1.8020190268,-1.9124268653\C,-3.4116686408,-0.04
36890055,0.4453530212\C,-2.2668471162,-0.6078466966,-0.0445106732\C,-1
.3203223192,0.2299443847,-0.7055650605\C,-1.5585039747,1.6177036723,-0
.8070203416\C,-2.7508151677,2.1701302864,-0.2851758024\C,-3.6547948259
,1.3444599236,0.3147151594\C,-1.0045958056,-2.6394604372,0.7111952505\
C,-1.2586201686,-4.024904774,0.6554115506\C,0.107415189,-2.1194024934,
1.3702492899\C,-0.3556305863,-4.9096693477,1.2561418466\C,0.9779310347
,-3.014741865,1.9591299565\C,0.7526665963,-4.3994852904,1.8998061662\H

,-2.9125735565,3.2354827478,-0.381171527\H,-4.5744087723,1.7499044978,
0.7175146701\H,-4.1356607179,-0.6690972028,0.9514350069\C,1.450237888,
2.610507764,-2.5862384723\C,2.5811323244,2.0055019576,-3.1353211285\C,
1.3042169486,3.9953056278,-2.6757304512\C,3.5478259863,2.7757018308,-3
.7630418049\H,2.6923783709,0.9326697383,-3.0653455574\C,2.2727008413,4
.7608687017,-3.3032157217\H,0.4253414474,4.4589241853,-2.2486765766\C,
3.3971436893,4.1532745642,-3.8486401357\H,4.4224767726,2.2976690619,-4
.1869720986\H,2.150865906,5.8352750618,-3.3676116621\H,4.1541385289,4.
7531158519,-4.3394754067\C,-2.4973431035,-4.1989487723,-0.0495303397\C
,-2.9439175478,-2.9618643165,-0.3790495585\N,-2.0488671869,-1.99743374
4,0.0609962057\H,-0.5309703903,-5.9785777929,1.2189195857\H,1.45998016
14,-5.072069277,2.3699574261\H,1.8537665673,-2.6404718069,2.4753249016
\H,-2.9843074624,-5.1312799638,-0.2870915439\H,-3.8265045309,-2.661262
0701,-0.9207814036\H,0.2907896813,-1.0543586156,1.4169325525\\Version=
ES64L-G16RevC.01\State=1-A\HF=-1027.3354817\RMSD=9.766e-09\RMSF=1.311e
-06\Dipole=-1.3691419,1.258514,0.2137499\Quadrupole=9.9694998,-0.97609
25,-8.9934072,2.1664667,-4.5510987,-0.7268779\PG=C01 [X(C21H14N4)]\\@

2a, S1

1\1\GINC-LOCALHOST\FOpt\RCAM-B3LYP TD-FC\6-311G(d,p)\C21H14N4\PIOTR\31
-Dec-2024\0\\#P CAM-B3LYP/6-311G(d,p) Fopt TD=(singlets,root=1, NState
s=3) SCF=tight #P Geom=(NoDistance,NoAngle) fcheck SCRF(Solvent=EthylE
thanoate)\\benzotrazine Benzcarbazole TD ex geom\\0,1\N,-0.2167145005,
-0.2526645528,-1.21203971\N,-0.6563462905,2.3976746777,-1.3954742582\N
,0.5817607597,0.5191716905,-1.7706316528\C,0.4334791824,1.8481403354,-
1.8996007713\C,-3.4754782833,-0.0358830339,0.4948503803\C,-2.310657527
3,-0.6082356017,-0.0083300608\C,-1.3780781302,0.2071713079,-0.64781768
24\C,-1.5883906261,1.62170029,-0.772950714\C,-2.7684072994,2.149764044
5,-0.2549739385\C,-3.699395649,1.3256379993,0.3632173887\C,-1.02779375
1,-2.6327853184,0.7221362907\C,-1.2375728683,-4.0235028659,0.614105115
4\C,0.0687048383,-2.0999661407,1.3985146151\C,-0.3059075432,-4.8989592
178,1.1838092439\C,0.969277492,-2.9879522198,1.9527884818\C,0.78739662
42,-4.376171596,1.8436806424\H,-2.9354340835,3.2141670557,-0.350604187
6\H,-4.6095781929,1.7563944429,0.760225634\H,-4.1944950566,-0.66970177
49,0.9971191441\C,1.4731029183,2.6215351995,-2.5852223751\C,2.60930709
95,1.993326573,-3.0939385816\C,1.3222719124,3.9994688353,-2.7291781564
\C,3.5824758913,2.7371048502,-3.7392374843\H,2.7239032059,0.9222494082
,-2.9803104989\C,2.2993042665,4.7395201698,-3.3758590456\H,0.436204193
4,4.4753196505,-2.3303270953\C,3.4299057864,4.1114816305,-3.8817240016
\H,4.4630643507,2.2445449004,-4.1326819602\H,2.1780059169,5.8101742792
,-3.485585912\H,4.1925403789,4.6915725707,-4.3869895269\C,-2.469086786
8,-4.2083829155,-0.09968603\C,-2.9499617075,-2.9725386418,-0.388358543
5\N,-2.0885323667,-2.0002587681,0.0943406588\H,-0.4469522809,-5.971124
3661,1.1106206898\H,1.5162545891,-5.0423552121,2.289480058\H,1.8331865
767,-2.6053903442,2.4828729003\H,-2.9302080365,-5.1453938435,-0.368439
2308\H,-3.8392929014,-2.678348765,-0.9226774097\H,0.2119687192,-1.0311
918429,1.4878743941\\Version=ES64L-G16RevC.01\State=1-A\HF=-1027.32349
55\RMSD=6.668e-09\RMSF=6.873e-06\Dipole=-0.3609147,1.007194,-0.3442113
\PG=C01 [X(C21H14N4)]\\@

2a, T

1\1\GINC-LOCALHOST\FOpt\UCAM-B3LYP\6-311G(d,p)\C21H14N4(3)\PIOTR\31-De
c-2024\0\\#P UCAM-B3LYP/6-311G(d,p) FOpt=tight SCF=Direct freq(noraman
) #P Geom=(NoDistance,NoAngle) fcheck SCRF(Solvent=EthylEthanoate)\\be
nzotrazine Benzcarbazole t\\0,3\N,-0.2205321206,-0.2524318672,-1.22463
0168\N,-0.6522649394,2.40200827,-1.4077192029\N,0.6015853956,0.5112329
914,-1.7733130034\C,0.4254390189,1.8468985373,-1.901849799\C,-3.464444
9082,-0.0330483384,0.4984737518\C,-2.3074001548,-0.6094655404,-0.00859
56574\C,-1.3720975902,0.204701525,-0.6518520502\C,-1.582712279,1.61259
83595,-0.7740404624\C,-2.7571591148,2.1486590764,-0.2539360762\C,-3.68
89011257,1.3315865518,0.3686053554\C,-1.0309019418,-2.6355522538,0.726

7592458\C,-1.2377234206,-4.0262990586,0.6133298628\C,0.0600079106,-2.1
025173535,1.4119592797\C,-0.3084381609,-4.9016387313,1.1872925514\C,0.
9582646783,-2.99046175,1.9700650033\C,0.7793146755,-4.3787119701,1.856
0907951\H,-2.9201605445,3.2135836414,-0.3531139697\H,-4.5969420786,1.7
630152782,0.7693361788\H,-4.1836481949,-0.664877977,1.0036097209\C,1.4
673190371,2.6252132501,-2.5885439901\C,2.5991488509,1.9959074118,-3.10
33807466\C,1.3201956214,4.0040938683,-2.7261757919\C,3.5737014484,2.73
94670947,-3.7485548139\H,2.7104639643,0.9240546299,-2.9954499362\C,2.2
970766107,4.7439150143,-3.3723326603\H,0.4367172148,4.480152017,-2.322
3462853\C,3.4246486494,4.1141366251,-3.8842303829\H,4.4514481919,2.245
8553674,-4.1469360989\H,2.1790212049,5.8153950956,-3.4772074871\H,4.18
74509822,4.6943311229,-4.3892287074\C,-2.4630999508,-4.2108409079,-0.1
108840531\C,-2.9429956798,-2.9747153168,-0.4007724446\N,-2.0870177367,
-2.0027817083,0.0914510622\H,-0.4468410177,-5.9738626446,1.1100449391\
H,1.5061831391,-5.0448385904,2.3052000298\H,1.8178252574,-2.6078993796
,2.5071502467\H,-2.9209411396,-5.1477328621,-0.3855839608\H,-3.8283906
806,-2.6802020808,-0.9414578821\H,0.2006317481,-1.033614507,1.50576441
75\\Version=ES64L-G16RevC.01\State=3-A\HF=-1027.2668557\S2=2.028824\S2
-1=0.\S2A=2.000404\RMSD=8.662e-09\RMSF=9.653e-07\Dipole=-0.3580375,0.9
475862,-0.336578\Quadrupole=8.7809435,-0.711524,-8.0694195,3.5948009,-
5.4376822,-1.2416008\PG=C01 [X(C21H14N4)]\\@

2b, GS

1\1\GINC-GAUSSIAN-PRECISION-WORKSTATION-T7500\FOpt\RCAM-B3LYP\6-311G(d
,p)\C20H13N5\GAUSSIAN\02-Jan-2025\0\\#P CAM-B3LYP/6-311G(d,p) FOpt=tig
ht SCF=Direct freq(noraman) #P Geom=(NoDistance,NoAngle) SCRF(Solvent=
EthylEthanoate)\\benzotrazine benzimidazole\\0,1\N,-0.6954162497,0.619
1849503,1.6329729013\N,-1.2019154127,-1.9829198826,2.3567473074\N,-0.0
052793933,0.0542716985,2.5586595149\C,-0.2615178114,-1.2464723083,2.89
78743605\C,-3.456000922,-0.173132247,-0.5984315273\C,-2.4413856701,0.5
336404691,-0.0195940722\C,-1.670171107,-0.0802848961,1.0090281857\C,-1
.9455987553,-1.4094407811,1.3926812963\C,-3.0041388988,-2.117158209,0.
7755638752\C,-3.7415158881,-1.4989269677,-0.189444022\C,-1.0242638729,
2.4017112635,-0.9572678323\C,-1.3361312813,3.7427589647,-1.2238542852\
C,0.2141473981,1.8503029618,-1.2657502569\C,-0.3831088998,4.578720636,
-1.804573478\C,1.1443561588,2.6935155786,-1.8444569195\C,0.8523008188,
4.041125974,-2.1087251672\H,-3.199203094,-3.1333179347,1.0914207862\H,
-4.5556292674,-2.0258548873,-0.6705828871\H,-4.0405249621,0.2827589914
,-1.3870137986\C,0.595997638,-1.8264380005,3.9548891082\C,1.5972725982
,-1.0661212878,4.5598810066\C,0.4062339438,-3.1498684915,4.3544691796\
C,2.3937708998,-1.6235394557,5.5480192863\H,1.7426701723,-0.0410643702
,4.2494608753\C,1.2046284915,-3.7026119944,5.3417039111\H,-0.371555085
2,-3.7345022162,3.882291415\C,2.200484624,-2.9410535653,5.9411507325\H
,3.1685849214,-1.0261871801,6.0127926722\H,1.0503642285,-4.7308644532,
5.6453060452\H,2.824464124,-3.3745002866,6.7134882507\C,-3.0972039661,
2.8971357878,-0.3794528317\N,-2.1826187903,1.8685429238,-0.407154907\H
,-0.6195047261,5.6155089857,-2.0086503652\H,1.6125908961,4.6665333424,
-2.5603606264\H,2.1235349239,2.305643689,-2.0974182732\H,-4.0887052522
,2.7361950897,0.0187978347\N,-2.641824153,4.0181207832,-0.8431302078\H
,0.4474883717,0.8145595658,-1.0585062073\\Version=ES64L-G16RevA.03\Sta
te=1-A\HF=-1043.3927245\RMSD=4.224e-09\RMSF=1.420e-06\Dipole=-0.776507
5,-2.4035135,-0.0242589\Quadrupole=9.7354998,-9.4365462,-0.2989536,7.7
176772,5.6661288,1.8045209\PG=C01 [X(C20H13N5)]\\@

2b, S_1 state

1\1\GINC-GAUSSIAN-PRECISION-WORKSTATION-T7500\FOpt\RCAM-B3LYP TD-FC\6-
311G(d,p)\C20H13N5\GAUSSIAN\05-Jan-2025\0\\#P CAM-B3LYP/6-311G(d,p) Fo
pt TD=(singlets,root=1, NStates=3) SCF=tight #P Geom=(NoDistance,NoAng
le) SCRF(Solvent=EthylEthanoate)\\benzotrazine Benzcarbazole TD ex\\0,
1\N,-0.6911429097,0.5571658218,1.6107405235\N,-1.2016388889,-1.9865072
385,2.3336546365\N,-0.0435516174,-0.0294916676,2.4960478749\C,-0.23667

7359,-1.2933288146,2.9092398326\C,-3.5062528246,-0.1858046227,-0.64906
18821\C,-2.4742436207,0.5320024367,-0.0544001075\C,-1.7113386496,-0.06
82202322,0.9452666582\C,-1.9652033731,-1.4187503955,1.3582396021\C,-3.
0111932029,-2.1000317463,0.7398593881\C,-3.7719704242,-1.4854166865,-0
.245466666\C,-1.0431494093,2.3979167558,-0.9658153632\C,-1.3267607695,
3.7537514993,-1.188550909\C,0.1858996177,1.8303475403,-1.2848916971\C,
-0.3532268057,4.5875692905,-1.7372997017\C,1.1372071661,2.673422722,-1
.8291661291\C,0.8734856219,4.0345086072,-2.0507478707\H,-3.2102702192,
-3.1168729205,1.0502828158\H,-4.5783392572,-2.0341340123,-0.714397959\
H,-4.0875759559,0.2813456885,-1.433102689\C,0.6189303547,-1.840961691,
3.9657117244\C,1.6308434334,-1.0681929265,4.534139761\C,0.4170206923,-
3.1480956468,4.4047782099\C,2.4310740605,-1.599339831,5.5311797936\H,1
.7853073999,-0.0526751666,4.1904274982\C,1.2207400251,-3.6750042244,5.
4030817813\H,-0.3714969132,-3.7374898194,3.9558867309\C,2.2279215673,-
2.9033954125,5.967775587\H,3.2157622889,-0.9955232054,5.9698527789\H,1
.0606162689,-4.6911875932,5.7417541941\H,2.855142309,-3.3170000004,6.7
480959316\C,-3.10476812,2.9142025181,-0.3735369167\N,-2.2118305014,1.8
70295981,-0.4359535226\H,-0.5667741196,5.6348643678,-1.9107887424\H,1.
6488386989,4.6585516171,-2.4780662908\H,2.1095938555,2.2742930844,-2.0
907736076\H,-4.1000507875,2.7598942659,0.017938357\N,-2.6279974085,4.0
413552029,-0.8033826636\H,0.3927465268,0.7819086943,-1.1157200817\\Ver
sion=ES64L-G16RevA.03\State=1-A\HF=-1043.3807782\RMSD=5.977e-09\RMSF=6
.201e-06\Dipole=0.1066815,-2.0058041,0.6427243\PG=C01 [X(C20H13N5)]\\@

2b, T

1\1\GINC-GAUSSIAN-PRECISION-WORKSTATION-T7500\FOpt\UCAM-B3LYP\6-311G(d
,p)\C20H13N5(3)\GAUSSIAN\04-Jan-2025\0\\#P UCAM-B3LYP/6-311G(d,p) FOpt
=tight SCF=Direct freq(noraman) #P Geom=(NoDistance,NoAngle) SCRF(Solv
ent=EthylEthanoate)\\benzotrazine Benzcarbazole t\\0,3\N,-0.6947896692
,0.5591266262,1.621191604\N,-1.198316855,-1.9895529394,2.34493034\N,-0
.0275383887,-0.0203298877,2.5041659764\C,-0.2441519372,-1.2936006989,2
.9094241565\C,-3.4948869465,-0.1873968562,-0.6479166009\C,-2.470487792
4,0.5344543548,-0.0525155684\C,-1.7054977582,-0.0641634815,0.950523001
8\C,-1.9586984885,-1.4096651032,1.3580311925\C,-2.9991066159,-2.098345
9769,0.7405674338\C,-3.760477655,-1.4906403352,-0.2463192432\C,-1.0451
518216,2.4002268179,-0.9715964557\C,-1.3262393642,3.7578830495,-1.1864
012458\C,0.179332032,1.8297400113,-1.3028169353\C,-0.3544418143,4.5905
508409,-1.740070183\C,1.1289178644,2.6718327322,-1.8516530181\C,0.8677
347591,4.0346049488,-2.0658359532\H,-3.1944755535,-3.1152205475,1.0539
874535\H,-4.5641925378,-2.0404541866,-0.7181653561\H,-4.0762388605,0.2
778491724,-1.4334612029\C,0.6127076564,-1.8451696527,3.9689717105\C,1.
6183697565,-1.0696708422,4.5429035394\C,0.4161846352,-3.1548549568,4.4
02671319\C,2.4195256337,-1.6003811285,5.5404964211\H,1.7682963512,-0.0
519649418,4.2041328171\C,1.2195292354,-3.6814712352,5.4007780608\H,-0.
3681691487,-3.7458608337,3.9490728437\C,2.2216902917,-2.9064711313,5.9
710307989\H,3.199791088,-0.9941347045,5.9836331339\H,1.0638936474,-4.6
996790609,5.7353779513\H,2.8488432111,-3.3200953959,6.7514591674\C,-3.
0992070131,2.9208230598,-0.3577358667\N,-2.2104660957,1.8744183952,-0.
432497825\H,-0.5657032668,5.6392894624,-1.907521016\H,1.6415656956,4.6
577312868,-2.4972194876\H,2.0977542387,2.2705583187,-2.1229328668\H,-4
.0913313724,2.7682026212,0.0423669069\N,-2.6228624921,4.0480849252,-0.
7881570904\H,0.383972101,0.7797195133,-1.1400690335\\Version=ES64L-G16
RevA.03\State=3-A\HF=-1043.3241454\S2=2.029102\S2-1=0.\S2A=2.000413\RM
SD=5.383e-09\RMSF=9.238e-07\Dipole=0.111931,-1.9412889,0.6099082\Quadr
upole=8.3159789,-9.7757245,1.4597456,6.3290663,6.5412559,0.7785389\PG=
C01 [X(C20H13N5)]\\@

2c, GS

1\1\GINC-GAUSSIAN-PRECISION-WORKSTATION-T7500\FOpt\RCAM-B3LYP\6-311G(d
,p)\C25H16N4\GAUSSIAN\01-Jan-2025\0\\#P CAM-B3LYP/6-311G(d,p) FOpt=tig

ht SCF=Direct freq(noraman) #P Geom=(NoDistance,NoAngle) SCRF(Solvent=
EthylEthanoate)\\benzotrazine carbazole\\0,1\N,0.1957414617,0.03871114
46,-1.9634484307\N,0.8019947499,2.7108489815,-2.1838279277\N,1.2055182
765,0.4232179163,-2.6599251985\C,1.499731933,1.7565476493,-2.750556002
\C,-2.4696090542,1.4972437108,0.0332133416\C,-1.6882807117,0.544041635
9,-0.555817059\C,-0.5674243187,0.9605385391,-1.3325236822\C,-0.2672692
96,2.3334005503,-1.4577838035\C,-1.0961092052,3.2953229314,-0.83409561
13\C,-2.1748685587,2.8746615198,-0.1145251128\C,-1.2327377505,-1.78053
45847,0.2441597361\C,-1.8889464079,-3.0225939672,0.1742400849\C,-0.031
8316468,-1.6263788225,0.9273888151\C,-1.3233008667,-4.1356298095,0.792
4383525\C,0.5101358514,-2.7461994875,1.5342526155\C,-0.1247148034,-3.9
915041335,1.468277447\H,-0.8490418452,4.3424068783,-0.9486867737\H,-2.
8192112128,3.5984373171,0.3684040514\H,-3.3221822757,1.1907666711,0.62
54487895\C,2.6899360671,2.1084779049,-3.5566739183\C,3.4516881065,1.11
72444159,-4.176095954\C,3.0596077952,3.4460093588,-3.7026402237\C,4.56
40472699,1.4615174428,-4.9282805128\H,3.163477925,0.0817807957,-4.0623
938162\C,4.1717566392,3.7853560047,-4.4548344781\H,2.4651092785,4.2103
301052,-3.2208396276\C,4.927094933,2.794146724,-5.0696660617\H,5.14958
07152,0.6854467331,-5.4058989254\H,4.4510195615,4.8263753739,-4.562611
0443\H,5.7969120182,3.0607318747,-5.6580081694\C,-3.1062302192,-2.8115
760545,-0.5807350181\C,-3.1369056282,-1.4486237479,-0.9296862608\N,-1.
9950712756,-0.8281350432,-0.427396544\C,-4.1447327747,-3.64779861,-0.9
832737273\H,-4.1345417638,-4.6999497588,-0.7238128865\C,-5.1877704091,
-3.1166619018,-1.7218969547\H,-6.0022098049,-3.7548078325,-2.041714763
2\C,-5.1997466889,-1.76028389,-2.0636459404\H,-6.0234227317,-1.3655149
461,-2.6462748724\C,-4.1789829042,-0.9085132978,-1.6755771853\H,-4.193
8770604,0.1389979963,-1.9474592992\H,-1.816707622,-5.0993804154,0.7463
590829\H,0.3279728814,-4.8475815231,1.953101711\H,1.4468444911,-2.6544
543846,2.0708134802\H,0.4666815019,-0.6674980148,0.9830941567\\Version
=ES64L-G16RevA.03\State=1-A\HF=-1180.9335696\RMSD=6.270e-09\RMSF=1.034
e-06\Dipole=-0.4621819,1.568585,0.4475161\Quadrupole=3.5910365,4.06907
73,-7.6601139,-0.820768,-3.8528611,0.0791753\PG=C01 [X(C25H16N4)]\\@

2c, S_1

1\1\GINC-GAUSSIAN-PRECISION-WORKSTATION-T7500\FOpt\RCAM-B3LYP TD-FC\6-
311G(d,p)\C25H16N4\GAUSSIAN\05-Jan-2025\0\\#P CAM-B3LYP/6-311G(d,p) Fo
pt TD=(singlets,root=1, NStates=3) SCF=tight #P Geom=(NoDistance,NoAng
le) SCRF(Solvent=EthylEthanoate)\\benzotrazine Carbazole TD ex\\0,1\N,
0.2117939469,0.0947280247,-1.9362134582\N,0.7961632318,2.7135258819,-2
.1683485306\N,1.1822906325,0.5067798269,-2.5952082795\C,1.5376435967,1
.7927411792,-2.7557933724\C,-2.5198822361,1.5261578913,0.0786676204\C,
-1.7249475109,0.5576914389,-0.5274438634\C,-0.6253698978,0.9605101791,
-1.2834214517\C,-0.2909086474,2.3485991984,-1.4310626437\C,-1.11683593
29,3.2818690681,-0.8095543299\C,-2.2185238001,2.8711428736,-0.06996666
65\C,-1.2490750754,-1.7685812533,0.2528740907\C,-1.8821760824,-3.02186
75563,0.1580718662\C,-0.0483337222,-1.6052624874,0.9353616177\C,-1.294
3500343,-4.1356594507,0.7534503056\C,0.5159407746,-2.7271345509,1.5182
202446\C,-0.0963130168,-3.9822681239,1.4290310778\H,-0.8740996131,4.33
01824624,-0.9202580613\H,-2.8451371791,3.6139945734,0.40655152\H,-3.36
98168315,1.2097673358,0.6689974735\C,2.7170338937,2.1141624424,-3.5650
147465\C,3.4690581296,1.1026250375,-4.161374257\C,3.0888815627,3.44598
14436,-3.7386379032\C,4.580542732,1.4221908794,-4.9224218523\H,3.17778
74732,0.0682861674,-4.0250468053\C,4.2023597285,3.7605804385,-4.501251
8613\H,2.4969033765,4.221713584,-3.2712267179\C,4.9498586175,2.7513265
142,-5.0939447632\H,5.1614048893,0.6330695014,-5.3836412541\H,4.487777
4108,4.7969421497,-4.6336204854\H,5.8199940997,2.9990391523,-5.6897191
523\C,-3.1029220456,-2.8172957325,-0.59327595\C,-3.1543261781,-1.44878
35072,-0.9187989218\N,-2.0266284326,-0.8177718384,-0.401048759\C,-4.12
80240904,-3.662196868,-1.0122328784\H,-4.1027118931,-4.7183545115,-0.7
70565217\C,-5.1774823042,-3.1340886089,-1.743950618\H,-5.9817971924,-3
.7788079788,-2.0760573665\C,-5.2092875033,-1.7722293886,-2.0631038348\

H,-6.0379924363,-1.3805150492,-2.6407104631\C,-4.2025053984,-0.9116758
092,-1.6585673784\H,-4.231068293,0.140347697,-1.9113954004\H,-1.769844
6699,-5.1074698207,0.690348572\H,0.3731537902,-4.8391697547,1.89617848
18\H,1.4518817109,-2.6290198908,2.0550523404\H,0.4290150399,-0.6368628
099,1.0106579124\\Version=ES64L-G16RevA.03\State=1-A\HF=-1180.9215825\
RMSD=5.993e-09\RMSF=3.256e-06\Dipole=0.3587305,0.9503436,-0.1934592\PG
=C01 [X(C25H16N4)]\\@

2c, T

1\1\GINC-GAUSSIAN-PRECISION-WORKSTATION-T7500\FOpt\UCAM-B3LYP\6-311G(d
,p)\C25H16N4(3)\GAUSSIAN\13-Jan-2025\0\\#P UCAM-B3LYP/6-311G(d,p) FOpt
=tight SCF=Direct freq(noraman) #P Geom=(NoDistance,NoAngle) SCRF(Solv
ent=EthylEthanoate)\\benzotrazine Carbazole t\\0,3\N,0.2105053393,0.09
6708258,-1.9456215338\N,0.8040950116,2.7170559592,-2.1748630524\N,1.19
27929935,0.4942408469,-2.6066107074\C,1.5296151724,1.7964947664,-2.757
6885893\C,-2.5005821375,1.5225316398,0.091179729\C,-1.7167889301,0.553
9465927,-0.5216045541\C,-0.6174446954,0.9556440156,-1.283805994\C,-0.2
846184631,2.337956823,-1.4259652314\C,-1.0997761735,3.2755051989,-0.79
86415788\C,-2.1975330427,2.870611591,-0.0532342753\C,-1.2544268905,-1.
772429706,0.2665406131\C,-1.8888223437,-3.0247923334,0.167777205\C,-0.
0602072375,-1.6095055122,0.960397523\C,-1.3086016591,-4.1383142467,0.7
711563701\C,0.4964214083,-2.7311521466,1.5510838812\C,-0.1171276967,-3
.9854091698,1.4583648957\H,-0.8525544421,4.3228513151,-0.9106887892\H,
-2.8197258064,3.6126133084,0.4300098686\H,-3.3485374529,1.2073129361,0
.6855372094\C,2.7092796977,2.1223459144,-3.5729093259\C,3.4562937801,1
.1116350608,-4.175016972\C,3.0824976887,3.4539257669,-3.7446056396\C,4
.5658087361,1.4308527537,-4.9404668343\H,3.1634890153,0.077694083,-4.0
402622733\C,4.1927467015,3.7687636703,-4.5109852125\H,2.4934877027,4.2
287241401,-3.2723324924\C,4.9360931446,2.7592327241,-5.1096591705\H,5.
1432065628,0.6419476736,-5.4063306504\H,4.4795861286,4.8048748897,-4.6
420645815\H,5.8040347287,3.0073359275,-5.7085499685\C,-3.1011029305,-2
.8194519206,-0.59698721\C,-3.1460687953,-1.4515923783,-0.926402845\N,-
2.0223644683,-0.821829071,-0.3990254893\C,-4.1240899629,-3.6628911523,
-1.024176456\H,-4.103825006,-4.7185327825,-0.7797968306\C,-5.165097171
4,-3.13389262,-1.7671740635\H,-5.9676758501,-3.7774413538,-2.105687944
4\C,-5.1908873066,-1.7725305538,-2.0892415928\H,-6.0133339841,-1.38013
06982,-2.675266068\C,-4.1861087888,-0.9134509397,-1.6768286897\H,-4.20
99684404,0.138286498,-1.9314584078\H,-1.7847590777,-5.109599222,0.7050
583394\H,0.3462439197,-4.8421250019,1.9318859535\H,1.427134655,-2.6335
255794,2.0970042407\H,0.4178209867,-0.6415560149,1.0385430747\\Version
=ES64L-G16RevA.03\State=3-A\HF=-1180.8651428\S2=2.029144\S2-1=0.\S2A=2
.000413\RMSD=6.768e-09\RMSF=5.429e-07\Dipole=0.3359503,0.8898672,-0.17
91427\Quadrupole=5.0386644,1.8127011,-6.8513655,0.4208301,-5.6912938,-
0.745427\PG=C01 [X(C25H16N4)]\\@

2d, GS

1\1\GINC-LOCALHOST\FOpt\RCAM-B3LYP\6-311G(d,p)\C27H20N4\PIOTR\28-Dec-2
024\0\\#P CAM-B3LYP/6-311G(d,p) FOpt=tight SCF=Direct freq(noraman) #P
 Geom=(NoDistance,NoAngle) fcheck SCRF(Solvent=EthylEthanoate)\\benzot
razine carbazole\\0,1\N,1.0447221391,0.1312077082,0.2983274198\N,3.177
6724868,-1.2339346347,-0.7702967612\N,2.2429508376,0.3826300627,0.6908
69278\C,3.2944038629,-0.2981345517,0.1402006823\C,-0.6702388695,-2.087
9406109,-2.0155888169\C,-0.4872666913,-1.0956641268,-1.0935638744\C,0.
8386092041,-0.8093603535,-0.6520200723\C,1.9309653093,-1.5238642727,-1
.1886295065\C,1.7064452762,-2.5428250884,-2.1432486144\C,0.4286120412,
-2.815506477,-2.5328352213\C,-1.8484457774,0.9709493709,-0.7272872729\
C,-3.064303619,1.2815782577,-0.0985147646\C,-1.1228917345,1.9406327063
,-1.4107948855\C,-3.5518772706,2.5878529131,-0.1421805763\C,-1.6318145
116,3.2252696713,-1.4389089448\C,-2.8403387953,3.5710945858,-0.8096184
065\H,2.5593004893,-3.0802247671,-2.5361029185\H,0.2393018341,-3.59314
23496,-3.2620917968\H,-1.6718903877,-2.3130919725,-2.3583791434\C,4.64

0272924,0.068392487,0.6352171085\C,4.8001693085,1.062870401,1.60052834
82\C,5.7666225547,-0.5853433547,0.134301695\C,6.0669274175,1.396095011
,2.054435517\H,3.9264258952,1.5672315225,1.9882659784\C,7.0302100829,-
0.2494672122,0.590784023\H,5.6367219888,-1.3562563034,-0.6130307201\C,
7.1839279857,0.7421801331,1.5520398205\H,6.1817934876,2.1695594229,2.8
03981201\H,7.8987079576,-0.7626960425,0.1962406758\H,8.1729048441,1.00
41625225,1.9085360213\C,-3.5639648635,0.051780103,0.4789414474\C,-2.63
11140968,-0.9507666174,0.1679055585\N,-1.5845552333,-0.3877890989,-0.5
619912188\C,-4.6997573716,-0.2679345914,1.2221896857\H,-5.4229996305,0
.5022471788,1.4673942126\C,-4.9045709309,-1.5693478797,1.6517827174\C,
-3.9499870966,-2.5485541789,1.3286361933\H,-4.1089122632,-3.5658229471
,1.6700084856\C,-2.8139462163,-2.2623007712,0.5939189231\H,-2.09324966
96,-3.0366705972,0.3644775492\H,-4.4898860948,2.8343659275,0.343434883
\H,-1.0780209109,3.9954911543,-1.964908969\H,-0.1886313109,1.705466079
9,-1.9036537335\C,-3.33818694,4.9919366497,-0.8686000035\H,-2.64222268
52,5.6761517259,-0.3762687451\H,-3.4492339915,5.3313097535,-1.90149182
25\H,-4.3068326002,5.09120853,-0.3774011352\C,-6.1229840299,-1.9440316
619,2.4551190986\H,-6.7610155703,-1.0773910666,2.6316942119\H,-6.72074
83791,-2.7003688186,1.939990961\H,-5.8446863849,-2.358629532,3.4273322
279\\Version=ES64L-G16RevC.01\State=1-A\HF=-1259.5368259\RMSD=3.274e-0
9\RMSF=1.054e-06\Dipole=0.2618266,-1.0907159,-1.0118635\Quadrupole=4.5
437054,2.4280606,-6.971766,0.4819093,-2.3581001,5.6812534\PG=C01 [X(C2
7H20N4)]\\@

2d, S_1

1\1\GINC-LOCALHOST\FOpt\RCAM-B3LYP TD-FC\6-311G(d,p)\C27H20N4\PIOTR\03
-Jan-2025\0\\#P CAM-B3LYP/6-311G(d,p) Fopt TD=(singlets,root=1, NState
s=3) SCF=tight #P Geom=(NoDistance,NoAngle) fcheck SCRF(Solvent=EthylE
thanoate)\\benzotrazine tBuCarbazole TD ex\\0,1\N,1.0663939262,0.11236
66502,0.2321406684\N,3.1634326709,-1.2371524747,-0.7834814367\N,2.2373
721493,0.3352548431,0.5854401655\C,3.3319587083,-0.2926878526,0.123381
6676\C,-0.7019791417,-2.1429530973,-2.0830515121\C,-0.5197377599,-1.13
38083923,-1.1411692234\C,0.772151146,-0.8435680098,-0.7042419099\C,1.9
104647944,-1.5498307653,-1.2207002404\C,1.6784326712,-2.5528365235,-2.
1582595691\C,0.3864509732,-2.8452938934,-2.5762418538\C,-1.860354273,0
.942465303,-0.7599381395\C,-3.0544136772,1.2716756246,-0.097606776\C,-
1.1276355914,1.9050032278,-1.4471669827\C,-3.5141028668,2.5883629854,-
0.1160786058\C,-1.6087646594,3.2009201609,-1.4475714506\C,-2.795468539
5,3.5643523483,-0.7875625769\H,2.5309264838,-3.0923767968,-2.548464396
5\H,0.2292830016,-3.6270604566,-3.3082140453\H,-1.7047128838,-2.362648
3411,-2.4253600178\C,4.6491059868,0.0921326731,0.6395466263\C,4.773924
6275,1.1076431071,1.5868871533\C,5.7875447632,-0.5649917526,0.17667759
35\C,6.0245998169,1.4601473077,2.0646797973\H,3.88731611,1.6168836748,
1.9443619588\C,7.0371949658,-0.2085560081,0.6581446549\H,5.6774832436,
-1.3512880418,-0.5583193721\C,7.1588048173,0.8031723614,1.6016671133\H
,6.1163808225,2.2496004226,2.8003415186\H,7.9192869087,-0.7221631262,0
.2959629485\H,8.1364052775,1.0805135224,1.9769386377\C,-3.5634803038,0
.0456169385,0.4796525132\C,-2.6546017411,-0.9700238451,0.1393661282\N,
-1.6196588687,-0.4213951727,-0.6151389152\C,-4.6871398367,-0.261810625
,1.2464907974\H,-5.3925642929,0.5175919488,1.513685576\C,-4.9025074577
,-1.5631014836,1.671265771\C,-3.9709201112,-2.5550042771,1.3197653323\
H,-4.1385439611,-3.5719704322,1.6579564425\C,-2.8477013311,-2.28168652
14,0.5609559929\H,-2.1438655845,-3.0643230999,0.3082337185\H,-4.435921
7361,2.8495906006,0.3923667012\H,-1.0507015748,3.96574293,-1.976998569
5\H,-0.2123559141,1.65261077,-1.9667691963\C,-3.2643631068,4.995927315
6,-0.8195695484\H,-2.5418736649,5.6605236323,-0.3386498255\H,-3.394531
1973,5.3482468287,-1.8458788717\H,-4.2179430738,5.1103873994,-0.302853
6999\C,-6.1092181572,-1.9252406921,2.497822029\H,-6.7205240623,-1.0472
273281,2.70947275\H,-6.7390367657,-2.6547182847,1.9820051943\H,-5.8171
977299,-2.3684112823,3.4532232847\\Version=ES64L-G16RevC.01\State=1-A\
HF=-1259.5248815\RMSD=6.820e-09\RMSF=3.497e-06\Dipole=0.6883264,-0.306

6262,-0.2003573\PG=C01 [X(C27H20N4)]\\@

2d, T

1\1\GINC-LOCALHOST\FOpt\UCAM-B3LYP\6-311G(d,p)\C27H20N4(3)\PIOTR\02-Ja
n-2025\0\\#P UCAM-B3LYP/6-311G(d,p) FOpt=tight SCF=Direct freq(noraman
) #P Geom=(NoDistance,NoAngle) fcheck SCRF(Solvent=EthylEthanoate)\\be
nzotrazine tBuCarbazole t\\0,3\N,1.0703153809,0.1070597988,0.239407177
6\N,3.1736108883,-1.2358338159,-0.7809796677\N,2.2415969432,0.34602474
3,0.6009997058\C,3.3292621425,-0.303804231,0.1249738179\C,-0.694766689
5,-2.1162108416,-2.100237093\C,-0.5177586981,-1.1194517495,-1.14925053
83\C,0.77547484,-0.8325487943,-0.7053215233\C,1.906845184,-1.534054075
9,-1.2246841383\C,1.6827463822,-2.5280906954,-2.1725243568\C,0.3951141
219,-2.8177282485,-2.5999149177\C,-1.8699538158,0.9500092455,-0.771053
961\C,-3.0628739431,1.2756742282,-0.1048856719\C,-1.1458139959,1.91276
89659,-1.4668475236\C,-3.5301771266,2.5896910086,-0.1285190047\C,-1.63
45423769,3.2058720138,-1.4722738989\C,-2.8204294126,3.5659621478,-0.80
89166881\H,2.5380312147,-3.0644823625,-2.5615573168\H,0.2379495423,-3.
5920161497,-3.3395711293\H,-1.6968283034,-2.3326034998,-2.4475811046\C
,4.6512756381,0.0734897875,0.6473383626\C,4.7751597325,1.0788163169,1.
6044594156\C,5.789661959,-0.581046479,0.1810033354\C,6.0257295582,1.42
54825628,2.0890208958\H,3.8886541064,1.5855621035,1.9653159704\C,7.038
3975935,-0.2316058935,0.6685796356\H,5.6793618073,-1.359875783,-0.5615
955219\C,7.159113203,0.7712711201,1.6223786021\H,6.1168971649,2.207518
8517,2.8326033837\H,7.9206854206,-0.7430155792,0.3038428269\H,8.136396
9863,1.0432044472,2.0025363698\C,-3.5609341369,0.0499070983,0.48252533
9\C,-2.6467173988,-0.961752098,0.1443880179\N,-1.6186626684,-0.4110454
639,-0.6174118273\C,-4.6791532468,-0.2606917204,1.2561526782\H,-5.3886
05361,0.5154664487,1.52209879\C,-4.8841553544,-1.5609896402,1.68887336
66\C,-3.9479310914,-2.5489642511,1.3382612974\H,-4.107863472,-3.565226
8072,1.6822460923\C,-2.8298677189,-2.2725391195,0.5731114802\H,-2.1222
959187,-3.0519779176,0.3207809141\H,-4.4508961911,2.8486529773,0.38308
82376\H,-1.0834113695,3.9709838217,-2.008490745\H,-0.2313618343,1.6627
126018,-1.9892719838\C,-3.2980568599,4.9945324875,-0.8470719158\H,-2.5
755634305,5.6667881651,-0.3769649312\H,-3.4389573817,5.3387763693,-1.8
747134317\H,-4.247972971,5.1069840047,-0.3232200776\C,-6.0848298463,-1
.926446016,2.5227468177\H,-6.7004491758,-1.0511363415,2.7331258138\H,-
6.7126301049,-2.662120487,2.0133050794\H,-5.7857259154,-2.3633492547,3
.478841545\\Version=ES64L-G16RevC.01\State=3-A\HF=-1259.4683366\S2=2.0
29014\S2-1=0.\S2A=2.00041\RMSD=5.912e-09\RMSF=7.092e-07\Dipole=0.64029
89,-0.2845218,-0.184359\Quadrupole=7.2453415,0.3810889,-7.6264304,2.20
90117,0.0740631,4.9930546\PG=C01 [X(C27H20N4)]\\@

2e, GS

1\1\GINC-LOCALHOST\FOpt\RCAM-B3LYP\6-311G(d,p)\C33H20N4\PIOTR\03-Jan-2
025\0\\#p freq=noraman cam-b3lyp/6-311g(d,p) scrf=(solvent=ethylethano
ate) fcheck fopt=tight geom=(nodistance,noangle) scf=direct\\benzotraz
ine Benzcarbazole\\0,1\N,-1.2274902664,-0.4160943673,-1.9979898379\N,-
3.4009395457,-2.0523917289,-2.4018113288\N,-2.1173436646,-0.1146441175
,-2.8750292133\C,-3.1906638572,-0.9421337146,-3.0672806254\C,-0.553221
9249,-3.0351027845,0.4318613386\C,-0.3872772298,-1.8970990937,-0.30135
00864\C,-1.3681212494,-1.5501938354,-1.2734790285\C,-2.4848754423,-2.3
867649751,-1.4741474809\C,-2.6316881766,-3.5610499783,-0.6971054055\C,
-1.6843091563,-3.8665204443,0.2337307856\C,0.8663778133,-0.1230446455,
0.9126299393\C,2.0660510808,0.576547839,0.7473324296\C,-0.0364380861,0
.1052067934,1.9703917635\C,2.2936480591,1.7163694342,1.5924185928\C,0.
2634477745,1.0868932865,2.8606541008\C,1.403321985,1.9249648348,2.6850
58945\H,-3.4982344297,-4.1864723848,-0.8664665918\H,-1.7835209897,-4.7
616848788,0.8343603137\H,0.1913781869,-3.3031075346,1.1703338936\C,-4.
1600874918,-0.5228543111,-4.1034949132\C,-3.9654155906,0.65182658,-4.8
307054295\C,-5.2842583348,-1.3083875535,-4.3610934531\C,-4.8821690906,
1.0316858694,-5.7986279415\H,-3.0940002206,1.2589402922,-4.6302047597\

C,-6.1973264275,-0.9250444579,-5.3290811324\H,-5.4301715817,-2.2179450
914,-3.7944141894\C,-5.9988871057,0.2460155997,-6.0503880757\H,-4.7233
982031,1.9452225162,-6.3584744379\H,-7.0670865252,-1.5410981741,-5.522
1988926\H,-6.7138662552,0.5450882138,-6.8074285767\C,2.7404896603,-0.0
376626191,-0.385069881\C,1.8484107655,-0.9805695387,-0.9050024681\N,0.
7326847543,-1.0529859402,-0.0981079005\C,4.0526521919,0.0286481096,-0.
9677962155\C,4.2839518084,-0.6955331938,-2.1728472577\C,3.2722369949,-
1.5410825866,-2.7156513626\H,3.4862884927,-2.0765655466,-3.6330902798\
C,2.0876319257,-1.7255044981,-2.0769030425\H,1.3420606296,-2.412257188
8,-2.4563708584\H,-0.3940105009,1.2809440108,3.6999668742\C,3.27594792
3,2.7065747829,1.3575305794\C,1.6143777057,3.0173053322,3.5565708007\C
,3.4378538864,3.7732266444,2.2037122823\C,2.6197423035,3.9190854176,3.
3366501894\H,2.7639106031,4.7564590258,4.0083514006\H,0.9409223017,3.1
421980233,4.3972975613\H,4.1939984579,4.5181602773,1.9858901915\H,3.88
20850625,2.6511412149,0.4671677838\C,5.1622165441,0.6773984592,-0.3775
086866\H,5.0566267433,1.1271917914,0.5970920807\C,5.552210136,-0.62941
06373,-2.7925376089\H,5.6983210768,-1.1658815369,-3.7235795144\C,6.390
2787094,0.6991199883,-0.9872190306\H,7.2199477375,1.1978497181,-0.5003
048152\C,6.5859296114,0.06542319,-2.2257559607\H,7.5548858722,0.100552
9856,-2.7085109832\H,-0.9332008808,-0.4927317936,2.0688311997\\Version
=ES64L-G16RevC.01\State=1-A\HF=-1488.1018554\RMSD=3.882e-09\RMSF=4.696
e-07\Dipole=-1.2123521,-1.739597,0.3800671\Quadrupole=0.3558294,1.1041
121,-1.4599415,8.6294772,-0.8471896,-0.8746341\PG=C01 [X(C33H20N4)]\\@

2e, S$_1$

1\1\GINC-LOCALHOST\FOpt\RCAM-B3LYP TD-FC\6-311G(d,p)\C33H20N4\PIOTR\07
-Jan-2025\0\\#P CAM-B3LYP/6-311G(d,p) Fopt TD=(singlets,root=1, NState
s=3) SCF=tight #P Geom=(NoDistance,NoAngle) fcheck SCRF(Solvent=EthylE
thanoate)\\benzotrazine carbazole TD ex\\0,1\N,-1.273806711,-0.4546940
751,-2.0399355961\N,-3.4095980087,-2.0575851946,-2.4029591899\N,-2.146
5017399,-0.2247578166,-2.895600641\C,-3.2361666755,-0.9733991156,-3.13
58990852\C,-0.5842117194,-3.0051763193,0.5267982482\C,-0.4068940582,-1
.8676916919,-0.2530549629\C,-1.3533355657,-1.5523278264,-1.2248745259\
C,-2.5014096147,-2.3852924786,-1.4410234488\C,-2.6416836335,-3.5124733
537,-0.6345390314\C,-1.6953787691,-3.8129946963,0.3367186594\C,0.87810
1003,-0.1128219772,0.9596290279\C,2.0786814997,0.5830489379,0.78286748
2\C,-0.0013854304,0.1002265391,2.040217041\C,2.3304203453,1.7054271918
,1.644508377\C,0.323398699,1.0622679884,2.9430110806\C,1.4645174922,1.
8972672846,2.7597371956\H,-3.5066132889,-4.143622187,-0.7886899712\H,-
1.824863675,-4.695602725,0.9495216437\H,0.158002926,-3.2461290182,1.27
650089\C,-4.1720477826,-0.56082061,-4.185824684\C,-3.9413818345,0.5947
327104,-4.9313626822\C,-5.3039536599,-1.3332742406,-4.4392635702\C,-4.
8348989131,0.9715456122,-5.9194462904\H,-3.060982736,1.1934809114,-4.7
320186648\C,-6.1952771921,-0.9516272124,-5.4292789099\H,-5.4715648272,
-2.2276345568,-3.8538590316\C,-5.9633967535,0.199639557,-6.1705593033\
H,-4.6518039188,1.869820416,-6.4959375003\H,-7.0738692893,-1.554540335
7,-5.6230853465\H,-6.6612590559,0.4965397145,-6.9440189379\C,2.7247030
564,-0.0123432402,-0.3760199352\C,1.8163089585,-0.9415813045,-0.893242
9703\N,0.7144605811,-1.0195350259,-0.0673932276\C,4.0239286788,0.05851
92078,-0.986628333\C,4.2250398704,-0.6452420991,-2.20918765\C,3.198512
8305,-1.4786672366,-2.7430719098\H,3.391147798,-2.0011654167,-3.672724
074\C,2.0271373942,-1.6687779785,-2.0818672391\H,1.2706892458,-2.34794
25577,-2.4531413806\H,-0.3146463834,1.2434123305,3.8000098368\C,3.3131
79231,2.6949735011,1.408574669\C,1.7004484215,2.9707523287,3.648039529
2\C,3.4992264219,3.7437626579,2.2720634737\C,2.7062001381,3.8713954318
,3.4248255569\H,2.8696301001,4.694148476,4.1100548456\H,1.0458709421,3
.0821522548,4.5054379782\H,4.2552444803,4.4885356793,2.0531924879\H,3.
900491492,2.6538385657,0.5049190011\C,5.1495519226,0.6919236406,-0.410
1174978\H,5.0681868345,1.1237520097,0.5748494674\C,5.4790628366,-0.572
1312041,-2.8565077539\H,5.6018322845,-1.0925212879,-3.7999634084\C,6.3
63525275,0.7202424641,-1.0471492526\H,7.2061940538,1.206574249,-0.5701

22767\C,6.5282612288,0.1086683122,-2.301214296\H,7.4860949563,0.148952
4404,-2.8053033225\H,-0.8992351916,-0.4950065512,2.1442156808\\Version
=ES64L-G16RevC.01\State=1-A\HF=-1488.0898268\RMSD=3.696e-09\RMSF=3.239
e-06\Dipole=-1.3202458,-0.7905591,-0.3827146\PG=C01 [X(C33H20N4)]\\@

2e, T

1\1\GINC-LOCALHOST\FOpt\UCAM-B3LYP\6-311G(d,p)\C33H20N4(3)\PIOTR\05-Ja
n-2025\0\\#P UCAM-B3LYP/6-311G(d,p) FOpt=tight SCF=Direct freq(noraman
) #P Geom=(NoDistance,NoAngle) fcheck SCRF(Solvent=EthylEthanoate)\\be
nzotrazine carbazole t\\0,3\N,-1.2418066682,-0.3592406421,-1.819263654
4\N,-3.3719266006,-2.0663027087,-2.4460446921\N,-2.2851910444,0.051899
386,-2.5777843141\C,-3.2685796523,-0.7953471375,-2.8466588933\C,-0.199
6319275,-3.4315583822,-0.098552173\C,-0.2115669858,-2.1277924111,-0.57
57770879\C,-1.265417952,-1.6317552311,-1.3738315568\C,-2.3398569614,-2
.5183717937,-1.6789008662\C,-2.310767548,-3.8278225147,-1.1916797523\C
,-1.2520940305,-4.2822004444,-0.4107664417\C,0.8258207273,-0.161874214
4,0.5464495169\C,2.066842185,0.5630526069,0.4770125453\C,-0.2228006769
,0.1867955945,1.3951391257\C,2.1419151849,1.8367117072,1.1677666413\C,
-0.0644132657,1.3049718771,2.1646335886\C,1.0804805876,2.1606382716,2.
0533548141\H,-3.1407567101,-4.478072422,-1.4413910093\H,-1.24882448,-5
.2997737364,-0.0405774521\H,0.6274630061,-3.7687838733,0.5144536332\C,
-4.3787557511,-0.2621688406,-3.689632626\C,-4.3420267002,1.0406232263,
-4.1884911156\C,-5.4773217083,-1.0650700892,-3.9934019373\C,-5.3782939
068,1.5262473988,-4.9706944914\H,-3.4881575258,1.6598120215,-3.9511257
165\C,-6.5146558366,-0.5783585079,-4.7760504551\H,-5.5000094875,-2.073
7129639,-3.6041946059\C,-6.4700128358,0.7191431266,-5.2682816756\H,-5.
3342461529,2.5402868694,-5.3511972269\H,-7.3618306832,-1.2155807877,-5
.0023994016\H,-7.2797472661,1.0996743342,-5.8799534451\C,2.9130669847,
-0.1931966005,-0.3435753686\C,2.1053211809,-1.259758449,-0.8726968058\
N,0.8773757529,-1.2458539482,-0.2799439609\C,4.3171412297,-0.168872965
6,-0.7081894327\C,4.733228249,-1.033471007,-1.7550379456\C,3.813670814
5,-1.963356616,-2.3412567673\H,4.1733242184,-2.5923119908,-3.146795310
2\C,2.5347105002,-2.1189061576,-1.8834429445\H,1.8644771977,-2.8611700
717,-2.2936869926\H,-0.8483446576,1.5961014014,2.8536972289\C,3.127447
4997,2.8031650541,0.9448622427\C,1.1072822678,3.3708589265,2.759718995
8\C,3.1209179847,4.0033070995,1.6336521108\C,2.1254746817,4.2809788456
,2.566540673\H,2.1331092947,5.2157872259,3.1121156369\H,0.2977967863,3
.593414249,3.4445505907\H,3.8945751208,4.734152434,1.4339791113\H,3.88
58579223,2.6383134195,0.1956939258\C,5.2983877817,0.5688938183,-0.0384
233803\H,5.0363281045,1.1521544238,0.8304853043\C,6.0747486883,-1.0388
259671,-2.1628057271\H,6.3707533453,-1.6895611243,-2.977075486\C,6.621
8007412,0.5278949503,-0.4394991469\H,7.3590387252,1.1066690866,0.10278
34979\C,7.0117693643,-0.2551728867,-1.5231559117\H,8.0460196733,-0.270
6911887,-1.8419354643\H,-1.1172242149,-0.4183906007,1.4380878318\\Vers
ion=ES64L-G16RevC.01\State=3-A\HF=-1488.0240448\S2=2.07007\S2-1=0.\S2A
=2.003009\RMSD=5.600e-09\RMSF=1.726e-06\Dipole=6.7342027,1.3352857,3.3
264235\Quadrupole=3.6174354,1.669811,-5.2872464,-2.0661057,-12.9106749
,14.1680263\PG=C01 [X(C33H20N4)]\\@

2f, GS

1\1\GINC-LOCALHOST\FOpt\RCAM-B3LYP\6-311G(d,p)\C25H16N4O1\PIOTR\28-Dec
-2024\0\\#P CAM-B3LYP/6-311G(d,p) FOpt=tight SCF=Direct freq(noraman)
#P Geom=(NoDistance,NoAngle) fcheck SCRF(Solvent=EthylEthanoate)\\benz
otrazine Phenoxazine\\0,1\N,0.5844556146,-0.0000041328,-0.3543197815\N
,2.7185110428,0.0000274424,1.3809242259\N,1.789857197,-0.0000099492,-0
.8017256415\C,2.8404296952,0.0000062224,0.0749422143\C,-1.1527499654,0
.0000485245,2.8355980299\C,-0.9602489758,0.000024287,1.4860944566\C,0.
3730107691,0.000016936,0.9820711201\C,1.4656505761,0.0000337565,1.8726
048934\C,1.2316598957,0.000058384,3.2694609067\C,-0.0510371686,0.00006
53669,3.7293510518\C,-2.4974178878,1.2097747526,0.0207751835\C,-3.5274
164342,1.1793548782,-0.9237443602\C,-1.9606879896,2.4438297118,0.36704

89125\C,-3.9971694018,2.3385245954,-1.5043888544\C,-2.4388322421,3.615
3746827,-0.2106997058\C,-3.4559013208,3.5687213216,-1.1469976693\H,2.0
845516127,0.0000713784,3.9354083414\H,-0.244276812,0.00008388,4.794640
2118\H,-2.1636357122,0.000054012,3.2225275825\C,4.1924714552,-0.000000
7771,-0.5268813054\C,4.3586865382,-0.0000222885,-1.9121782008\C,5.3188
014981,0.0000142987,0.2967829786\C,5.6317401902,-0.0000288071,-2.46088
60657\H,3.4855112395,-0.0000332091,-2.5492294616\C,6.5886290157,0.0000
076658,-0.2557620689\H,5.1839111391,0.0000312507,1.369852552\C,6.74865
38243,-0.0000139897,-1.6361082741\H,5.751610751,-0.0000453848,-3.53738
74865\H,7.4570426407,0.0000195372,0.3916748927\H,7.7424734243,-0.00001
92337,-2.0674730663\C,-3.5274155465,-1.1794031599,-0.9236979288\C,-2.4
974166163,-1.2097848126,0.0208223092\N,-2.063428269,0.0000063024,0.591
0332878\O,-4.1327039679,-0.0000316278,-1.2927753283\C,-3.9971684396,-2
.3385963959,-1.504295578\H,-4.7947912174,-2.2584155004,-2.2320852507\C
,-3.4559004264,-3.5687786568,-1.1468544392\H,-3.8315245043,-4.47620740
61,-1.6018147992\C,-2.4388311588,-3.6153940904,-0.210554778\H,-2.00437
47685,-4.5635596176,0.0803513924\C,-1.9606864803,-2.443825617,0.367145
7834\H,-1.1662073613,-2.4929958939,1.0993078917\H,-4.794791684,2.25831
41508,-2.232175809\H,-3.8315249101,4.4761315616,-1.6019953448\H,-2.004
3754619,4.5635519746,0.0801675154\H,-1.166208397,2.4930296762,1.099208
4644\\Version=ES64L-G16RevC.01\State=1-A\HF=-1256.1297753\RMSD=4.256e-
09\RMSF=5.784e-07\Dipole=0.7668215,0.0000319,1.7613802\Quadrupole=-1.5
072404,-3.5902561,5.0974966,0.0000298,-0.6201796,0.0001115\PG=C01 [X(C
25H16N4O1)]\\@

2f, S_1

1\1\GINC-LOCALHOST\FOpt\RCAM-B3LYP TD-FC\6-311G(d,p)\C25H16N4O1\PIOTR\
02-Jan-2025\0\\#P CAM-B3LYP/6-311G(d,p) Fopt TD=(singlets,root=1, NSta
tes=3) SCF=tight #P Geom=(NoDistance,NoAngle) fcheck SCRF(Solvent=Ethy
lEthanoate)\\benzotrazine phenoxazine TD ex\\0,1\N,0.5559872625,-0.000
0034152,-0.4102516658\N,2.7308375917,0.0000275792,1.3389074143\N,1.828
9314091,-0.000009931,-0.8676066903\C,2.8259006702,0.0000043355,0.00683
27035\C,-1.1784415234,0.000047607,2.8261747447\C,-0.9278970009,0.00002
46679,1.4567132746\C,0.3765987673,0.0000177414,0.9197335638\C,1.470115
8199,0.0000350315,1.8368282043\C,1.2093807578,0.0000580841,3.211816512
2\C,-0.0968252251,0.0000643461,3.6950701311\C,-2.4724817732,1.19332821
56,0.0016978848\C,-3.4601085264,1.1692087765,-0.9987297493\C,-1.999042
1214,2.4348301215,0.4520827081\C,-3.9695749645,2.3412225615,-1.5330050
768\C,-2.5055526707,3.5948321798,-0.0821655587\C,-3.4917063624,3.55083
50214,-1.0745169306\H,2.0545902747,0.0000708637,3.8883688837\H,-0.2736
101789,0.0000825729,4.7633509833\H,-2.196302284,0.0000520941,3.1933632
495\C,4.2024851431,-0.0000024384,-0.570966927\C,4.4026274672,-0.000026
8891,-1.9516395905\C,5.3147152727,0.0000154235,0.2689466071\C,5.685523
0154,-0.0000337119,-2.4773597445\H,3.5378919773,-0.0000405399,-2.60071
88692\C,6.5983713931,0.0000087158,-0.2578466747\H,5.1526628993,0.00003
43639,1.3380950918\C,6.7892333526,-0.0000159646,-1.6330082907\H,5.8253
768913,-0.0000529922,-3.5521429202\H,7.4530425767,0.0000227423,0.40869
32678\H,7.7915157356,-0.0000213039,-2.0451894713\C,-3.4601069001,-1.16
92592309,-0.9986837938\C,-2.4724799621,-1.1933380219,0.0017446069\N,-1
.9994278113,0.00000541,0.5085446316\O,-3.9457404285,-0.0000349259,-1.4
754352132\C,-3.9695717586,-2.3412947025,-1.5329130403\H,-4.7271942628,
-2.2752605686,-2.3016888428\C,-3.4917014289,-3.5508884982,-1.074377452
5\H,-3.8818589922,-4.470859605,-1.4887558826\C,-2.5055474938,-3.594845
315,-0.0820245508\H,-2.1344892303,-4.5488750776,0.2668453995\C,-1.9990
384366,-2.4348215656,0.4521780645\H,-1.2362959703,-2.4578904415,1.2154
705811\H,-4.7271972562,2.275157182,-2.3017784039\H,-3.8818650758,4.470
7893199,-1.4889315942\H,-2.1344958049,4.5488761736,0.2666669591\H,-1.2
362998342,2.4579299982,1.2153744662\\Version=ES64L-G16RevC.01\State=1-
A\HF=-1256.1169692\RMSD=8.953e-09\RMSF=6.084e-06\Dipole=-5.7481995,-0.
0000177,-0.2310034\PG=C01 [X(C25H16N4O1)]\\@

2f, T

1\1\GINC-LOCALHOST\FOpt\UCAM-B3LYP\6-311G(d,p)\C25H16N4O1(3)\PIOTR\04-
Jan-2025\0\\#P UCAM-B3LYP/6-311G(d,p) FOpt=tight SCF=Direct freq(noram
an) #P Geom=(NoDistance,NoAngle) fcheck SCRF(Solvent=EthylEthanoate)\\
benzotrazine phenoxazine triplet\\0,3\N,0.6162304516,0.0000503265,-0.4
604317588\N,2.7668335063,0.0000277619,1.3341922815\N,1.9006693419,0.00
00695109,-0.887924557\C,2.8833937194,0.0000568806,0.0016592165\C,-1.16
23458564,-0.0000467396,2.7535745075\C,-0.8959506925,-0.000020947,1.391
8334131\C,0.4128825876,0.000010018,0.8714765022\C,1.4902541893,0.00000
39194,1.8051713671\C,1.2122170917,-0.0000253337,3.1753768346\C,-0.0979
486009,-0.0000487222,3.6454766617\C,-2.4868080869,1.191472465,-0.03192
5765\C,-3.5472864312,1.1687270614,-0.9547975861\C,-1.9777649204,2.4348
546374,0.3777670873\C,-4.0957216839,2.3421261716,-1.4523167261\C,-2.52
10916161,3.5923050298,-0.1203019391\C,-3.5829961026,3.5502074818,-1.03
5061399\H,2.0513988529,-0.0000287387,3.8604546686\H,-0.2919663235,-0.0
000674193,4.7105808268\H,-2.1870870532,-0.0000617827,3.1039973861\C,4.
2685191074,0.0000671052,-0.5526225679\C,4.4929391485,0.000052631,-1.93
00226016\C,5.3668413701,0.0000856141,0.3061389141\C,5.7844406482,0.000
0548348,-2.4336173802\H,3.6389189438,0.0000428923,-2.5931129711\C,6.65
92574894,0.0000893922,-0.1987735238\H,5.1862533564,0.0000961191,1.3723
447931\C,6.8738227409,0.0000724423,-1.5705034369\H,5.9426586593,0.0000
431867,-3.5059187796\H,7.5024658771,0.0001043633,0.4823156593\H,7.8830
367605,0.0000740057,-1.9655196159\C,-3.5473647416,-1.1687900386,-0.954
6998839\C,-2.486892616,-1.191529631,-0.0318200397\N,-1.9859288851,-0.0
000249747,0.4442414911\O,-4.0727101413,-0.0000320688,-1.389024109\C,-4
.0958723377,-2.342193053,-1.4521295162\H,-4.9111446901,-2.2743691121,-
2.1596317643\C,-3.5832279525,-3.5502748184,-1.0347757839\H,-4.00148425
68,-4.4712934571,-1.4188993102\C,-2.5213348713,-3.5923688556,-0.120003
2865\H,-2.1217729055,-4.5467836941,0.1959126095\C,-1.9779369496,-2.434
913957,0.3779784123\H,-1.1579849732,-2.4582599276,1.0804223413\H,-4.91
10044193,2.2742994845,-2.1598068101\H,-4.0011974278,4.4712233751,-1.41
92515405\H,-2.1214664987,4.5467192147,0.1955360453\H,-1.1578078082,2.4
582033461,1.0802046332\\Version=ES64L-G16RevC.01\State=3-A\HF=-1256.07
07528\S2=2.048333\S2-1=0.\S2A=2.001517\RMSD=3.779e-09\RMSF=5.855e-07\D
ipole=-6.026714,-0.0001148,-0.1240784\Quadrupole=1.780444,9.2791116,-1
1.0595557,0.0011521,6.7721136,-0.001041\PG=C01 [X(C25H16N4O1)]\\@

nitroxide

1\1\GINC-LOCALHOST\FOpt\UB3LYP\6-311G(d,p)\C16H17N2O1(2)\PIOTR\08-Apr-
2025\0\\#P UB3LYP/6-311G(d,p) Fopt=tight geom=(nodistance,noangle) SCF
=direct\\carbazole-NO-tBu\\0,2\C,-1.2316547433,1.5697056842,-0.0160268
252\C,-1.0240127755,0.1734201382,-0.1467433467\C,-0.1690304832,2.47275
52204,-0.0733127284\C,0.2702907539,-0.3182223142,-0.3294894011\C,1.108
2003984,1.9643798118,-0.246948999\C,1.3366643559,0.5776655915,-0.35789
32441\C,-2.3296092854,-0.4511437567,-0.0700120244\C,-3.2707008954,0.59
51952475,0.1094672951\N,-2.5865919379,1.8001729564,0.1420684708\C,-2.7
813479946,-1.7727011659,-0.132797536\H,-2.0798832066,-2.5883568085,-0.
2693456012\C,-4.1421106183,-2.0292463568,-0.0191389755\H,-4.5038550555
,-3.0496003928,-0.0673862124\C,-5.0579876623,-0.980119899,0.1574700323
\H,-6.1150273255,-1.2049193258,0.2432104814\C,-4.6375016215,0.34332298
36,0.2247595538\H,-5.3499773676,1.1491949392,0.3613365917\H,0.43902760
44,-1.3762592146,-0.4756205066\H,1.9595420826,2.627073376,-0.323766908
1\H,-0.3297077532,3.5415773948,0.0120454834\H,-3.0126715854,2.70607395
45,0.2393705013\N,2.6859884373,0.1385242652,-0.5922092264\O,3.38791403
32,0.8781986438,-1.3713887286\C,3.4893487419,-0.7006699595,0.385108296
8\C,2.6144937747,-1.6672904805,1.188715609\H,1.8478445238,-1.150755287
7,1.7677078165\H,2.1328974122,-2.4093572523,0.5485260463\H,3.256126472
,-2.2067647194,1.8899137208\C,4.1978799419,0.2811525758,1.337094962\H,
3.4701696875,0.8426684089,1.9293552546\H,4.8506117516,-0.2626654182,2.
0249842156\H,4.80028287,0.9861232607,0.7628596448\C,4.5138123288,-1.48
7373243,-0.4437427282\H,5.1708585616,-2.0537680748,0.2210120793\H,4.01

01593557,-2.1898007058,-1.1129868832\H,5.1151652237,-0.809371077,-1.04
71671802\\Version=ES64L-G16RevC.01\State=2-A\HF=-804.8230124\S2=0.7554
37\S2-1=0.\S2A=0.750024\RMSD=9.280e-09\RMSF=5.024e-07\Dipole=-0.957072
1,0.1092644,0.941295\Quadrupole=1.3953651,7.4916347,-8.8869998,-8.4612
26,5.0248205,2.2573305\PG=C01 [X(C16H17N2O1)]\\@

carb-*N*-CPh$_2$

1\1\GINC-LOCALHOST\FOpt\UB3LYP\6-311G(d,p)\C25H18N1(2)\PIOTR\09-Apr-20
25\0\\#P UB3LYP/6-311G(d,p) Fopt=tight geom=(nodistance,noangle) SCF=d
irect\\carbazole-CPh2\\0,2\C,-1.1505803895,1.1204874438,0.9020381819\C
,-1.0073018303,-0.0316961999,0.0938416895\C,-0.0865083659,1.626377758,
1.6483674396\C,0.2307778886,-0.6762894661,0.0249263461\C,1.1352892887,
0.9674956116,1.5615534567\C,1.2976404161,-0.1702641636,0.757218464\C,-
2.3014060987,-0.2924635303,-0.502515807\C,-3.182809262,0.7071631246,-0
.028059921\N,-2.4797441153,1.5712267791,0.8288501331\C,-2.7718064864,-
1.2599861265,-1.3945450878\H,-2.1075743324,-2.0308521499,-1.7693273466
\C,-4.1002316652,-1.2167411152,-1.7994216683\H,-4.4785517831,-1.961453
4831,-2.4900370694\C,-4.9579618206,-0.2123515713,-1.3268623119\H,-5.98
97414548,-0.192554697,-1.6590968274\C,-4.5141994687,0.7626303743,-0.43
9767839\H,-5.1822076642,1.5342807343,-0.0806750702\H,0.3554973321,-1.5
621986491,-0.5878630685\H,1.9785932162,1.3434894058,2.1297156002\H,-0.
2033729006,2.5029469532,2.2717417213\H,2.263631898,-0.6596598047,0.712
2132493\C,-3.0148198802,2.6948695042,1.4986341729\C,-2.3914943138,3.98
14522728,1.2540367925\C,-1.7167981512,4.231982922,0.0357671897\C,-2.40
4584066,5.0173640479,2.2182158002\C,-1.1146858582,5.4565345693,-0.2104
862531\H,-1.6876597702,3.4572905729,-0.7201122298\C,-1.7953728023,6.23
81450219,1.9648761876\H,-2.8704135227,4.8428419143,3.1797042647\C,-1.1
505289898,6.4702416118,0.7488311099\H,-0.6157106691,5.6248766154,-1.15
8341643\H,-1.8115429921,7.0107302484,2.7257314453\H,-0.6760753809,7.42
51226874,0.5543940725\C,-4.1462009244,2.4751810785,2.3791900799\C,-5.1
064565305,3.4832563366,2.6333979214\C,-4.3411351206,1.2171838558,2.996
9279523\C,-6.1850167181,3.2480905103,3.4742447058\H,-5.0141180168,4.44
21038514,2.1394344285\C,-5.4194479679,0.9926262539,3.8392644077\H,-3.6
243225469,0.4254153353,2.8190715292\C,-6.3483620257,2.0048650885,4.087
5761743\H,-6.9115989713,4.0352416418,3.6431272345\H,-5.5365912081,0.02
24658003,4.3091700768\H,-7.1918669552,1.8249760913,4.7439674856\\Versi
on=ES64L-G16RevC.01\State=2-A\HF=-1018.4866173\S2=0.774948\S2-1=0.\S2A
=0.750553\RMSD=8.613e-09\RMSF=5.165e-07\Dipole=-0.1860129,0.390618,0.2
328475\Quadrupole=0.9785687,2.3764254,-3.3549941,0.2100591,-2.8620039,
3.0927713\PG=C01 [X(C25H18N1)]\\@

***N*-oxide**

1\1\GINC-LOCALHOST\FOpt\UB3LYP\6-311G(d,p)\C12H8N1O1(2)\PIOTR\09-Apr-2
025\0\\#p ub3lyp/6-311g(d,p) fopt=tight geom=(nodistance,noangle) scf=
direct\\carbazole-N-oxide\\0,2\C,0.,-1.144639235,0.6066100306\C,0.,-0.
7311101185,-0.7415799322\C,0.,-2.4761930236,0.9948882647\C,0.,-1.70068
76283,-1.740783295\C,0.,-3.4287586143,-0.0217959879\C,0.,-3.0463331357
,-1.3700326662\C,0.,0.7311101185,-0.7415799322\C,0.,1.144639235,0.6066
100306\N,0.,0.,1.4317737058\C,0.,1.7006876283,-1.740783295\H,0.,1.4209
847893,-2.78800789\C,0.,3.0463331357,-1.3700326662\H,0.,3.8106686561,-
2.1383776299\C,0.,3.4287586143,-0.0217959879\H,0.,4.4817777759,0.23410
40245\C,0.,2.4761930236,0.9948882647\H,0.,2.7423241926,2.0439191984\H,
0.,-1.4209847893,-2.78800789\H,0.,-4.4817777759,0.2341040245\H,0.,-2.7
423241926,2.0439191984\H,0.,-3.8106686561,-2.1383776299\O,0.,0.,2.6978
987203\\Version=ES64L-G16RevC.01\State=2-B1\HF=-592.1593573\S2=0.76106
7\S2-1=0.\S2A=0.750102\RMSD=5.480e-09\RMSF=1.767e-06\Dipole=0.,0.,-1.0
849064\Quadrupole=-5.916299,8.1246499,-2.2083509,0.,0.,0.\PG=C02V [C2(
N1O1),SGV(C12H8)]\\@

12. References

1. G. R. Fulmer, A. J. M. Miller, N. H. Sherden, H. E. Gottlieb, A. Nudelman, B. M. Stoltz, J. E. Bercaw and K. I. Goldberg, *Organometallics*, 2010, **29**, 2176–2179.
2. P. Kaszyński, C. P. Constantinides and V. G. Young, Jr., *Angew. Chem. Int. Ed.*, 2016, **55**, 11149–11152.
3. CrysAlis PRO. 2014, Agilent Technologies Ltd, Yarnton, Oxfordshire, England.
4. G. Sheldrick, *Acta Cryst. A*, 2015, **71**, 3-8.
5. G. Sheldrick, *Acta Cryst. C*, 2015, **71**, 3-8.
6. N. G. Connelly and W. E. Geiger, *Chem. Rev.*, 1996, **96**, 877-910.
7. C. Hansch, A. Leo and R. W. Taft, *Chem. Rev.*, 1991, **91**, 165–195.
8. P. Bartos, M. Celeda, A. Pietrzak and P. Kaszyński, *Org. Chem. Front.*, 2022, **9**, 929–938.
9. P. Bartos, B. Anand, A. Pietrzak and P. Kaszyński, *Org. Lett.*, 2020, **22**, 180-184.
10. B. Bleaney and K. D. Bowers, *Proc. R. Soc. London, Ser. A.*, 1952, **214**, 451–465.
11. O. Kahn, *Molecular Magnetism*, VCH Publishers, New York, NY, 1993.
12. Gaussian 16, M. J. Frisch, G. W. Trucks, H. B. Schlegel, G. E. Scuseria, M. A. Robb, J. R. Cheeseman, G. Scalmani, V. Barone, G. A. Petersson, H. Nakatsuji, X. Li, M. Caricato, A. V. Marenich, J. Bloino, B. G. Janesko, R. Gomperts, B. Mennucci, H. P. Hratchian, J. V. Ortiz, A. F. Izmaylov, J. L. Sonnenberg, D. Williams-Young, F. Ding, F. Lipparini, F. Egidi, J. Goings, B. Peng, A. Petrone, T. Henderson, D. Ranasinghe, V. G. Zakrzewski, J. Gao, N. Rega, G. Zheng, W. Liang, M. Hada, M. Ehara, K. Toyota, R. Fukuda, J. Hasegawa, M. Ishida, T. Nakajima, Y. Honda, O. Kitao, H. Nakai, T. Vreven, K. Throssell, J. A. Montgomery, Jr., J. E. Peralta, F. Ogliaro, M. J. Bearpark, J. J. Heyd, E. N. Brothers, K. N. Kudin, V. N. Staroverov, T. A. Keith, R. Kobayashi, J. Normand, K. Raghavachari, A. P. Rendell, J. C. Burant, S. S. Iyengar, J. Tomasi, M. Cossi, J. M. Millam, M. Klene, C. Adamo, R. Cammi, J. W. Ochterski, R. L. Martin, K. Morokuma, O. Farkas, J. B. Foresman, and D. J. Fox, Gaussian, Inc., Wallingford CT. 2016,.
13. M. Cossi, G. Scalmani, N. Rega and V. Barone, *J. Chem. Phys.*, 2002, **117**, 43–54.
14. F. De Vleeschouwer, A. Chankisjijev, W. Yang, P. Geerlings and F. De Proft, *J. Org. Chem.*, 2013, **78**, 3151–3158.
15. R. E. Stratmann, G. E. Scuseria and M. J. Frisch, *J. Chem. Phys.*, 1998, **109**, 8218-8224.

16. K. Yamaguchi, Y. Takahara, T. Fueno and K. Nasu, *Jpn. J. Appl. Phys.*, 1987, **26**, L1362-L1364.

17. C. P. Constantinides, A. A. Berezin, M. Manoli, G. M. Leitus, M. Bendikov, J. M. Rawson and P. A. Koutentis, *New J. Chem.*, 2014, **38**, 949-954.
